# Low-Valent Tungsten Catalyzed Carbonylative Synthesis of Benzoates from Aryl Iodides and Alcohols

**DOI:** 10.3390/molecules29225305

**Published:** 2024-11-10

**Authors:** Feihua Ye, Lin Lu, Zhaoyang Huang, Yunwei Huang, Lixuan Huang, Chunsheng Li, Xiang Li

**Affiliations:** School of Environmental and Chemical Engineering, Zhaoqing University, Zhaoqing 526061, China; yefeihua@zqu.edu.cn (F.Y.); lulin207115@163.com (L.L.);

**Keywords:** tungsten catalyst, carbonylation, benzoates, oxidative addition, synthetic method

## Abstract

Non-noble metals catalyzed carbonylative reactions serve as straightforward and sustainable methods for the synthesis of functionalized carbonyl-containing compounds. Herein, a low-valent-tungsten-catalyzed reaction that enables the coupling of aryl iodides and alcohols or phenols was disclosed, employing the readily available W(CO)_6_ as the effective catalyst and PPh_3_ as ligand. Under the optimal reaction conditions, aryl iodides smoothly underwent carbonylative coupling reactions with alcohols or phenols, processing the feature of broad substrate scope and good functional groups tolerance. Furthermore, this conversion can be carried out on a gram scale, showcasing significant promise in the synthesis of pharmaceutical or biologically active compounds.

## 1. Introduction

Carbonyl-containing compounds, including aldehydes, ketones, esters, amides, and carboxylic acids, are important and versatile structure motifs for the construction of functionalized materials, pharmaceuticals, and other biologically active molecules [1,2,3]. Owing to their significance, numerous methodologies have been developed in recent years. Among them, the transition metal-catalyzed carbonylation reaction represents an atom-efficient toolbox to convert a variety of easily available substrates into valuable carbonylated products [4,5,6,7,8,9,10]. Heck et al. accomplished pioneering work in the field of palladium-catalyzed carbonylation reactions. Since then, palladium catalyst systems have emerged as the most straightforward choices for the synthesis of carbonyl-containing compounds [11,12,13]. While palladium catalyst systems demonstrated enhanced reactivity and efficiency, the high cost associated with them, along with the necessity for expensive phosphine ligands, has constrained their broader utilization, particularly on an industrial scale [4]. In this regard, other noble (such as Rh, Ru) and especially non-noble metals (Fe, Co, Ni) emerged as attractive alternatives [14,15,16,17,18,19,20]. For example, Shi et al. reported a cobalt-catalyzed enantioselective C-H carbonylation reaction utilizing carbon monoxide as the C1 building block, which facilitates the highly efficient production of chiral isoindolinones [17]. Moreover, an innovative four-component carbonylation process for synthesizing *β*, *γ*-unsaturated ketones has been established by Wu’s group through the cross-coupling of 1,3-butadiene, alkyl bromides, and arylboronic acids in the presence of carbon monoxide at 1 bar pressure, using nickel as the catalyst. This transformation possessed the advantages of high synthetic efficiency, gentle reaction conditions, and outstanding 1,4-regioselectivity [18]. Therefore, due the huge successes that can be achieved, developing more non-noble metal-catalyzed carbonylation reactions for the synthesis of functional carbonyl compounds is in high demand.

**Scheme 1:**
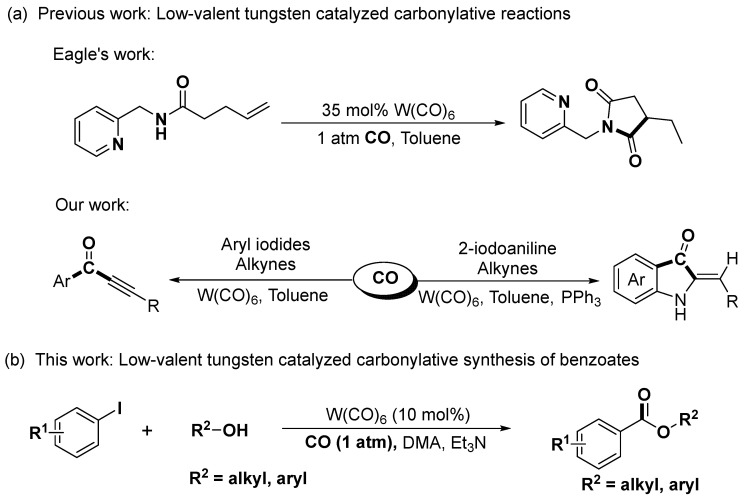
Overview of tungsten-catalyzed carbonylation reactions.

Non-noble metal tungsten catalysts have been extensively investigated as efficient catalysts and functional materials in contemporary organic chemistry. Accordingly, the high-valent tungsten-catalyzed alkyne metathesis and polymerization reactions have been well-established in recent years [21]. Likewise, low-valent tungsten catalysts also displayed remarkably distinct catalytic characteristics in organic synthesis. To date, low valent tungsten catalysts have been proven to be effective in alkene isomerization [22], hydrogenation [23], allylic substitution reactions [24], oxidative cross dehydrogenative coupling reactions [25], hydroboration reaction [26], and carbonylation reaction [27], indicating the appealing and promising reactivity of these catalysts. For instance, as shown in Scheme 1, in 2021, Eagle’s group revealed that the tungsten catalytic system could achieve the hydroboration of unactivated alkenes at distal C(sp^3^)-H bonds aided by native directing groups. This reaction is distinguished by its simplicity, exquisite regio- and chemoselectivity, and wide substrate scope [22]. Subsequently, Song et al. successfully applied low-valent-tungsten catalysis for the direct hydroboration of esters and nitriles with excellent substrate scope. Aside from these innovative transformations, Eagle’s group discovered a comparatively mild tungsten-catalyzed isomerization and tandem carbonylation of alkenes. Driven by our ongoing dedication to developing efficient synthesis techniques for the construction of carbonyl compounds, our group also accomplished direct synthesis of functionalized alkynones and indoxyls compounds via tungsten-catalyzed carbonylative sonogashira coupling reactions [27]. Although significant advancements have been actualized, the exploration of more effective examples of the non-noble tungsten-catalyzed carbonylation reactions is still desirable and appealing. Herein, as a part of our continuous efforts to broaden the scope of the utilization of non-noble tungsten catalysts in carbonylation reactions, we disclosed a tungsten-catalyzed carbonylative reaction of aryl iodides and alcohols or phenols with the atmospheric CO as the CO surrogates, obtained after tremendous effort. Compared with classic Pd catalysts, the W catalysts did not display superior performance in the carbonylative reaction. Despite this, and considering the importance of the carbonyl-containing molecules, our reaction could provide alternatives for the construction of these compounds.

## 2. Results and Discussion

At the outset, we assessed the feasibility of this approach by probing the reactivity of iodobenzene (**1a**) in conjunction with *n*-butanol (**2a**) with the employment of W(CO)_6_ as the catalyst (Table 1). Pleasingly, the expected product **3a** could be attained at a 69% yield. We then switched our focus to investigating the capabilities of other transition metal catalysts. Upon performing this reaction with several different tungsten catalysis, such as W(CO)_6_, W(CH_3_CN)_3_(CO)_3_, W(COD)_2_(CO)_4_, and Mo(CO)_6_ in Toluene at 80 °C, the product **3a** was produced as anticipated (entries 1–4). Among these catalysts, W(CO)_6_ exhibited the highest catalytic efficiency. To enhancing the productivity of this reaction, a range of ligands were evaluated under identical conditions, and PPh_3_ was determined as the optimal choice (entries 5–9). Subsequent optimization concentrated on assessing various kinds of solvents. We found that the solvent did not significantly affect the transformation of the model substrates. Only in DMF (*N*,*N*-Dimethylformamide) was the yield of **3a** decreased significantly (entries 10–17). In DMA (*N*,*N*-Dimethylacetamide), product **3a** could be obtained at an 80% yield. Control experiments indicated that both the tungsten catalyst and ligand were crucial for this conversion to proceed successfully (entries 18–19, respectively). Finally, the moderation of the reaction temperature from 80 °C to 100 °C established a platform for the tungsten-catalyzed carbonylative synthesis of benzoates (entries 20–21).

**Scheme 2:**
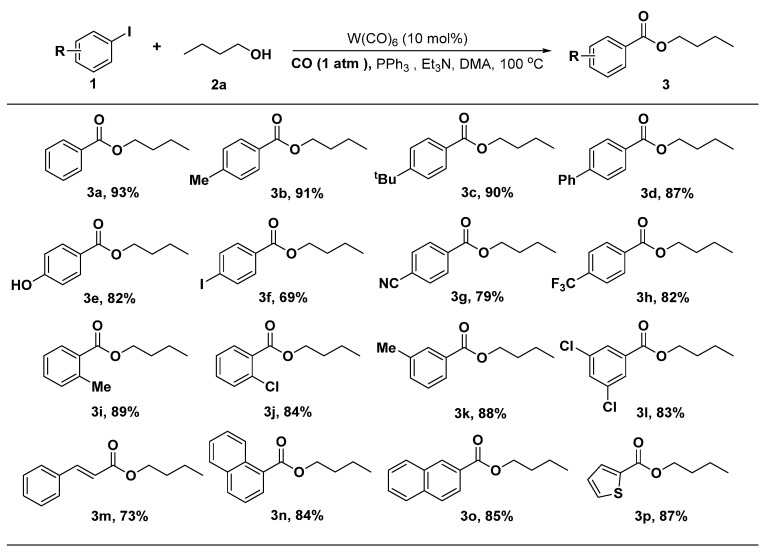
Substrate Scope of Carbonylative Synthesis of Benzoates. aryl iodides **1** (0.5 mmol), *n*-butanol **2a** (0.75 mmol), PPh_3_ (15 mol%), CO balloon, W(CO)_6_ (10 mol%), Et_3_N (2 equiv.), DMA (2 mL), 100 °C, 24 h.

After establishing the optimal reaction conditions of this carbonylative reaction, the investigation of the versatility of aryl iodides and alcohols or phenols was carried out accordingly. Initially, we assessed the reactivity of substituted aryl iodides, as depicted in Scheme 2. Encouragingly, aryl iodide substrates with electron-donating and electron-withdrawing groups effectively underwent the reaction process, yielding the corresponding products with good yields (**3a**–**3l**). Delightfully, halogen substituents (such as I) were well-tolerated in this transformation, which allowed the subsequent structural diversification of benzoates. Furthermore, aryl iodides with sterically hindered groups at the 2-position were also well accommodated within the reaction system, resulting in products with good yields (**3i**–**3j**). Except for these iodobenzene derivatives, fused-ring and heterocyclic substrates did not notably impact the efficiency of the reaction, resulting in only a minor reduction in reaction yields (**3n**–**3p**). Moreover, aliphatic substrates had no significant effect on the yield of products, delivering the corresponding product at a 73% yield (**3m**). However, the direct carbonylation of the alkyl iodide compounds was unsuccessful (See more details in the Supplementary materials).

**Scheme 3:**
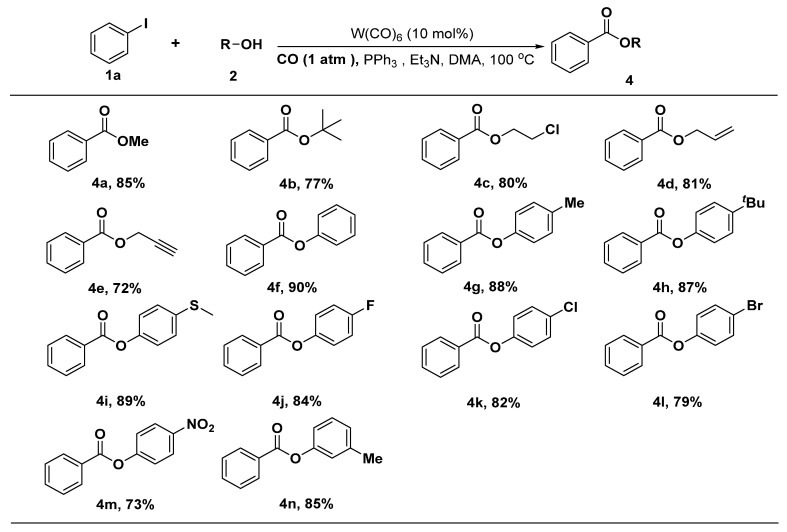
Substrate Scope of Carbonylative Synthesis of Benzoates. iodobenzene **1a** (0.5 mmol), alcohols or phenols **2** (0.75 mmol), CO balloon, W(CO)_6_ (10 mol%), Et_3_N (2 equiv.), PPh_3_ (15 mol%), DMA (2 mL), 100 °C, 24 h.

Subsequently, examination of the scope of the reaction (Scheme 3) was conducted with emphasis was placed on the alcohol part. Based on the aforementioned experimental findings, we concluded that alcohols with electron-rich characteristics were more proficient in this process. Pleasingly, alcohols like methanol could conveniently turn into methyl benzoate (**4a**) with an 85% yield. Noticeably, the sterically hindered *tert*-butanol was compatible, generating **4b** in yields of 77%. This catalytic system could also accommodate alcohols containing C=C or C≡C bonds, which gave **4d**–**4e** in 72–81% yields. The catalytic system served exceptionally well for the phenoxycarbonylation of iodobenzene in conjunction with phenols, demonstrating remarkable activity. Consequently, different phenols were tested without any additional optimization of the reaction parameters. An array of functional groups including alkyl groups (**4g**–**4h**), a methylthio substituent (**4i**), halogens (**4j**–**4l**), and a nitro moiety (**4m**) proved to be suitable coupling partners of this novel conversion. The methyl in the *ortho* positions of the phenyl ring had no obvious influence, providing the desired *m*-tolyl benzoate indoles successfully (**4n**).

**Scheme 4:**
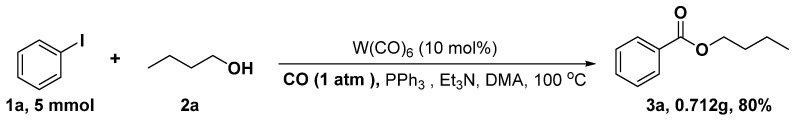
Large-Scale Preparation.

To thoroughly inspect the practicality of this reaction in synthesis, the gram-scale reaction was conducted under optimal conditions (Scheme 4). The production of **3a** on a gram scale was achieved by enhancing the catalyst dosage and extending the reaction duration, resulting in an outstanding yield. This specific result confirmed the potential application of this protocol in the facile synthesis of ester compounds.

**Scheme 5:**
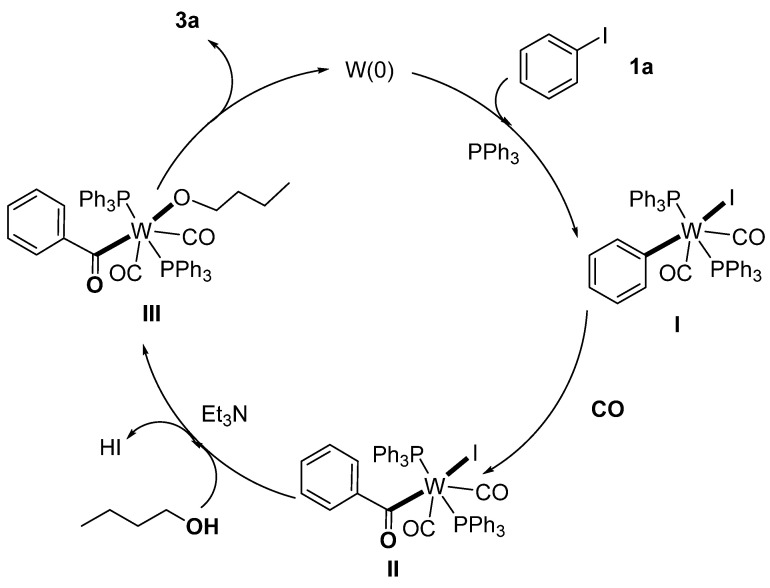
Plausible Mechanistic Pathway.

According to the literature precedents and our own preliminary investigation [8,22,27], the possible pathway for this conversion was outlined in Scheme 5. First, phenyl tungsten intermediate **I** was generated by the oxidative addition of aryl halide to the tungsten complex. Next, the carbonyl tungsten intermediate **II** was formed after the insertion of CO into the phenyl-tungsten bond. Subsequently, following the interaction with *n*-butanol (**2a**), intermediate **II** transformed into the tungsten intermediate **III**, accompanied by the release of HI. Finally, after the reductive elimination process, the desired product **3a** was delivered with a high yield.

## 3. Conclusions

In summary, a novel, universal, and effective method for the production of benzoates has been disclosed via the low-valent tungsten catalyzed tungsten-catalyzed carbonylative reaction of aryl iodides and alcohols or phenols wtih the atmospheric CO as the CO surrogates. This W(CO)_6_ system is straightforward, readily available, and operates in situ. It exhibits broad functional group compatibility, encompassing substrates with broad substituents or heteroatoms. This work suggests that tungsten complexes can act as a novel class of non-noble catalysts for carbonylative reaction, offering an alternative to the known Pd, Cu, Ni, and Co catalysts, which may hold significance for future developments.

## 4. Materials and Methods

### 4.1. Materials

All the reagents were obtained from commercial sources and used directly without further purification unless otherwise noted. ^1^H NMR and ^13^C NMR spectra were recorded on a Bruker AVANCE III 400 MHz. ^1^H NMR and ^13^C NMR chemical shifts were determined relative to internal standard TMS at *δ* 0.0. Chemical shifts (*δ*) are reported in ppm and coupling constants (*J*) are in Hertz (Hz).

### 4.2. General Methods for the Preparation of Benzoates

A 25 mL dried Schlenk tube was added the mixture of iodobenzene **1a** (0.50 mmol), *n*-butanol **2a** (0.75 mmol), W(CO)_6_ (10 mol%), Et_3_N (2 equiv.), and PPh_3_ (15 mol%) in anhydrous DMA (2.0 mL). The gas in the Schlenk tube was replaced by CO three times. The reaction was then stirred at 100 °C for 24 h. Upon completion, the reaction mixture was washed with a saturated NaCl aqueous solution (2 × 10 mL) and then extracted with ethyl acetate (2 × 10 mL), and the organic layers were combined, dried over anhydrous MgSO_4_, filtered, and concentrated under reduced pressure. The residue was separated by column chromatography (petroleum ether/ethyl acetate) to give the pure butyl benzoate **3a**.

## Figures and Tables

**Table 1:** Optimization of the reaction conditions ^a^.

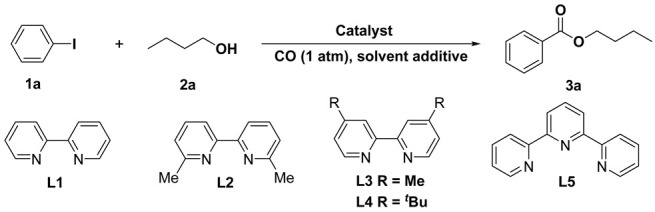				
Entry	Catalyst	Ligand	Solvent	Yield (%) ^b^
1	W(CO)_6_	PPh_3_	Toluene	69
2	W(CO)_3_(CH_3_CN)_3_	PPh_3_	Toluene	51
3	W(COD)_2_(CO)_4_	PPh_3_	Toluene	43
4	Mo(CO)_6_	PPh_3_	Toluene	34
5	W(CO)_6_	**L1**	Toluene	49
6	W(CO)_6_	**L2**	Toluene	53
**7**	W(CO)_6_	**L3**	Toluene	42
8 ^c^	W(CO)_6_	**L4**	Toluene	56
9	W(CO)_6_	**L5**	Toluene	38
10	W(CO)_6_	PPh_3_	THF	62
11	W(CO)_6_	PPh_3_	MeCN	61
12	W(CO)_6_	PPh_3_	DMSO	31
13	W(CO)_6_	PPh_3_	DMF	26
14	W(CO)_6_	PPh_3_	DMA	80
15	W(CO)_6_	PPh_3_	DCM	51
16	W(CO)_6_	PPh_3_	DCE	50
17	W(CO)_6_	PPh_3_	1,4-dioxane	54
18	W(CO)_6_	-	DMA	30
19	-	PPh_3_	DMA	trace
20 ^c^	W(CO)_6_	PPh_3_	THF	93
21 ^d^	W(CO)_6_	PPh_3_	THF	92

^a^ iodobenzene **1a** (0.5 mmol), *n*-butanol **2a** (0.75 mmol), ligand (15 mol%), catalyst (10 mol%), solvent (2 mL), Et_3_N (2 equiv.), 80 °C, 24 h, THF (tetrahydrofuran), DMSO (dimethyl sulfoxide), DCM (dichloromethane), DCE (1,2-dichloroethane); ^b^ Yields were determined by GC-MS; ^c^ the reaction temperature was 100 °C; ^d^ the reaction temperature was 120 °C.

## Data Availability

Data are contained within the article and Supplementary Materials.

## Supplementary Materials

The following supporting information can be downloaded at: https://www.mdpi.com/article/10.3390/molecules29225305/s1, the NMR data and spectra of the catalytic products. Refs. [28,29,30,31,32,33,34,35,36,37,38] are cited in the Supplementary Materials.
