# Supplementary material for: Low-Valent Tungsten Catalyzed Carbonylative Synthesis of Benzoates from Aryl Iodides and Alcohols
Source: Molecules. 2024 Nov 10;29(22):5305. doi: 10.3390/molecules29225305 (PMC11596934; doi:10.3390/molecules29225305)
Supplement: Supplementary file 1 [file molecules-29-05305-s001.zip › molecules-3274310-supplementary.pdf]

Supporting Information

for

Low-valent Tungsten Catalyzed Carbonylative Synthesis of Benzoates from Aryl Iodides and Alcohols

Feihua Ye, Lu Lin, Zhaoyang Huang, Yunwei Huang, Lixuan Huang, Xiang Li*, Chunsheng Li*

Table of Contents

General information S3

Characterization data for all products. S6

References. S12

NMR spectra for all the compounds S13

General information

Melting points were measured using a melting point instrument and are uncorrected. ^{1}H and ^{13}C NMR spectra were recorded on a 400 MHz NMR spectrometer. The chemical shifts are referenced to signals at TMS (0 ppm) and 77.0 ppm, respectively, and chloroform was used as a solvent with TMS as the internal standard. GC-MS data were obtained using electron ionization. TLC was performed using commercially available 100-400 mesh silica gel plates (GF254). Unless otherwise noted, purchased chemicals were used without further purification.

Characterization data for all products

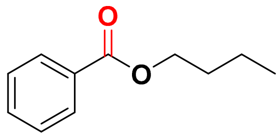

Butyl benzoate (**3a**) [1] was obtained after purification by column chromatography on silica gel (pe0troleum ether/ethyl acetate) as a light yellow oil (82.8 mg, 93%); ^{1}H NMR (400 MHz, Chloroform-d) δ 8.11 (d, J = 8.0 Hz, 2H), 7.62 (t, J = 8.0 Hz, 1H), 7.50 (t, J = 7.6 Hz, 2H), 4.39 (t, J = 6.6 Hz, 2H), 1.82 (dt, J = 14.4, 6.8 Hz, 2H), 1.56 (dt, J = 14.4, 7.2 Hz, 2H), 1.05 (t, J = 8.0 Hz, 3H); ^{13}C NMR (100 MHz, Chloroform-d) δ 166.7, 132.7, 130.6, 129.5, 128.3, 64.8, 30.8, 19.3, 13.7.

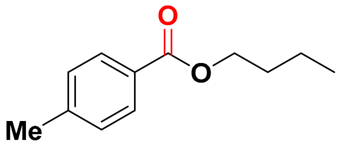

Butyl 4-methylbenzoate (**3b**) [1] was obtained after purification by column chromatography on silica gel (petroleum ether/ethyl acetate) as a light yellow oil (87.4 mg, 91%); ^{1}H NMR (400 MHz, Chloroform-d) δ 8.00 (d, J = 8.0 Hz, 2H), 7.31 (t, J = 7.8 Hz, 2H), 4.37 (t, J = 8.0 Hz, 2H), 2.47 (s, 3H), 1.81 (dt, J = 14.4, 6.8 Hz, 2H), 1.55 (dt, J = 14.4, 7.2 Hz, 2H), 1.04 (t, J = 7.4 Hz, 3H); ^{13}C NMR (100 MHz, Chloroform-d) δ 166.8, 143.4, 129.5, 129.0, 127.9, 102.0, 64.6, 30.8, 21.6, 19.3, 13.7.

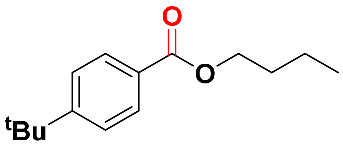

Butyl 4-(tert-butyl)benzoate (**3c**) [2] was obtained after purification by column chromatography on silica gel (petroleum ether/ethyl acetate) as a colorless oil (105.4 mg, 90%); ^{1}H NMR (400 MHz, Chloroform-d) δ 8.04 (d, J = 8.4 Hz, 2H), 7.52 (d, J = 8.4 Hz, 2H), 4.38 (t, J = 6.8 Hz, 2H), 1.81 (dt, J = 14.4, 6.6 Hz, 2H), 1.54 (dt, J = 14.4, 7.2 Hz, 2H), 1.40 (s, 9H), 1.04 (t, J = 8.0 Hz, 3H); ^{13}C NMR (100 MHz, Chloroform-d) δ 166.7, 156.4, 129.4, 127.8, 125.2, 64.6, 35.0, 31.1, 30.8, 19.3, 13.7.

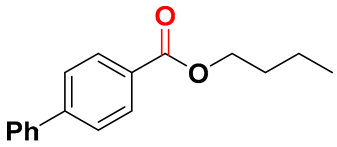

Butyl [1,1'-biphenyl]-4-carboxylate (**3d**) [2] was obtained after purification by column chromatography on silica gel (petroleum ether/ethyl acetate) as a light yellow oil (110.5 mg, 87 %); ^{1}H NMR (400 MHz, Chloroform-d) δ 8.19 (d, J = 8.0 Hz, 2H), 7.44 - 7.69 (m, 4H), 7.54 (t, J = 8.0 Hz, 2H), 7.47 (t, J = 7.2 Hz, 2H), 4.43 (t, J = 6.6 Hz, 2H), 1.85 (dt, J = 14.4, 6.6 Hz, 2H), 1.58 (dt, J = 14.4, 7.2 Hz, 2H), 1.07 (t, J = 8.0 Hz, 3H); ^{13}C NMR (100 MHz, Chloroform-d) δ 166.6, 145.6, 140.1, 130.1, 129.3, 128.9, 128.1, 127.3, 127.0, 64.9, 30.8, 19.3, 13.8.

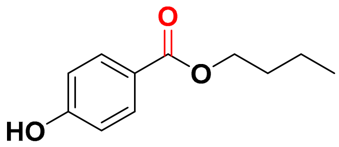

Butyl 4-hydroxybenzoate (**3e**) [3] was obtained after purification by column chromatography on silica gel (petroleum ether/ethyl acetate) as a solid (m. p. = 68 - 69 ºC) (79.6 mg, 82 %); ^{1}H NMR (400 MHz, Chloroform-d) δ 7.95 (d, J = 8.4 Hz, 2H), 6.90 (d, J = 8.4 Hz, 2H), 4.31 (t, J = 6.4 Hz, 2H), 1.74 (dt, J = 14.4, 6.6 Hz, 2H), 1.47 (dt, J = 14.4, 7.2 Hz, 2H), 0.97 (t, J = 8.0 Hz, 3H); ^{13}C NMR (100 MHz, Chloroform-d) δ 167.3, 160.5, 131.9, 122.4, 115.3, 64.9, 30.7, 19.2, 13.7.

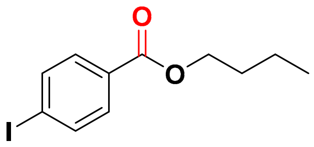

Butyl 4-iodobenzoate (**3f**) [4] was obtained after purification by column chromatography on silica gel (petroleum ether/ethyl acetate) as a light yellow oil (89.7 mg, 59 %); ^{1}H NMR (400 MHz, Chloroform-d) δ 7.86 (d, J = 8.4 Hz, 2H), 7.80 (d, J = 8.4 Hz, 2H), 4.37 (t, J = 6.8 Hz, 2H), 1.80 (dt, J = 14.6, 6.6 Hz, 2H), 1.54 (dt, J = 15.0, 7.4 Hz, 2H), 1.04 (t, J = 8.0 Hz, 3H); ^{13}C NMR (100 MHz, Chloroform-d) δ 166.1, 137.7, 131.0, 130.0, 100.5, 65.1, 30.7, 19.2, 13.7.

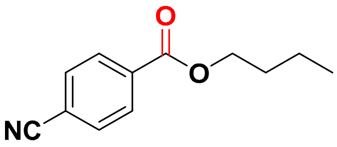

Butyl 4-cyanobenzoate (**3g**) [1] was obtained after purification by column chromatography on silica gel (petroleum ether/ethyl acetate) as a colorless solid (m. p. = 53 - 54 ºC) (80.2 mg, 79 %); ^{1}H NMR (400 MHz, Chloroform-d) δ 8.19 (d, J = 8.4 Hz, 2H), 7.80 (d, J = 8.4 Hz, 2H), 4.41 (t, J = 6.6 Hz, 2H), 1.82 (dt, J = 14.6, 6.7 Hz, 2H), 1.54 (dt, J = 15.0, 7.4 Hz, 2H), 1.04 (t, J = 7.4 Hz, 3H); ^{13}C NMR (100 MHz, Chloroform-d) δ 164.9, 132.1, 130.0, 117.9, 116.3, 65.6, 30.6, 19.2, 13.6.

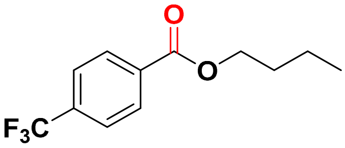

Butyl 4-(trifluoromethyl)benzoate (**3h**) [1] was obtained after purification by column chromatography on silica gel (petroleum ether/ethyl acetate) as a yellow oil (100.9 mg, 82 %); ^{1}H NMR (400 MHz, Chloroform-d) δ 8.16 (d, J = 8.0 Hz, 2H), 7.70 (d, J = 8.0 Hz, 2H), 4.37 (t, J = 6.6 Hz, 2H), 1.77 (dt, J = 14.4, 6.8 Hz, 1H), 1.49 (dt, J = 15.0, 7.4 Hz, 2H), 0.99 (t, J = 8.0 Hz, 3H); ^{13}C NMR (100 MHz, Chloroform-d) δ 165.4, 134.3 (q, J = 32.6 Hz), 133.8, 129.9, 125.3 (q, J = 3.7 Hz), 123.7 (q, J = 271.0 Hz), 65.4, 30.7, 19.2, 13.7.

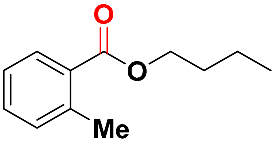

Butyl 2-methylbenzoate (**3i**) [1] was obtained after purification by column chromatography on silica gel (petroleum ether/ethyl acetate) as a colorless oil (85.5 mg, 89 %); ^{1}H NMR (400 MHz, Chloroform-d) δ 7.98 (d, J = 8.0 Hz, 1H), 7.45 (t, J = 8.0 Hz, 1H), 7.32 - 7.29 (m, 2H), 4.37 (t, J = 6.8 Hz, 2H), 2.67 (s,

3H), 1.82 (dt, J = 14.6, 6.8 Hz, 2H), 1.56 (dt, J = 15.0, 7.2 Hz, 2H), 1.05 (t, J = 8.0 Hz, 3H); ^{13}C NMR (100 MHz, Chloroform-d) δ 167.8, 140.0, 131.7, 131.6, 130.5, 130.1, 125.6, 64.6, 30.8, 21.7, 19.3, 13.7.

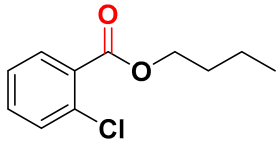

Butyl 2-chlorobenzoate (**3j**) [1] was obtained after purification by column chromatography on silica gel (petroleum ether/ethyl acetate) as a yellow oil (89.1 mg, 84 %); ^{1}H NMR (400 MHz, Chloroform-d) δ 8.07 (d, J = 8.0 Hz, 1H), 8.00 - 7.98 (m, 1H), 7.60 - 7.57 (m, 1H), 7.44 (t, J = 8.0 Hz, 1H), 4.39 (t, J = 6.8 Hz, 2H), 1.82 (dt, J = 14.4, 6.8 Hz, 2H), 1.56 (dt, J = 15.0, 7.2 Hz, 2H), 1.04 (t, J = 8.0 Hz, 3H); ^{13}C NMR (100 MHz, Chloroform-d) δ 134.5, 132.8, 132.3, 129.6, 127.7, 102.0, 65.2, 30.7, 19.2, 13.7.

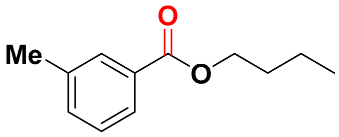

Butyl 3-methylbenzoate (**3k**) [1] was obtained after purification by column chromatography on silica gel (petroleum ether/ethyl acetate) as a colorless oil (84.5 mg, 88 %); ^{1}H NMR (400 MHz, Chloroform-d) δ 7.91 (d, J = 8.8 Hz, 2H), 7.43 - 7.37 (m, 2H), 4.38 (t, J = 6.6 Hz, 3H), 2.47 (s, 3H), 1.82 (dt, J = 14.6, 6.7 Hz, 2H), 1.56 (dt, J = 15.0, 7.4 Hz, 2H), 1.05 (t, J = 8.0 Hz, 3H); ^{13}C NMR (100 MHz, Chloroform-d) δ 166.9, 138.1, 133.5, 130.5, 130.0, 128.2, 126.7, 64.7, 30.8, 21.2, 19.3, 13.7.

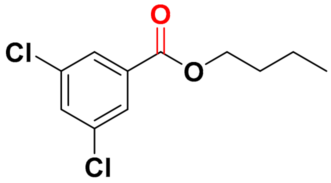

Butyl 3,5-dichlorobenzoate (**3l**) [5] was obtained after purification by column chromatography on silica gel (petroleum ether/ethyl acetate) as a yellow oil (102.1 mg, 83 %); ^{1}H NMR (400 MHz, Chloroform-d) δ 7.95 (s, 2H), 7.59 (s, 1H), 4.39 (t, J = 6.6 Hz, 2H), 1.81 (dt, J = 14.4, 6.8 Hz, 2H), 1.54 (dt, J = 15.0, 7.4 Hz, 2H), 1.04 (t, J = 8.0 Hz, 2H); ^{13}C NMR (100 MHz, Chloroform-d) δ 164.3, 135.2, 133.4, 132.6, 128.0, 65.7, 30.6, 19.2, 13.7.

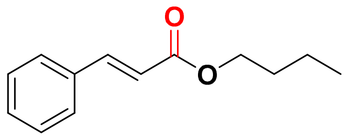

Butyl cinnamate (**3m**) [3] was obtained after purification by column chromatography on silica gel (petroleum ether/ethyl acetate) as a yellow solid (m. p. = 139 - 140 ºC) (74.5 mg, 73 %); ^{1}H NMR (400 MHz, Chloroform-d) δ 7.75 (d, J = 16.0 Hz, 1H), 7.60 - 7.58 (m, 4H), 7.45 - 7.44 (m, 3H), 6.51 (d, J = 16.0 Hz, 1H), 4.28 (t, J = 6.8 Hz, 2H), 1.76 (dt, J = 14.4, 6.8 Hz, 2H), 1.52 (dt, J = 15.0, 7.4 Hz, 2H), 1.03 (t, J = 8.0 Hz, 3H); ^{13}C NMR (100 MHz, Chloroform-d) δ 167.0, 144.5, 134.5, 130.1, 128.8, 128.0, 118.3, 64.4, 30.8, 19.2, 13.7.

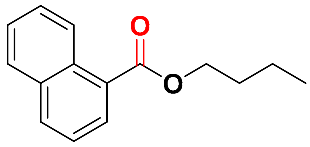

Butyl 1-naphthoate (**3n**) [2] was obtained after purification by column chromatography on silica gel (petroleum ether/ethyl acetate) as a yellow oil (95.8 mg, 84 %); ^{1}H NMR (400 MHz, Chloroform-d) δ 9.00 (d, J = 8.0 Hz, 1H), 8.27 - 8.25 (m, 1H), 8.08 (d, J = 8.0 Hz, 1H), 7.95 (d, J = 8.0 Hz, 1H), 7.71 - 7.67 (m, 1H), 7.62 - 7.55 (m, 2H), 4.51 (d, J = 6.6 Hz, 2H), 1.93 - 1.86 (m, 2H), 1.65 - 1.56 (m, 2H), 1.08 (t, J = 7.4 Hz, 3H); ^{13}C NMR (100 MHz, Chloroform-d) δ 167.7, 133.9, 133.1, 131.4, 130.0, 128.5, 127.6, 126.1, 125.8, 124.5, 64.9, 30.8, 19.4, 13.7.

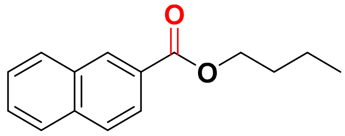

Butyl 2-naphthoate (**3o**) [5] was obtained after purification by column chromatography on silica gel (petroleum ether/ethyl acetate) as a yellow solid (m. p. = 41 - 42 ºC) (96.9 mg, 85 %); ^{1}H NMR (400 MHz, Chloroform-d) δ 8.68 (s, 1H), 8.15 - 8.13 (m, 1H), 8.03 (d, J = 8.0 Hz, 1H), 7.95 (d, J = 8.0 Hz, 2H), 7.67 - 7.59 (m, 2H), 4.46 (t, J = 6.6 Hz, 2H), 1.88 (dt, J = 14.6, 6.7 Hz, 2H), 1.59 (dt, J = 15.0, 7.4 Hz, 2H), 1.08 (d, J = 8.0 Hz, 2H); ^{13}C NMR (100MHz, Chloroform-d) δ 166.8, 135.5, 132.5, 130.9, 129.3, 128.1, 127.7, 126.5, 125.2, 65.0, 30.8, 19.3, 13.8.

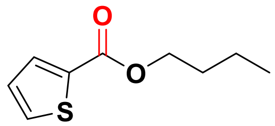

Butyl thiophene-2-carboxylate (**3p**) [5] was obtained after purification by column chromatography on silica gel (petroleum ether/ethyl acetate) as a yellow oil (80.1 mg, 87 %); ^{1}H NMR (400 MHz, Chloroform-d) δ 7.86 - 7.85 (m, 1H), 7.61 - 7.59 (m, 1H), 7.17 - 7.14 (m, 1H), 4.36 (t, J = 6.6 Hz, 2H), 1.83 - 1.76 (m, 2H), 1.57 - 1.49 (m, 2H), 1.03 (t, J = 7.4 Hz, 3H); ^{13}C NMR (100 MHz, Chloroform-d) δ 162.3, 134.2, 133.2, 132.1, 127.6, 65.0, 30.7, 19.2, 13.7.

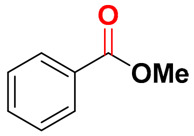

Methyl benzoate (**4a**) [1] was obtained after purification by column chromatography on silica gel (petroleum ether/ethyl acetate) as a white solid (M. p.=116-117 ºC) (57.8 mg, 85%); ^{1}H NMR (400 MHz, Chloroform-*d*) δ 8.11 (d, J = 8.0 Hz, 2H), 7.62 (t, J = 8.0 Hz, 1H), 7.50 (t, J = 8.0 Hz, 2H), 3.98 (s, 3H); ^{13}C NMR (100 MHz, Chloroform-*d*) δ 167.1, 132.8, 130.2, 129.5, 128.3, 52.0.

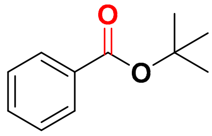

***tert*-Butyl benzoate** (**4b**) [6] was obtained after purification by column chromatography on silica gel (petroleum ether/ethyl acetate) as a white solid (M. p.=97-98 ºC) (68.6 mg, 77%); ^{1}H NMR (400 MHz, Chloroform-*d*) δ 8.06 (d, J = 8.0 Hz, 2H), 7.59 (t, J = 8.0 Hz, 1H), 7.48 (t, J = 8.0 Hz, 2H), 1.66 (s, 9H); ^{13}C NMR (100 MHz, Chloroform-*d*) δ 165.8, 132.4, 132.1, 129.4, 128.1, 28.2.

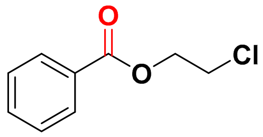

2-Chloroethyl benzoate (**4c**) [5] was obtained after purification by column chromatography on silica gel (petroleum ether/ethyl acetate) as a colorless oil (73.6 mg, 80%); ^{1}H NMR (400 MHz, Chloroform-*d*) δ

8.07 (d, J = 8.0 Hz, 2H), 7.58 (t, J = 8.0 Hz, 1H), 7.45 (t, J = 8.0 Hz, 2H), 4.57 (t, J = 6.0 Hz, 2H), 3.82 (t, J = 6.0 Hz, 2H); ^{13}C NMR (100 MHz, Chloroform-*d*) δ 166.1, 133.2, 129.7, 129.6, 128.4, 64.4, 41.6.

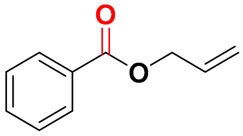

Allyl benzoate (**4d**) [7] was obtained after purification by column chromatography on silica gel (petroleum ether/ethyl acetate) as a white solid (M. p.=138-139 ºC) (65.6 mg, 81 %); ^{1}H NMR (400 MHz, Chloroform-*d*) δ 8.15 - 8.12 (m, 2H), 7.65 - 7.61 (m, 1H), 7.51 (t, J = 8.0 Hz, 2H), 6.16 - 6.06 (m, 1H), 5.51 - 5.46 (m, 1H), 5.37 - 5.34 (m, 1H), 4.91 - 4.89 (m, 2H); ^{13}C NMR (100 MHz, Chloroform-*d*) δ 166.2, 132.9, 132.3, 130.2, 129.6, 128.3, 118.2, 65.5.

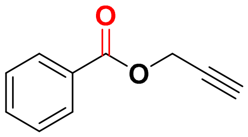

Prop-2-yn-1-yl benzoate (**4e**) [7] was obtained after purification by column chromatography on silica gel (petroleum ether/ethyl acetate) as a white solid (M. p.=120-121 ºC) (57.6 mg, 72 %); ^{1}H NMR (400 MHz, Chloroform-*d*) δ 8.14 (d, J = 8.0 Hz, 2H), 7.66 - 7.62 (m, 1H), 7.52 (t, J = 8.0 Hz, 2H), 4.99 (d, J = 2.5 Hz, 1H), 2.59 (t, J = 2.4 Hz, 2H); ^{13}C NMR (100 MHz, Chloroform-*d*) δ 165.8, 133.3, 129.8, 129.4, 128.4, 77.7, 75.0, 52.4.

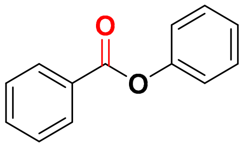

Phenyl benzoate (**4f**) [6] was obtained after purification by column chromatography on silica gel (petroleum ether/ethyl acetate) as a white solid (M. p.=124-125 ºC) (89.1mg, 90 %); ^{1}H NMR (400 MHz, Chloroform-*d*) δ 8.31 - 8.29 (m, 2H), 7.74 - 7.70 (m, 1H), 7.61 - 7.58 (m, 2H), 7.54 - 7.50 (m, 2H), 7.38 - 7.30 (m, 3H); ^{13}C NMR (100 MHz, Chloroform-*d*) δ 165.1, 151.0, 133.5, 130.1, 129.5, 128.5, 125.8, 121.7, 101.9.

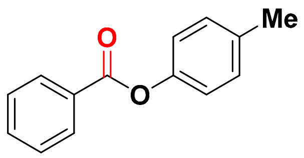

***p*-Tolyl benzoate** (**4g**) [8] was obtained after purification by column chromatography on silica gel (petroleum ether/ethyl acetate) as a white solid (M. p.=127-128 ºC) (93.3 mg, 88 %); ^{1}H NMR (400 MHz, Chloroform-*d*) *δ* 8.30 - 8.27 (m, 2H), 7.71 (t, *J* = 8.0 Hz, 1H), 7.59 (t, *J* = 7.6 Hz, 2H), 7.30 (d, *J* = 8.4 Hz, 2H), 7.18 (d, *J* = 8.4 Hz, 2H), 2.46 (s, 3H); ^{13}C NMR (100 MHz, Chloroform-*d*) *δ* 165.3, 148.8, 135.5, 133.4, 130.1, 130.0, 128.5, 121.3, 102.0, 20.9.

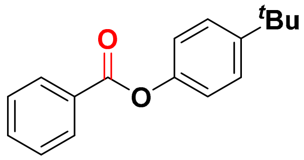

4-(*tert*-Butyl)phenyl benzoate (**4h**) [8] was obtained after purification by column chromatography on silica gel (petroleum ether/ethyl acetate) as a white solid (M. p.=107 - 108 ºC) (110.5 mg, 87 %); ^{1}H NMR (400 MHz, Chloroform-*d*) *δ* 8.30 - 8.28 (m, 2H), 7.71 (t, *J* = 8.0 Hz, 1H), 7.59 (t, *J* = 8.0 Hz, 1H), 7.53 - 7.51 (m, 1H), 7.23 - 7.21 (m, 2H), 1.42 (s, 9H); ^{13}C NMR (100 MHz, Chloroform-*d*) *δ* 165.3, 148.7, 133.4, 130.1, 129.8, 128.5, 126.3, 121.0, 102.0, 34.5, 31.4.

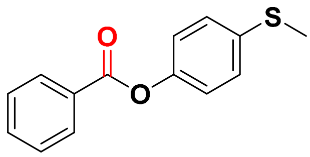

4-(Methylthio)phenyl benzoate (**4i**) [9] was obtained after purification by column chromatography on silica gel (petroleum ether/ethyl acetate) as a white solid (M.p.=94-96 ºC) (108.6 mg, 89 %); ^{1}H NMR (400 MHz, Chloroform-*d*) *δ* 8.27 (d, *J* = 8.0 Hz, 2H), 7.71 (t, *J* = 8.0 Hz, 1H), 7.58 (t, *J* = 8.0 Hz, 2H), 7.40 (d, *J* = 8.6 Hz, 2H), 7.23 (d, *J* = 8.6 Hz, 2H), 2.57 (s, 3H); ^{13}C NMR (100 MHz, Chloroform-*d*) *δ* 165.1, 148.7, 135.7, 133.6, 130.1, 129.5, 128.5, 128.1, 122.1, 16.5.

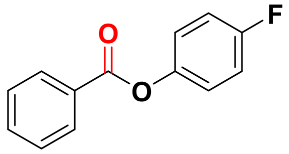

4-Fluorophenyl benzoate (**4j**) [10] was obtained after purification by column chromatography on silica gel (petroleum ether/ethyl acetate) as a yellow oil (90.7 mg, 84 %); ^{1}H NMR (400 MHz, Chloroform-*d*) δ

8.27 (d, J = 8.0 Hz, 2H), 7.72 (t, J = 8.0 Hz, 1H), 7.59 (t, J = 8.0 Hz, 2H), 7.27 - 7.24 (m, 2H), 7.21 - 7.16 (m, 2H); ^{13}C NMR (101 MHz, Chloroform-*d*) δ 165.2, 160.3 (d, J = 244.2 Hz), 146.8, 133.7, 130.2, 128.6, 123.1 (d, J = 8.1 Hz), 116.1 (d, J = 23.4 Hz), 102.0.

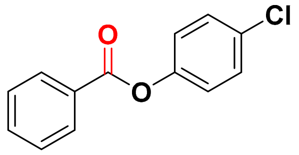

4-Chlorophenyl benzoate (**4k**) [8] was obtained after purification by column chromatography on silica gel (petroleum ether/ethyl acetate) as a white solid (M. p.=177 - 178 ºC) (95.1 mg, 82 %); ^{1}H NMR (400 MHz, Chloroform-*d*) δ 8.27 (d, J = 8.0 Hz, 2H), 7.72 (t, J = 8.0 Hz, 1H), 7.59 (t, J = 8.0 Hz, 2H), 7.48 - 7.45 (m, 2H), 7.25 - 7.23 (m, 2H); ^{13}C NMR (100 MHz, Chloroform-*d*) δ 164.9, 149.5, 133.8, 131.3, 130.2, 129.6, 128.6, 123.1, 102.0.

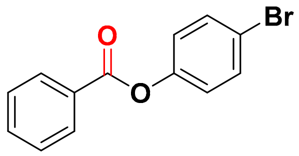

4-Bromophenyl benzoate (**4l**) [11] was obtained after purification by column chromatography on silica gel (petroleum ether/ethyl acetate) as a white solid (M. p.=115 - 116 ºC) (109.0 mg, 79 %); ^{1}H NMR (400 MHz, Chloroform-*d*) δ 8.26 (d, J = 8.0 Hz, 2H), 7.72 (t, J = 8.0 Hz, 1H), 7.63 - 7.57 (m, 4H), 7.20 - 7.18 (m, 2H); ^{13}C NMR (101 MHz, Chloroform-*d*) δ 164.8, 150.0, 133.8, 132.5, 130.2, 129.2, 128.6, 123.5, 118.9.

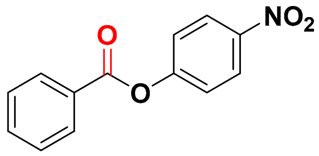

4-Nitrophenyl benzoate (**4m**) [11] was obtained after purification by column chromatography on silica gel (petroleum ether/ethyl acetate) as a white solid (M. p.=143 - 144 ºC) (88.7 mg, 73 %); ^{1}H NMR (400 MHz, Chloroform-*d*) δ 8.41 - 8.38 (m, 2H), 8.28 - 8.26 (m, 2H), 7.75 (t, J = 8.0 Hz, 1H), 7.61 (t, J = 8.0 Hz, 2H), 7.51 - 7.48 (m, 2H); ^{13}C NMR (100 MHz, Chloroform-*d*) δ 164.2, 155.7, 145.4, 134.2, 130.3, 128.8, 128.6, 125.2, 122.6.

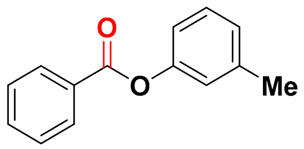

***m*-Tolyl benzoate** (**4n**) [8] was obtained after purification by column chromatography on silica gel (petroleum ether/ethyl acetate) as a white solid (M. p.=150 - 151 ºC) (90.1 mg, 85 %); ^{1}H NMR (400 MHz, Chloroform-*d*) δ 8.29 (d, J = 8.0 Hz, 2H), 7.71 (t, J = 8.0 Hz, 1H), 7.59 (t, J = 8.0 Hz, 2H), 7.39 (t, J = 8.0 Hz, 1H), 7.18 - 7.09 (m, 3H), 2.47 (s, 3H); ^{13}C NMR (100 MHz, Chloroform-*d*) δ 165.2, 151.0, 139.6, 133.5, 130.1, 129.7, 129.2, 128.5, 126.6, 122.3, 118.6, 21.3.

References

1. Kong, W.; Li, B.; Xu, X.; Song, Q. Fe-Catalyzed Aerobic Oxidative C–CN Bond Cleavage of Arylacetonitriles Leading to Various Esters. *J. Org. Chem.* **2016**, *81*, 8436–8441.
2. Nakatani, Y.; Koizumi, Y.; Yamasaki, Y.; Saito, S. Copper-Catalyzed Synthesis of Esters from Ketones. Alkyl Group as a Leaving Group. *Org. Lett.* **2008**, *10*, 2067-2071.
3. Iwasaki, T.; Maegawa, Y.; Hayashi, Y.; Ohshima, T.; Mashima, K. Transesterification of Various Methyl Esters Under Mild Conditions Catalyzed by Tetranuclear Zinc Cluster. *J. Org. Chem.* **2008**, *73*, 5147–5150.
4. Corma, A.; Villoria-del-Álamo, B.; Rojas-Buzo, S.; García-García, P. Zr-MOF-808 as Catalyst for Amide Esterification. *Chem. Eur. J.* **2021**, *27*, 4588–4598.
5. Yuan, Y.; Wu, X.-F. Synthesis of Esters from Stable and Convenient Sulfoxonium Precursors under Catalyst- and Additive-Free Conditions. *Synlett.* **2019**, *30*, 1820–1824.
6. Ibrahim, M. B.; Suleiman, R. K.; Fettouhi, M.; El Ali, B. A palladium–bisoxazoline supported catalyst for selective synthesis of aryl esters and aryl amides via carbonylative coupling reactions. *RSC Adv.* **2016**, *6*, 78826–78837.
7. Chen, Z.; Wen, Y.; Fu, Y.; Chen, H.; Ye, M.; Luo, G. Graphene Oxide: An Efficient Acid Catalyst for the Construction of Esters from Acids and Alcohols. *Synlett.* **2017**, *28*, 981–985.
8. Liu, J.; Chen, J.; Xia, C. A simple and efficient recyclable phosphine-free catalytic system for alkoxycarbonylation and carbonylative Sonogashira coupling reactions of aryl iodides. *J. Catal.* **2008**, *253*, 50–56.
9. Wu, X.-F.; Neumann, H.; Beller, M A General and Efficient Palladium-Catalyzed Alkoxycarbonylation of Phenols To Form Esters through In Situ Formed Aryl Nonaflates. *Chem. Eur. J.* **2012**, *18*, 3831–3834.
10. Chai, L.; Zhao, Y.-H.; Young, D. J.; Lu, X.; Li, H.- X. Ni(II)-Mediated Photochemical Oxidative Esterification of Aldehydes with Phenols. *Org. Lett.* **2022**, *24*, 6908–6913.
11. Liao, W.-J.; Lin, S.-Y.; Kuo, Y.-S.; Liang, C.-F. Site-Selective Acylation of Phenols Mediated by a Thioacid Surrogate through Sodium Thiosulfate Catalysis. *Org. Lett.* **2022**, *24*, 4207–4211.

NMR spectra for all the compounds

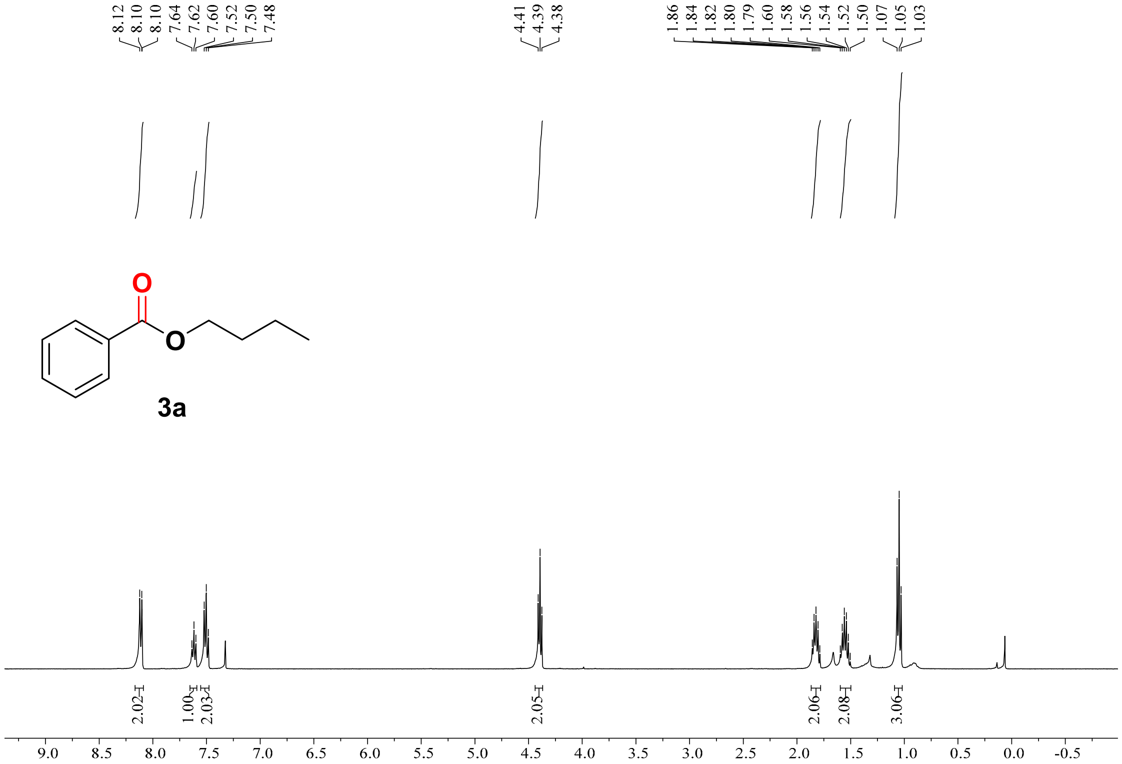

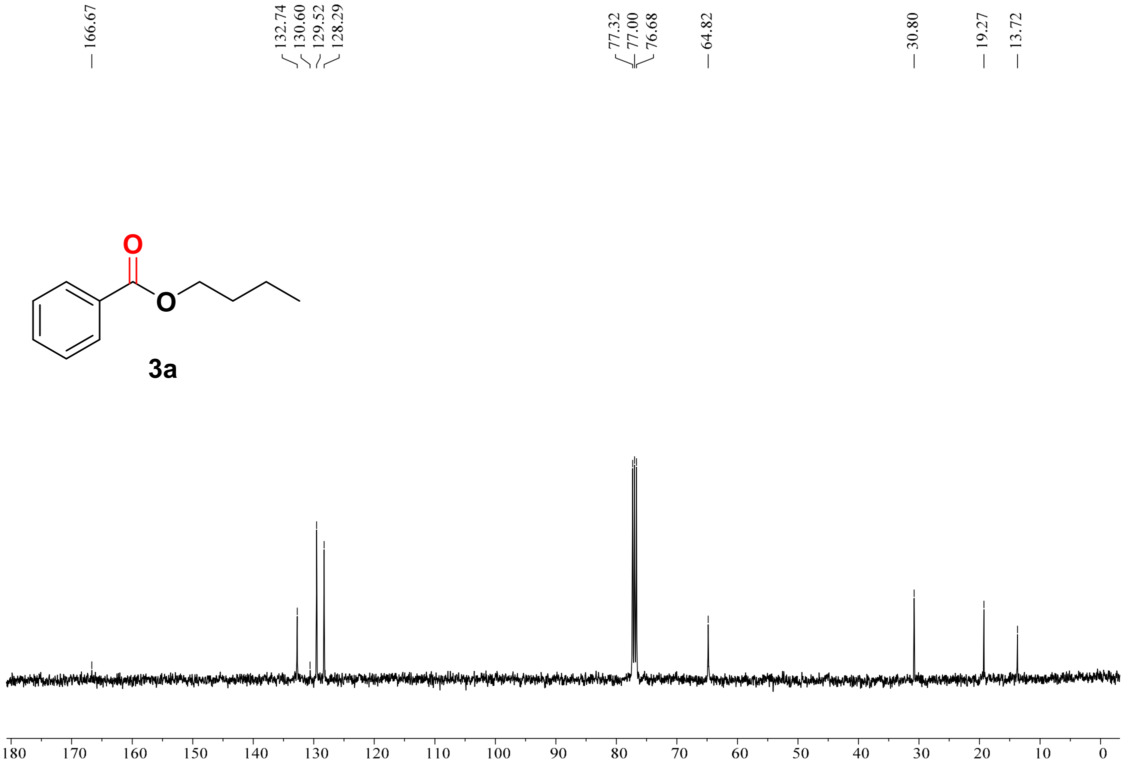

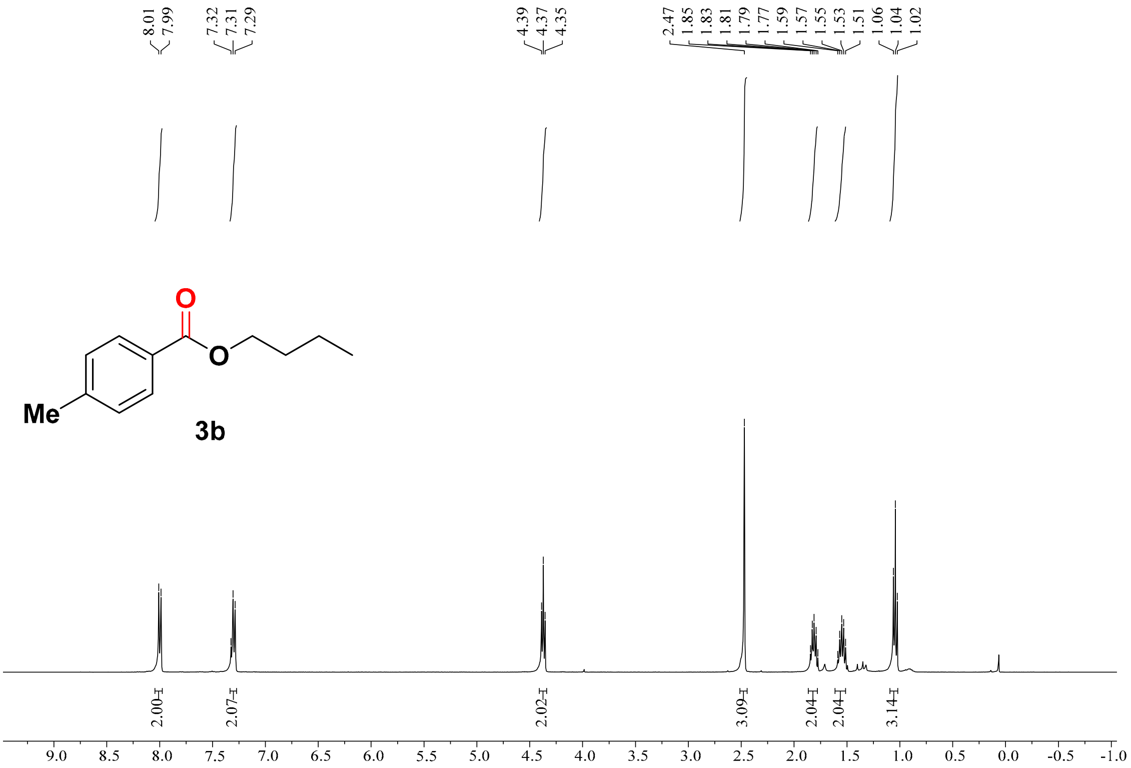
8.01
7.99
7.32
7.31
7.29
4.39
4.37
4.35
2.47
1.85
1.83
1.81
1.79
1.77
1.59
1.57
1.55
1.53
1.51
1.06
1.04
1.02
O
O
Me
2.00
2.07
2.02
3.09
2.04
2.04
3.14
9.0 8.5 8.0 7.5 7.0 6.5 6.0 5.5 5.0 4.5 4.0 3.5 3.0 2.5 2.0 1.5 1.0 0.5 0.0 -0.5 -1.0

3b

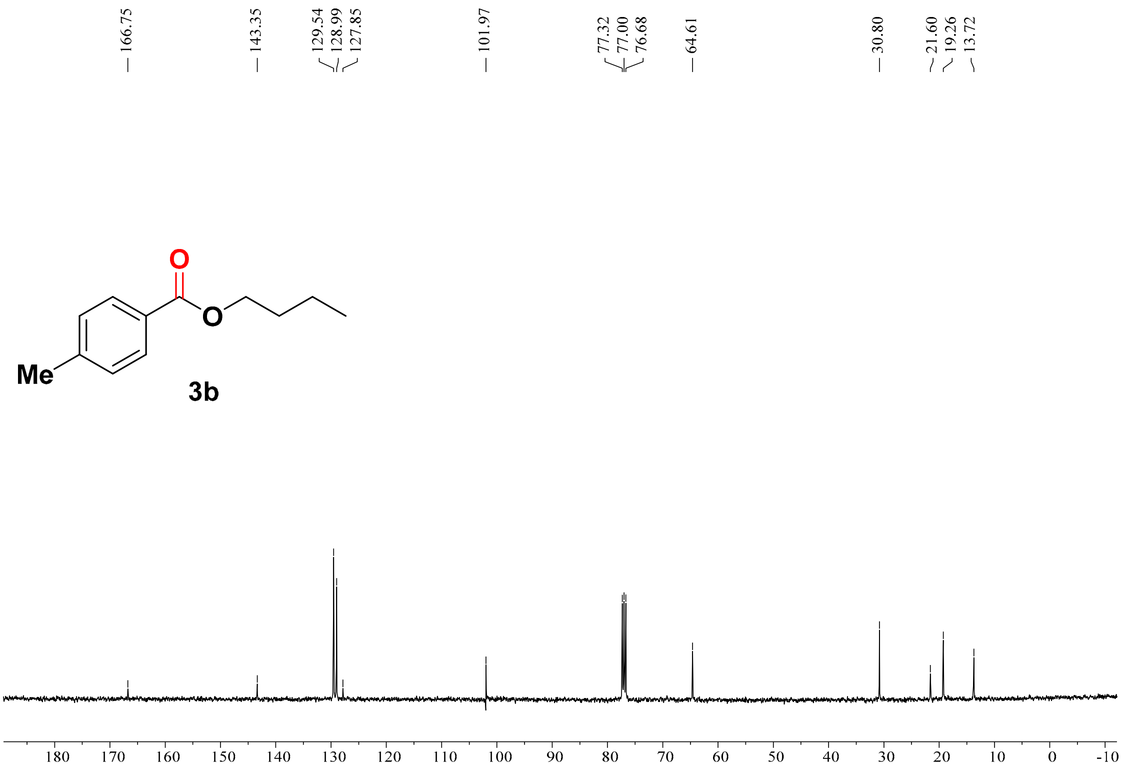
166.75
143.35
129.54
128.99
127.85
101.97
77.32
77.00
76.68
64.61
30.80
21.60
19.26
13.72
O
O
Me
180 170 160 150 140 130 120 110 100 90 80 70 60 50 40 30 20 10 0 -10

3b

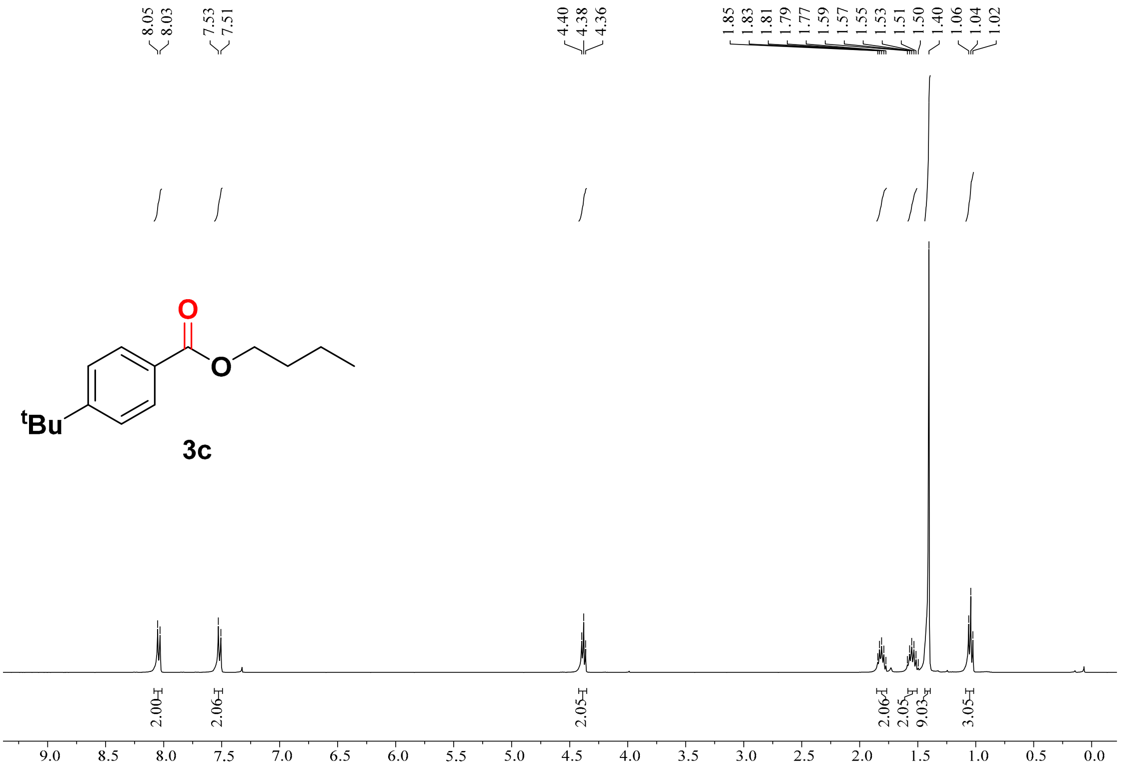

8.05
8.03
7.53
7.51
4.40
4.38
4.36
1.85
1.83
1.81
1.79
1.77
1.59
1.57
1.55
1.53
1.51
1.50
1.40
1.06
1.04
1.02
O
O
tBu
3c
2.00
2.06
2.05
2.06
2.05
9.03
3.05
9.0 8.5 8.0 7.5 7.0 6.5 6.0 5.5 5.0 4.5 4.0 3.5 3.0 2.5 2.0 1.5 1.0 0.5 0.0

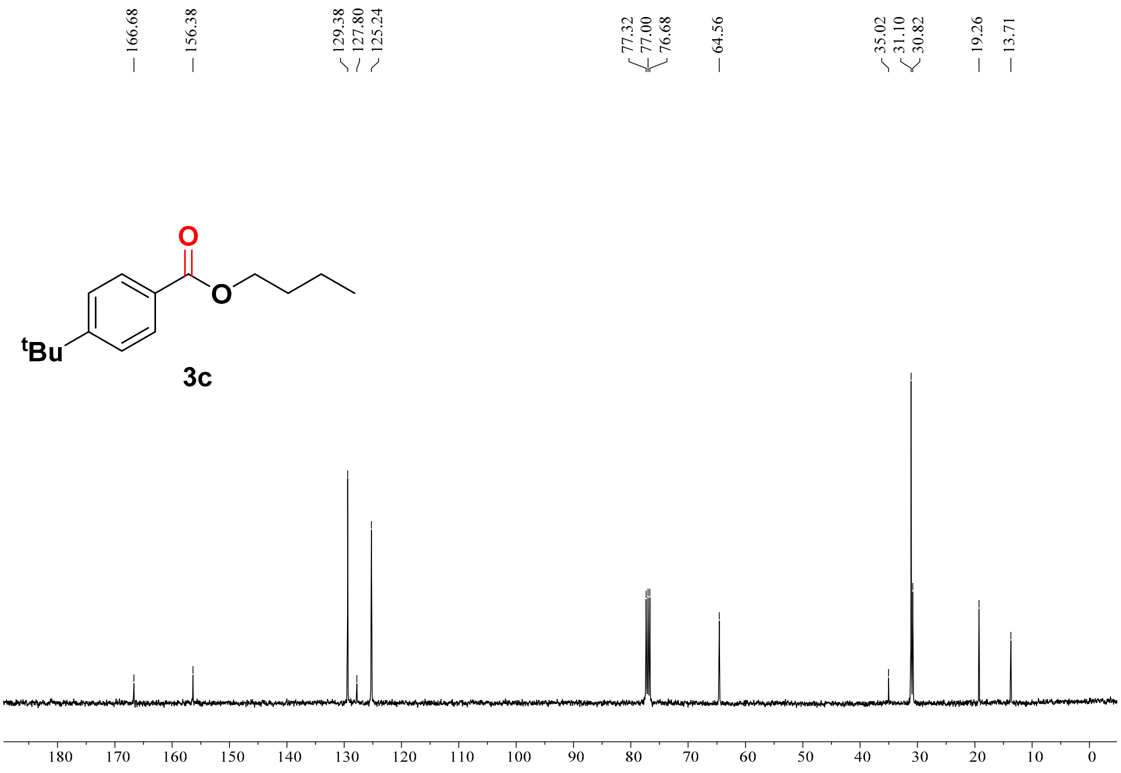

166.68
156.38
129.38
127.80
125.24
77.32
77.00
76.68
64.56
35.02
31.10
30.82
19.26
13.71
O
O
tBu
3c
180 170 160 150 140 130 120 110 100 90 80 70 60 50 40 30 20 10 0

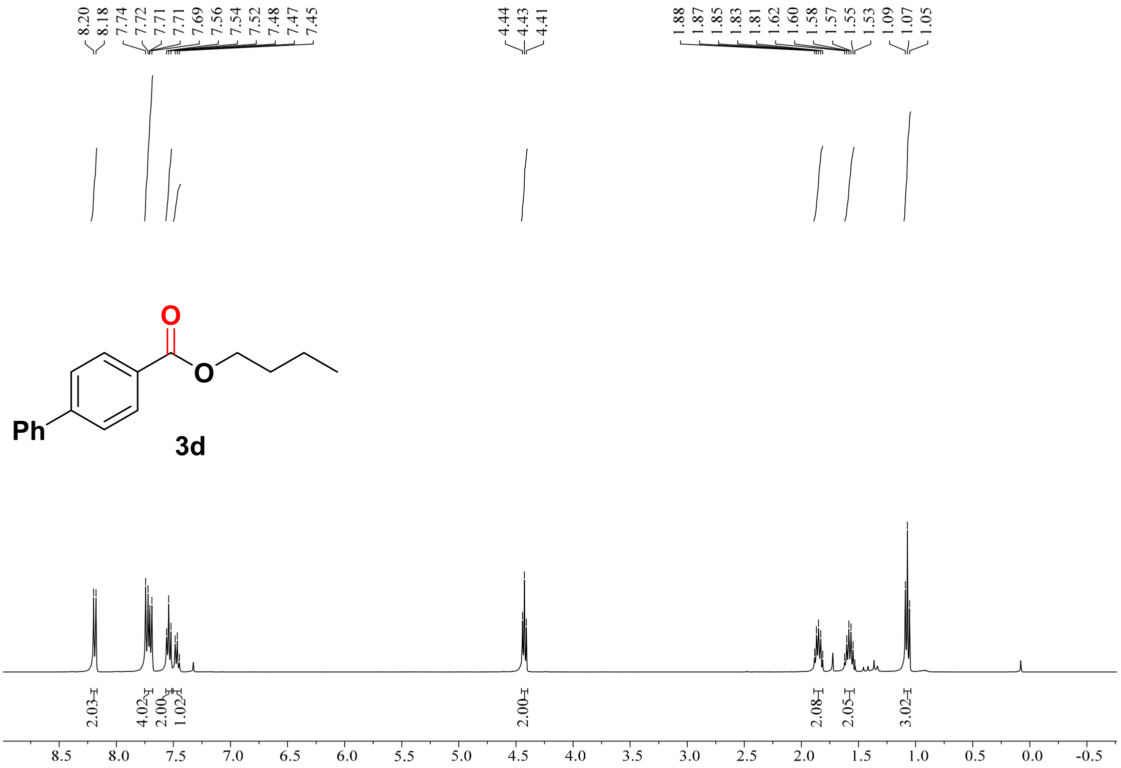
8.20
8.18
7.74
7.72
7.71
7.71
7.69
7.56
7.54
7.52
7.48
7.47
7.45
4.44
4.43
4.41
1.88
1.87
1.85
1.83
1.81
1.62
1.60
1.58
1.57
1.55
1.53
1.09
1.07
1.05
O
O
Ph
2.03
4.02
2.00
1.02
2.00
2.08
2.05
3.02
8.5 8.0 7.5 7.0 6.5 6.0 5.5 5.0 4.5 4.0 3.5 3.0 2.5 2.0 1.5 1.0 0.5 0.0 -0.5

3d

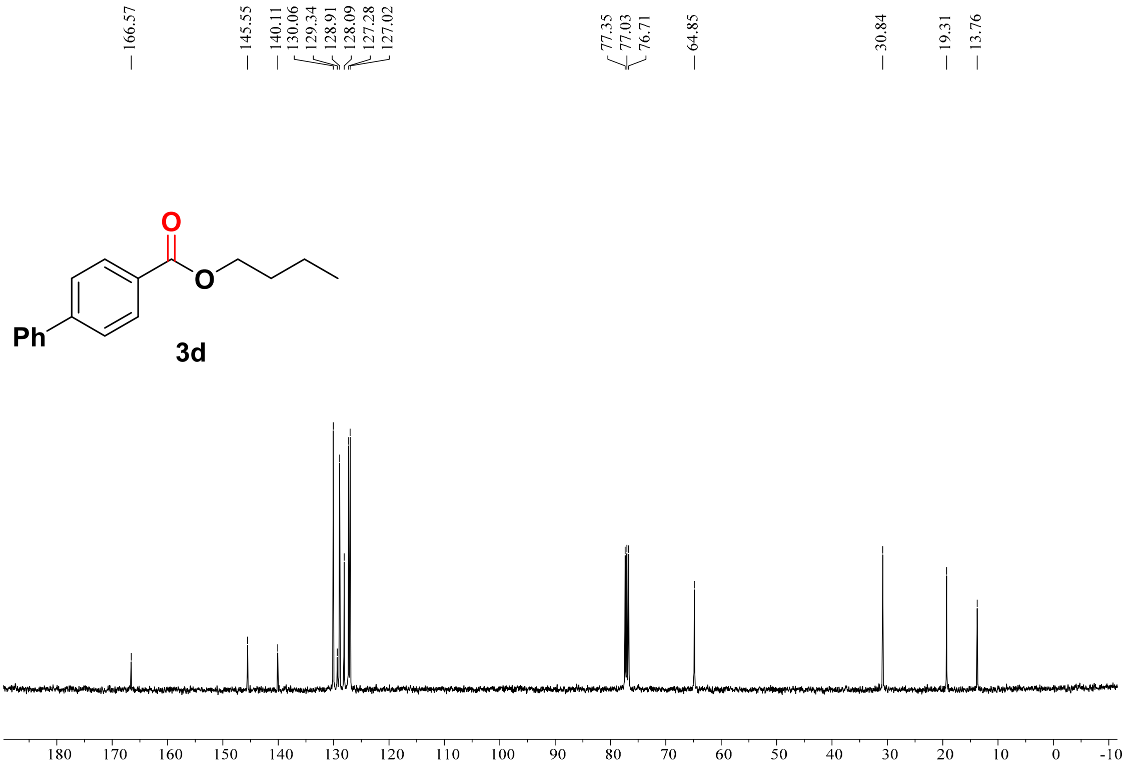
166.57
145.55
140.11
130.06
129.34
128.91
128.09
127.28
127.02
77.35
77.03
76.71
64.85
30.84
19.31
13.76
O
O
Ph
180 170 160 150 140 130 120 110 100 90 80 70 60 50 40 30 20 10 0 -10

3d

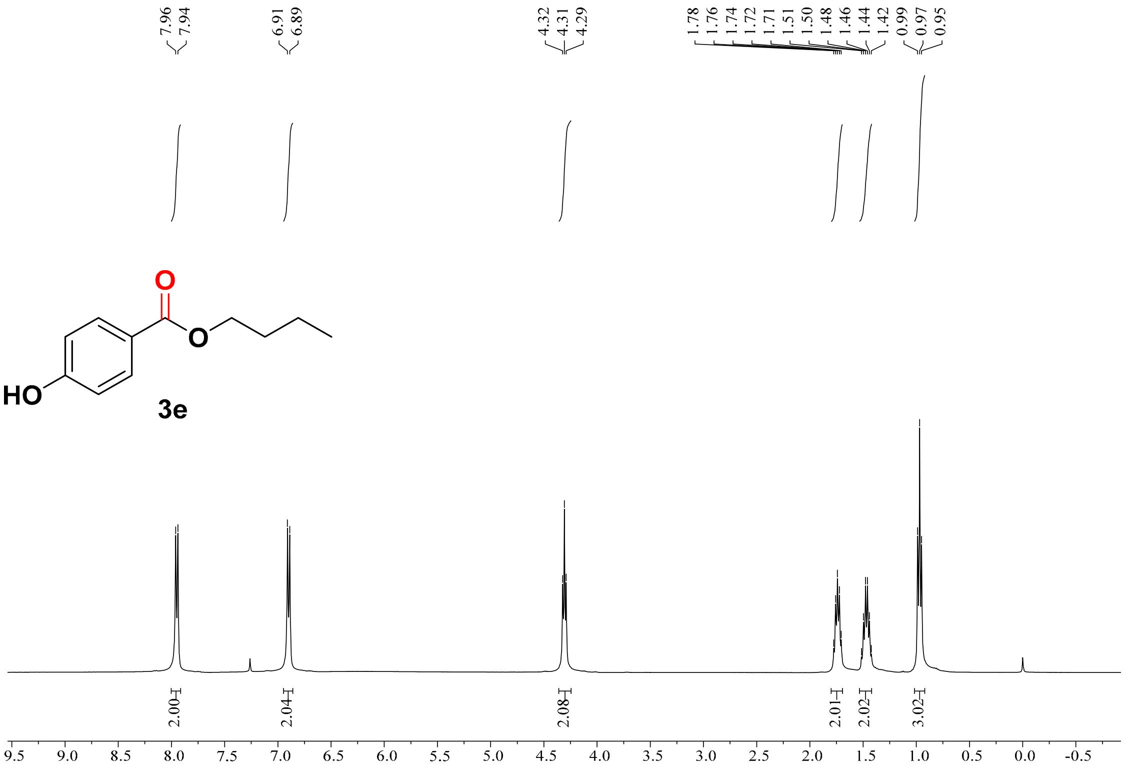
7.96
7.94
6.91
6.89
4.32
4.31
4.29
1.78
1.76
1.74
1.72
1.71
1.51
1.50
1.48
1.46
1.44
1.42
0.99
0.97
0.95
O
O
HO
3e
2.00
2.04
2.08
2.01
2.02
3.02
9.5 9.0 8.5 8.0 7.5 7.0 6.5 6.0 5.5 5.0 4.5 4.0 3.5 3.0 2.5 2.0 1.5 1.0 0.5 0.0 -0.5

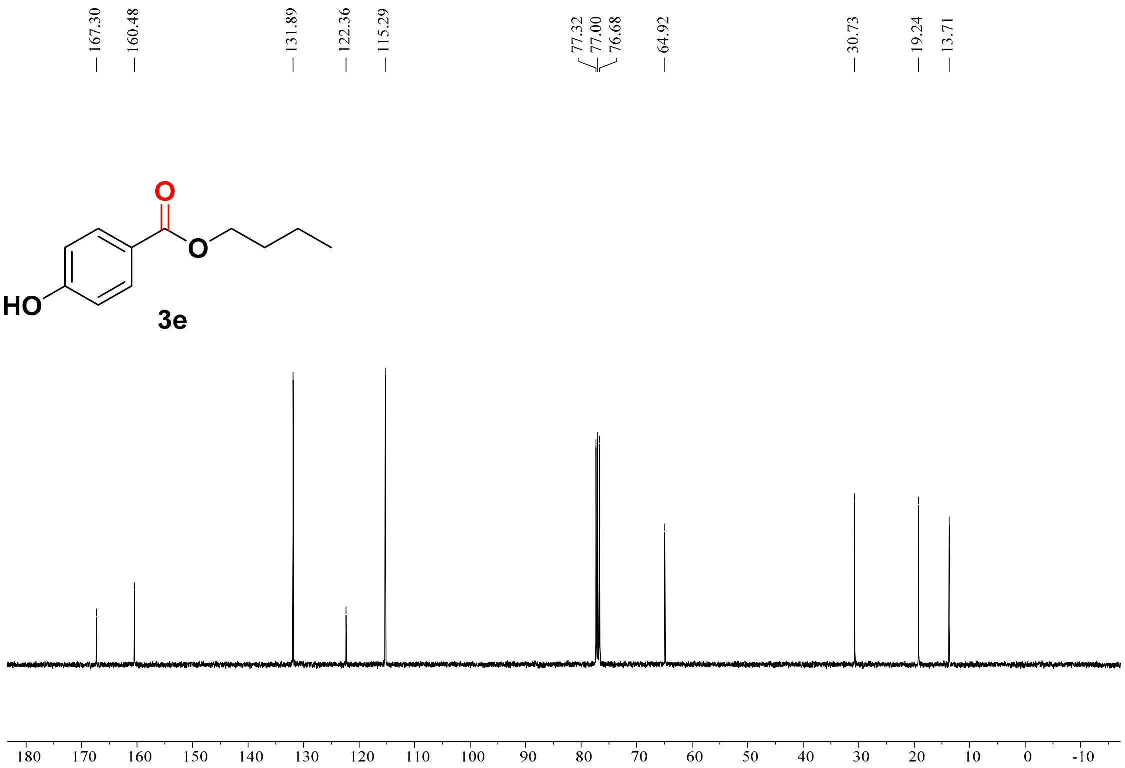
167.30
160.48
131.89
122.36
115.29
77.32
77.00
76.68
64.92
30.73
19.24
13.71
O
O
HO
3e
180 170 160 150 140 130 120 110 100 90 80 70 60 50 40 30 20 10 0 -10

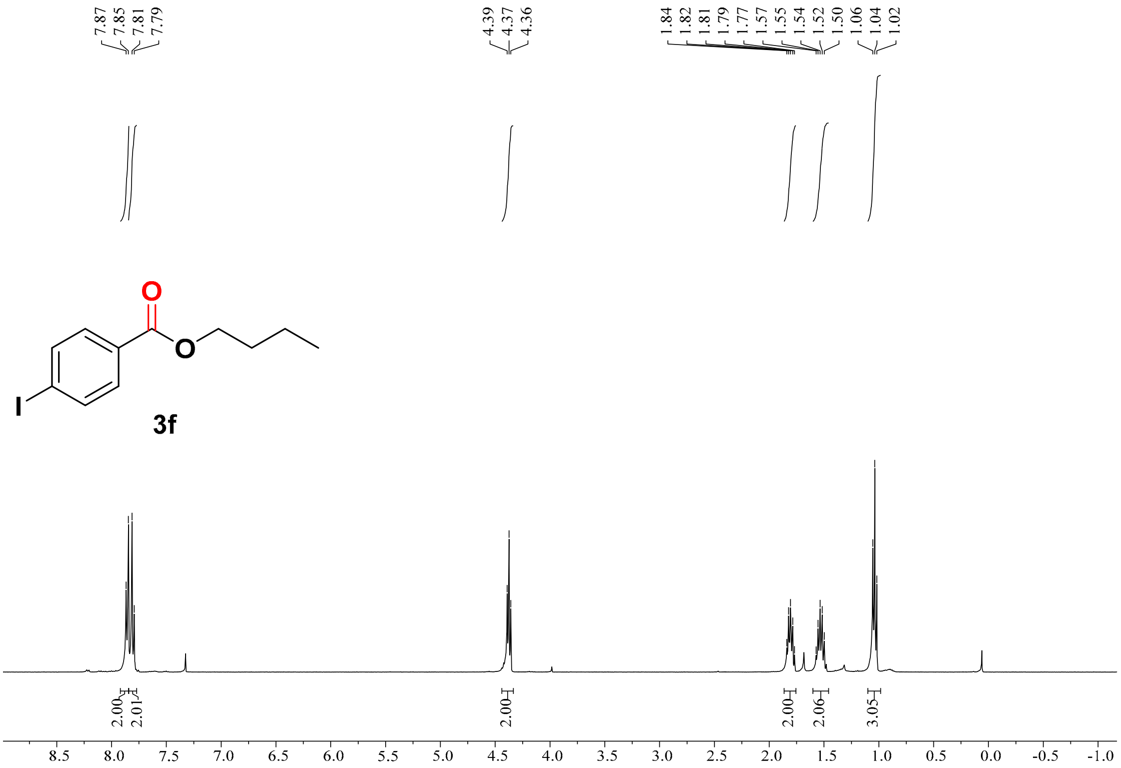
7.87
7.85
7.81
7.79
4.39
4.37
4.36
1.84
1.82
1.81
1.79
1.77
1.57
1.55
1.54
1.52
1.50
1.06
1.04
1.02
O
O
I
2.00
2.01
2.00
2.00
2.06
3.05
8.5
8.0
7.5
7.0
6.5
6.0
5.5
5.0
4.5
4.0
3.5
3.0
2.5
2.0
1.5
1.0
0.5
0.0
-0.5
-1.0

3f

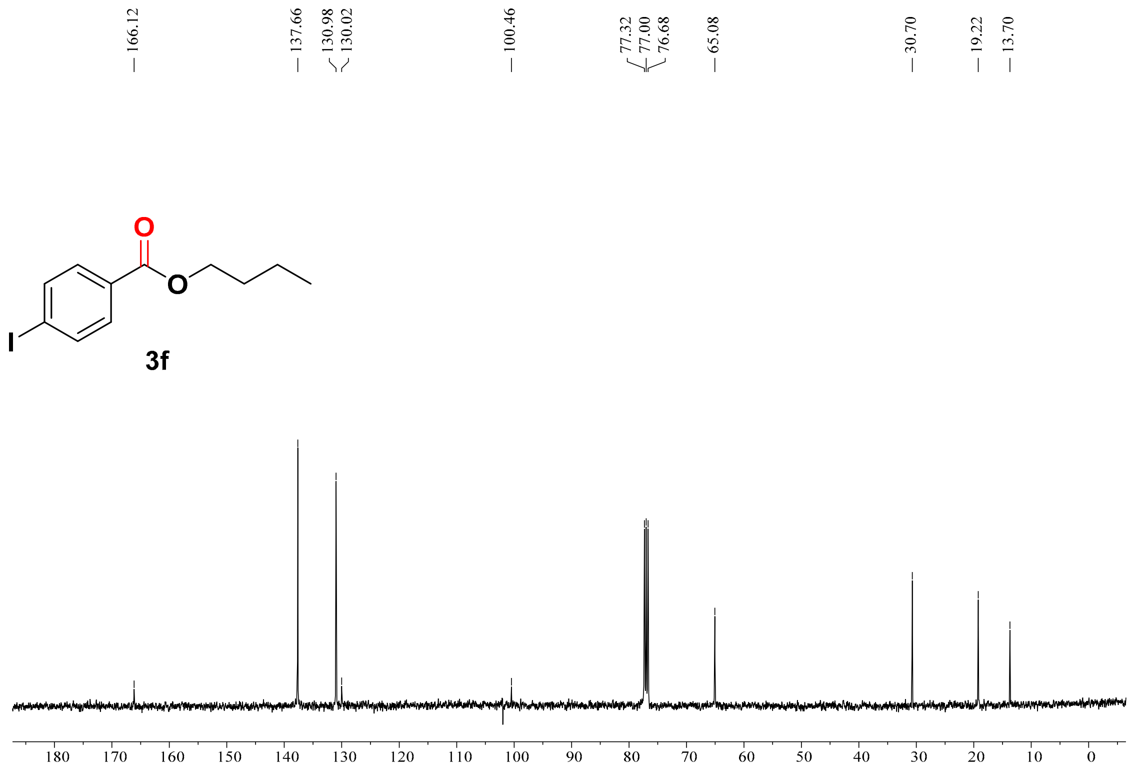
166.12
137.66
130.98
130.02
100.46
77.32
77.00
76.68
65.08
30.70
19.22
13.70
O
O
I
180
170
160
150
140
130
120
110
100
90
80
70
60
50
40
30
20
10
0

3f

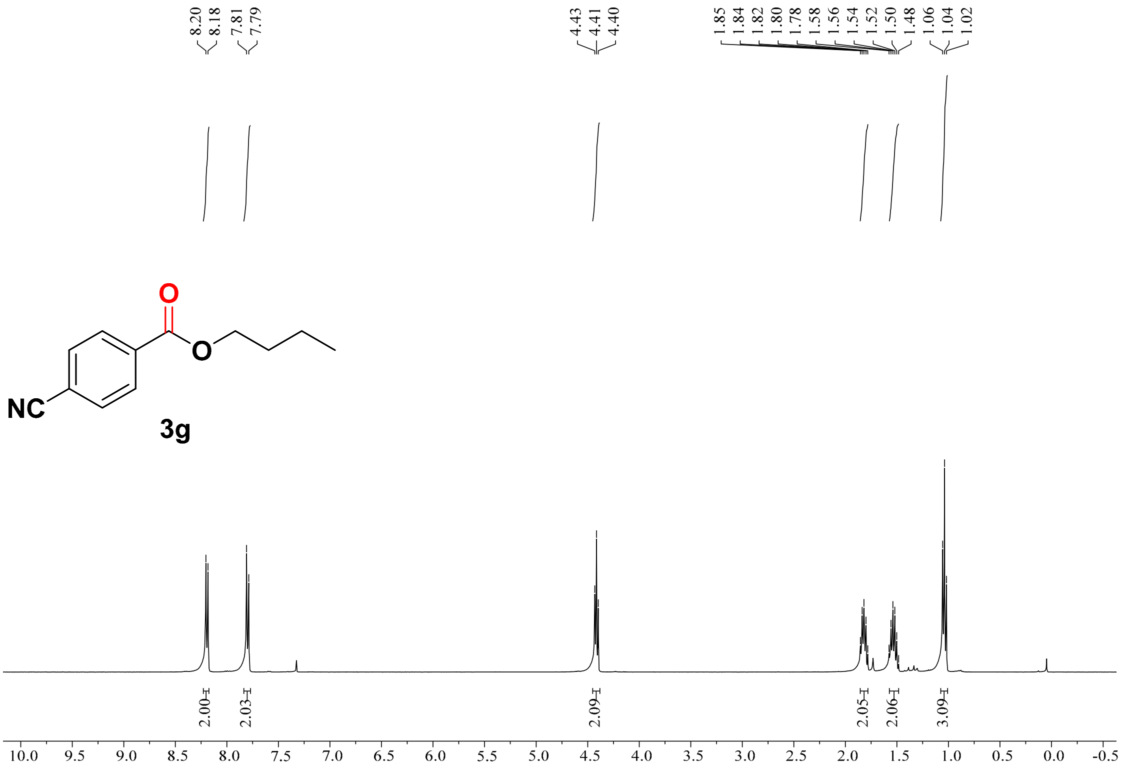
8.20
8.18
7.81
7.79
4.43
4.41
4.40
1.85
1.84
1.82
1.80
1.78
1.58
1.56
1.54
1.52
1.50
1.48
1.06
1.04
1.02
NC
O
O
3g
2.00
2.03
2.09
2.05
2.06
3.09
10.0 9.5 9.0 8.5 8.0 7.5 7.0 6.5 6.0 5.5 5.0 4.5 4.0 3.5 3.0 2.5 2.0 1.5 1.0 0.5 0.0 -0.5

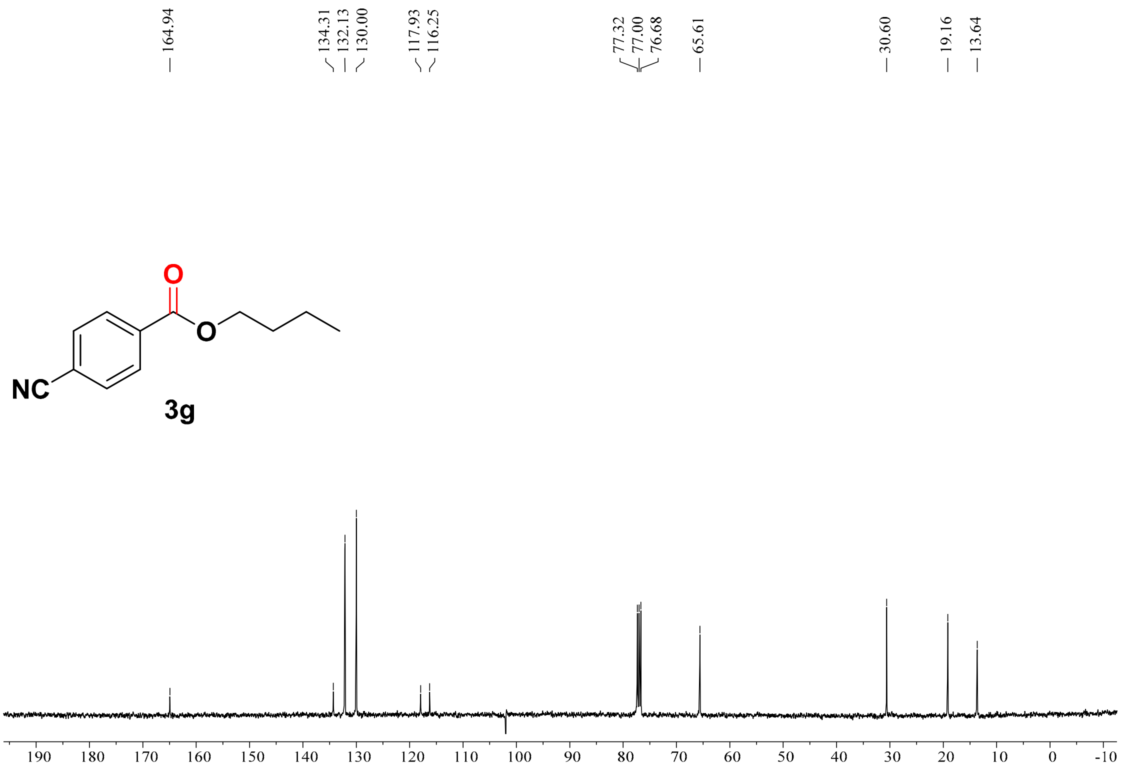
164.94
134.31
132.13
130.00
117.93
116.25
77.32
77.00
76.68
65.61
30.60
19.16
13.64
NC
O
O
3g
190 180 170 160 150 140 130 120 110 100 90 80 70 60 50 40 30 20 10 0 -10

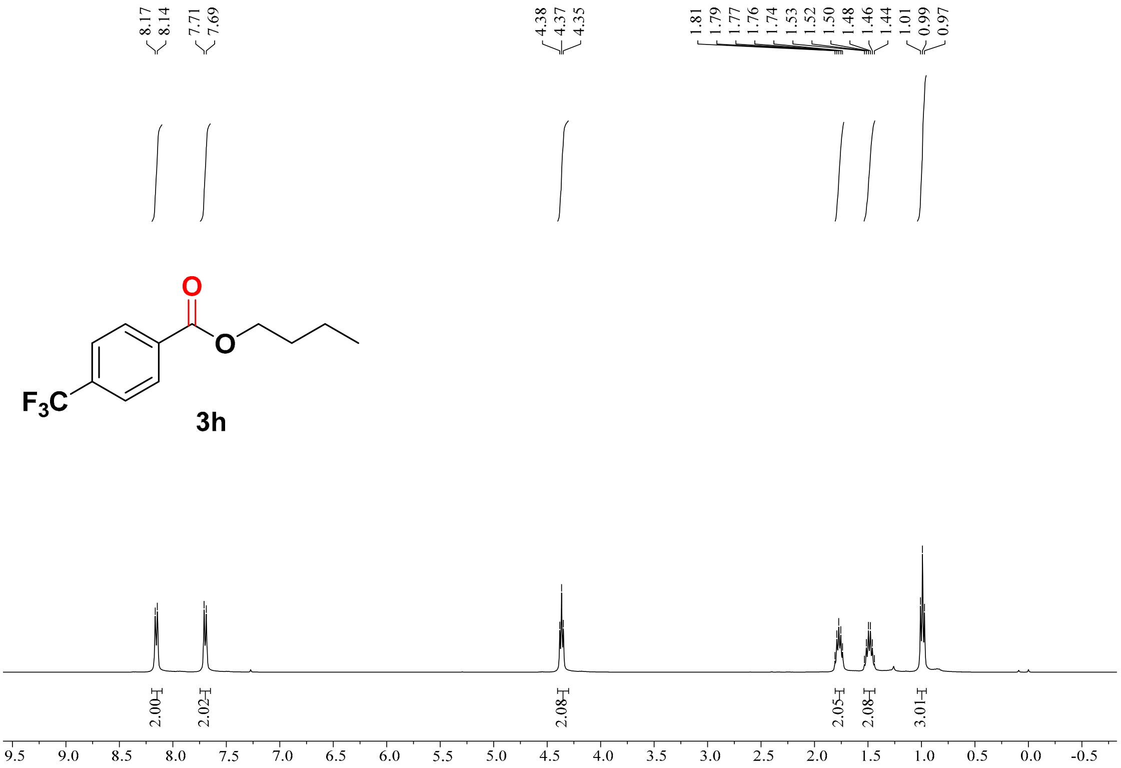

8.17
8.14
7.71
7.69
4.38
4.37
4.35
1.81
1.79
1.77
1.76
1.74
1.53
1.52
1.50
1.48
1.46
1.44
1.01
0.99
0.97
F3C
O
O
3h
2.00
2.02
2.08
2.05
2.08
3.01
9.5
9.0
8.5
8.0
7.5
7.0
6.5
6.0
5.5
5.0
4.5
4.0
3.5
3.0
2.5
2.0
1.5
1.0
0.5
0.0
-0.5

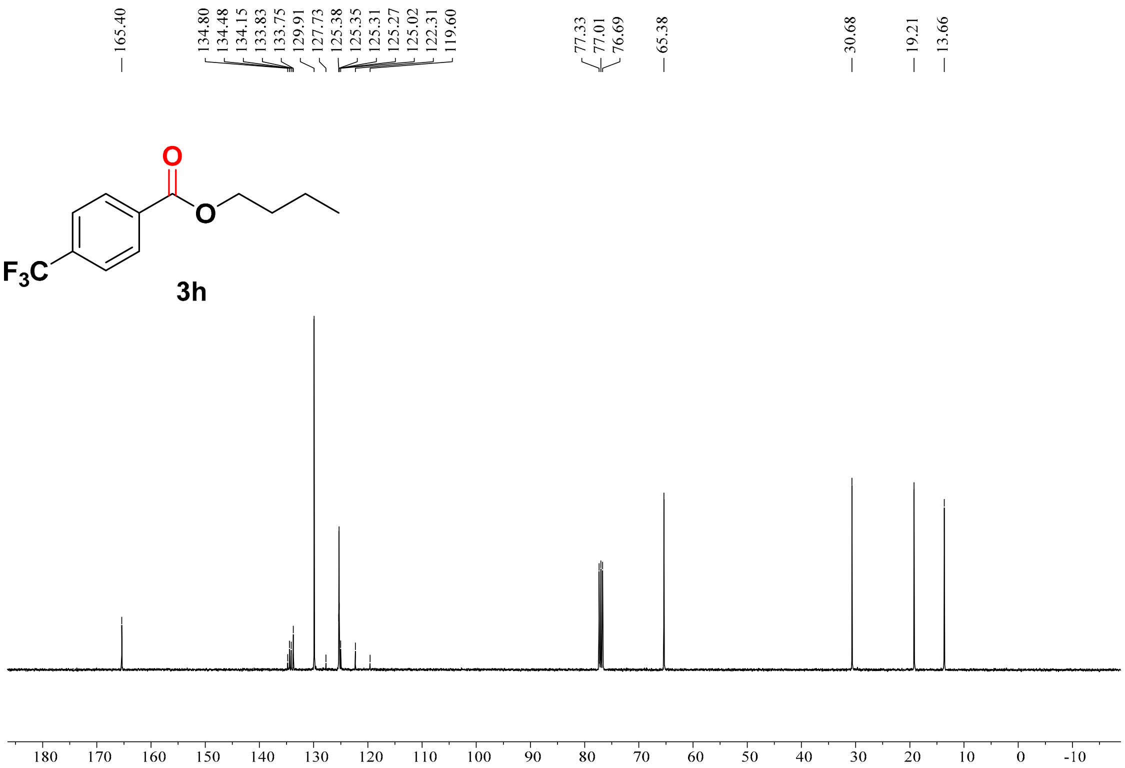

165.40
134.80
134.48
134.15
133.83
133.75
129.91
127.73
125.38
125.35
125.31
125.27
125.02
122.31
119.60
77.33
77.01
76.69
65.38
30.68
19.21
13.66
F3C
O
O
3h
180
170
160
150
140
130
120
110
100
90
80
70
60
50
40
30
20
10
0
-10

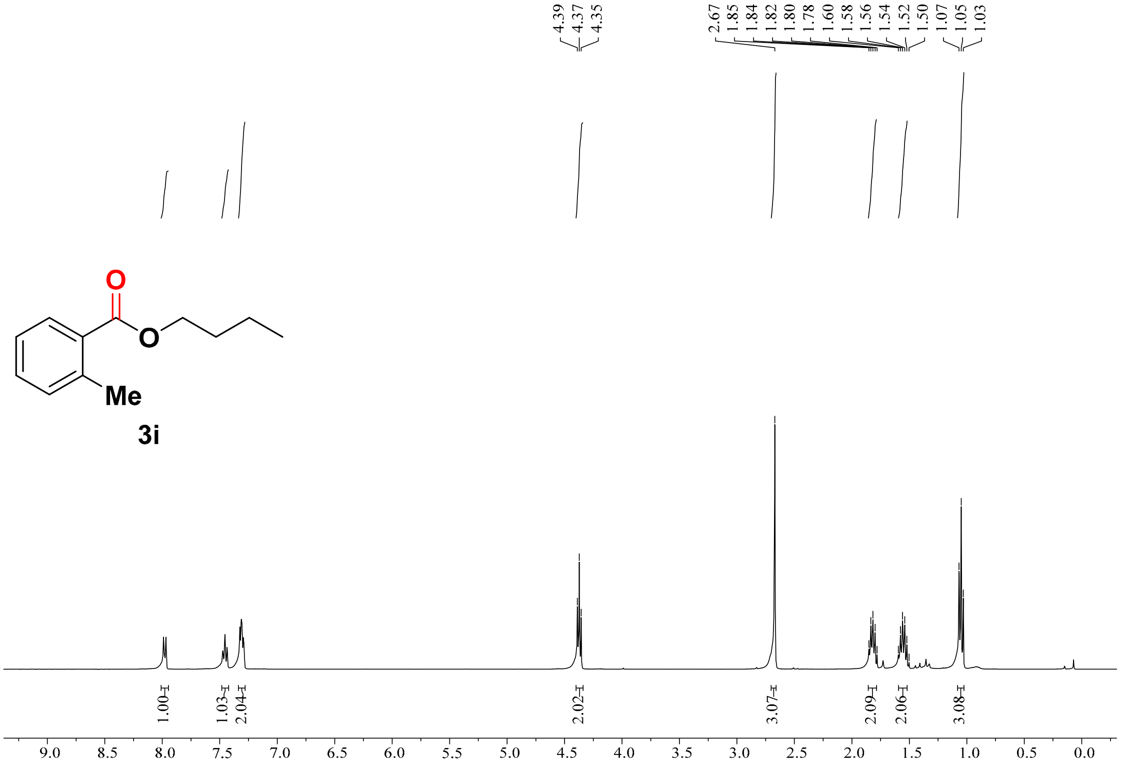
4.39
4.37
4.35
2.67
1.85
1.84
1.82
1.80
1.78
1.60
1.58
1.56
1.54
1.52
1.50
1.07
1.05
1.03
O
O
Me
3i
1.00
1.03
2.04
2.02
3.07
2.09
2.06
3.08
9.0 8.5 8.0 7.5 7.0 6.5 6.0 5.5 5.0 4.5 4.0 3.5 3.0 2.5 2.0 1.5 1.0 0.5 0.0

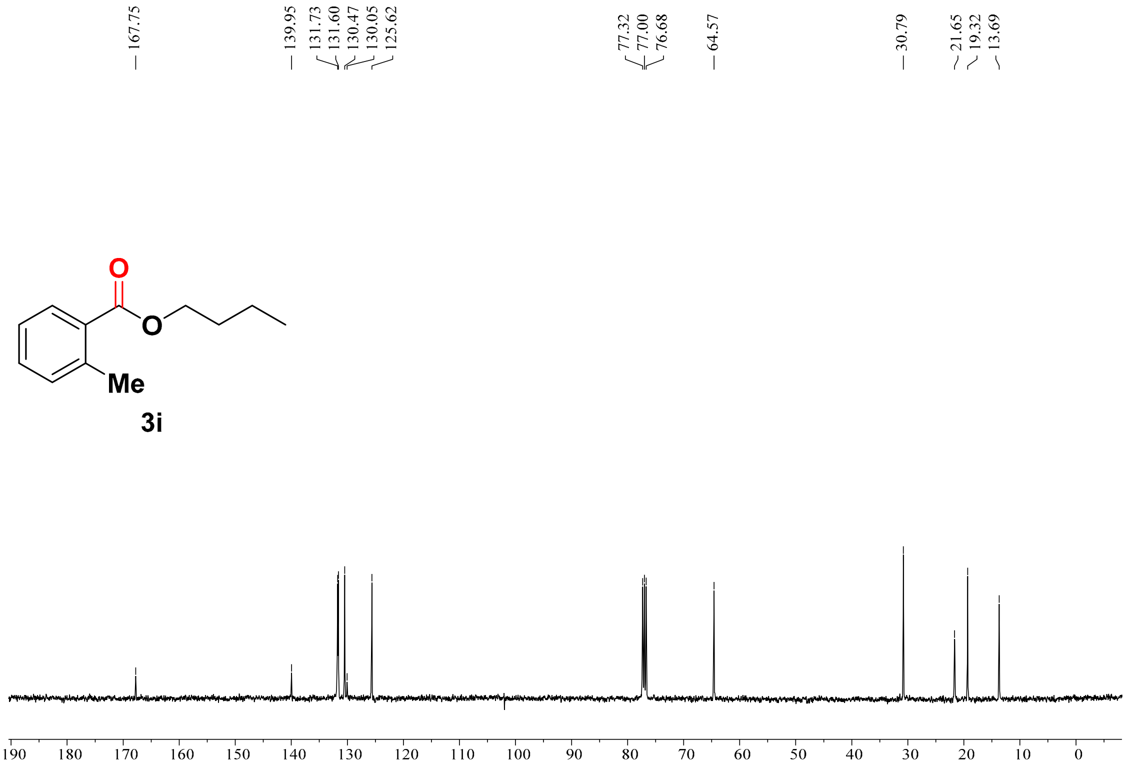
167.75
139.95
131.73
131.60
130.47
130.05
125.62
77.32
77.00
76.68
64.57
30.79
21.65
19.32
13.69
O
O
Me
3i
190 180 170 160 150 140 130 120 110 100 90 80 70 60 50 40 30 20 10 0

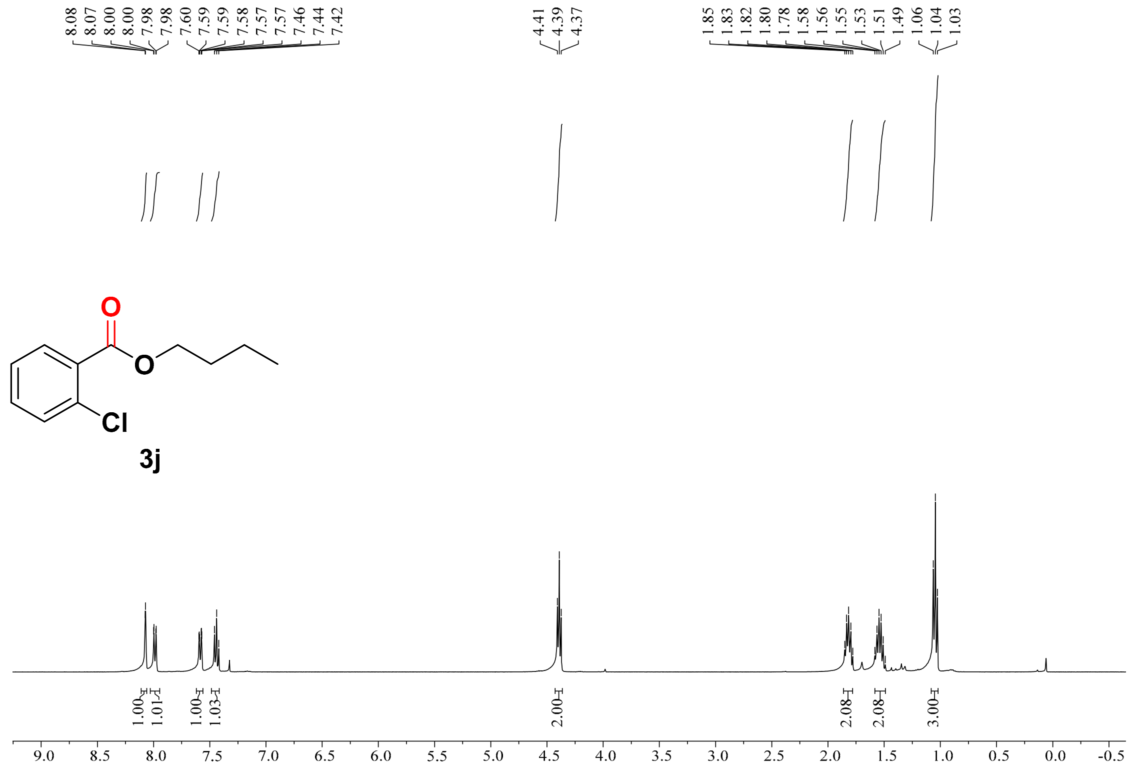
8.08
8.07
8.00
8.00
7.98
7.98
7.60
7.59
7.59
7.58
7.57
7.57
7.46
7.44
7.42
4.41
4.39
4.37
1.85
1.83
1.82
1.80
1.78
1.58
1.56
1.55
1.53
1.51
1.49
1.06
1.04
1.03
O
O
Cl
1.00
1.01
1.00
1.03
2.00
2.08
2.08
3.00
9.0
8.5
8.0
7.5
7.0
6.5
6.0
5.5
5.0
4.5
4.0
3.5
3.0
2.5
2.0
1.5
1.0
0.5
0.0
-0.5

3j

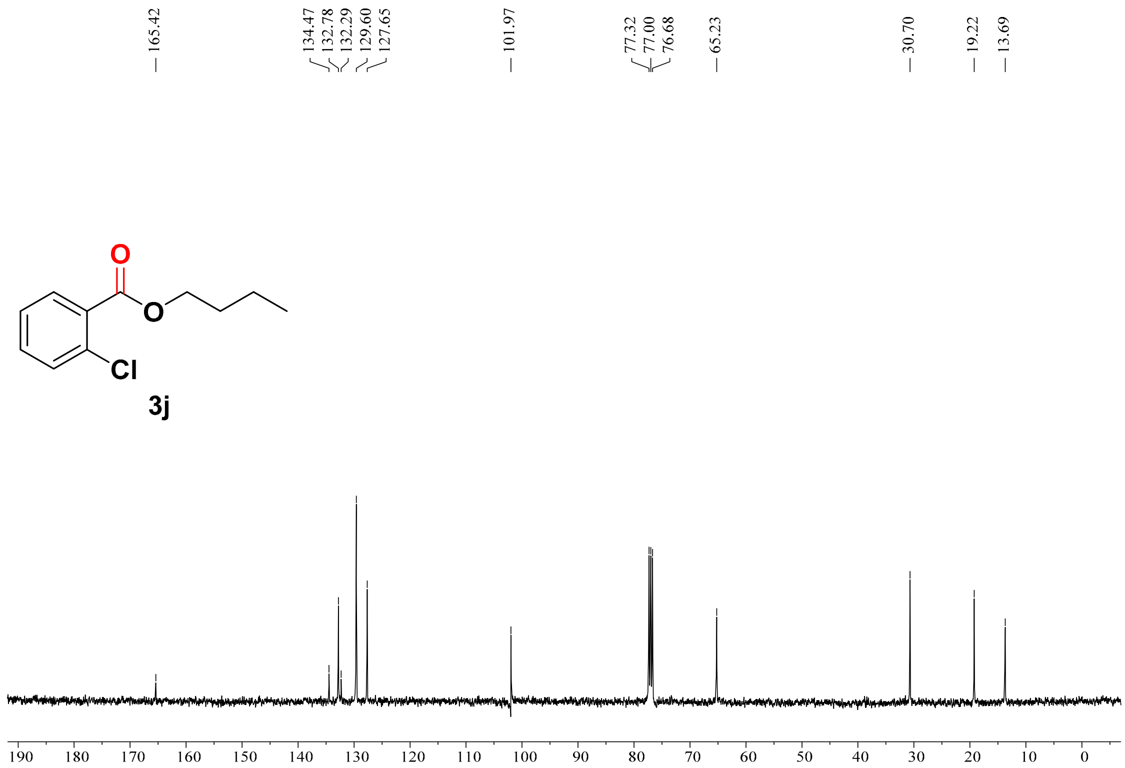
165.42
134.47
132.78
132.29
129.60
127.65
101.97
77.32
77.00
76.68
65.23
30.70
19.22
13.69
O
O
Cl
190
180
170
160
150
140
130
120
110
100
90
80
70
60
50
40
30
20
10
0

3j

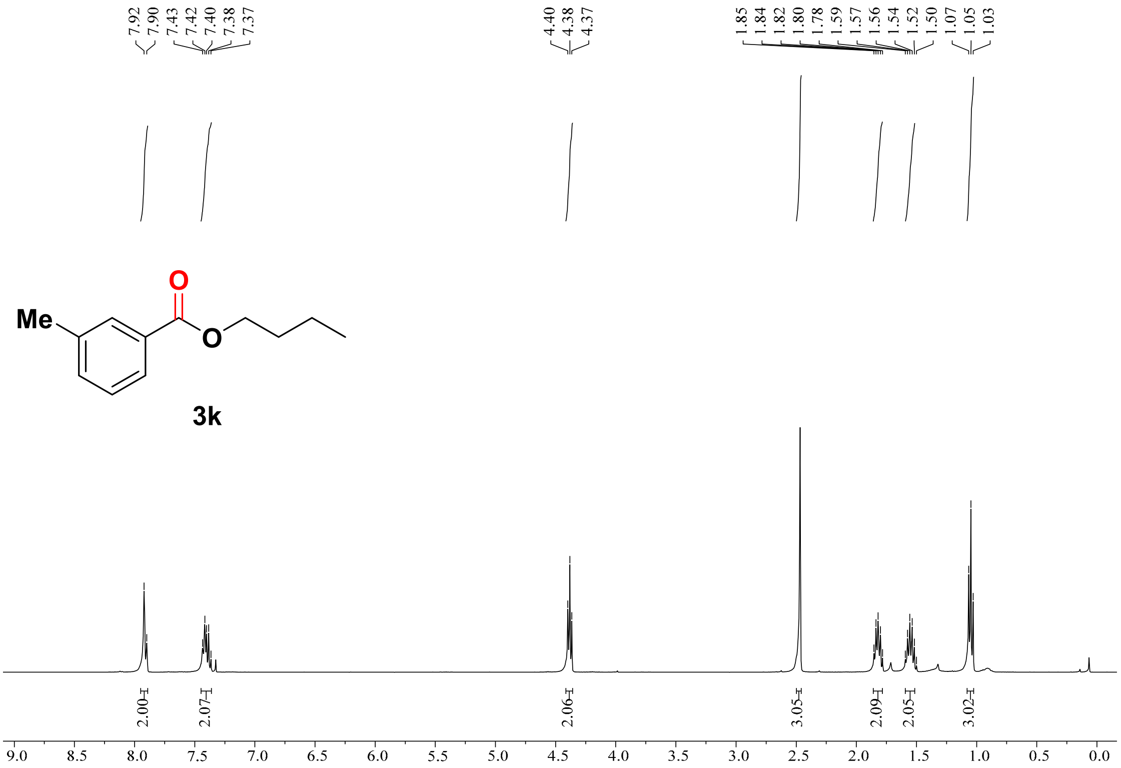
7.92
7.90
7.43
7.42
7.40
7.38
7.37
4.40
4.38
4.37
1.85
1.84
1.82
1.80
1.78
1.59
1.57
1.56
1.54
1.52
1.50
1.07
1.05
1.03
Me
O
O
2.00
2.07
2.06
3.05
2.09
2.05
3.02
9.0 8.5 8.0 7.5 7.0 6.5 6.0 5.5 5.0 4.5 4.0 3.5 3.0 2.5 2.0 1.5 1.0 0.5 0.0

3k

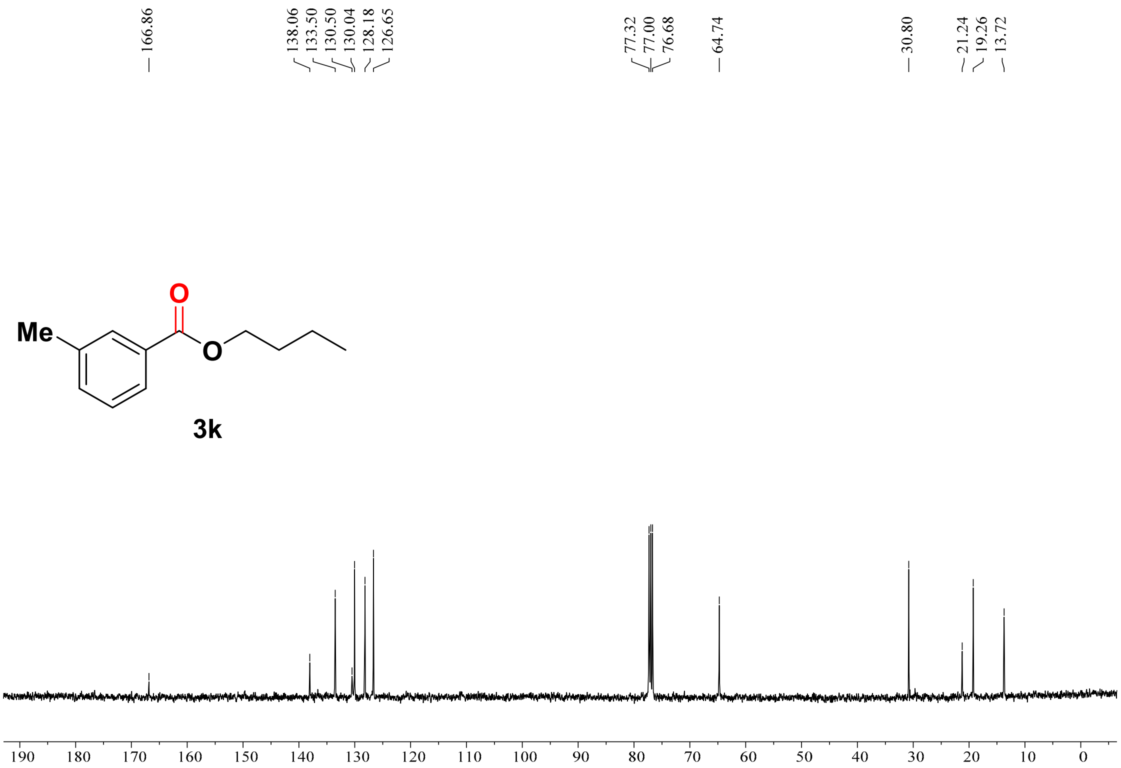
166.86
138.06
133.50
130.50
130.04
128.18
126.65
77.32
77.00
76.68
64.74
30.80
21.24
19.26
13.72
Me
O
O
190 180 170 160 150 140 130 120 110 100 90 80 70 60 50 40 30 20 10 0

3k

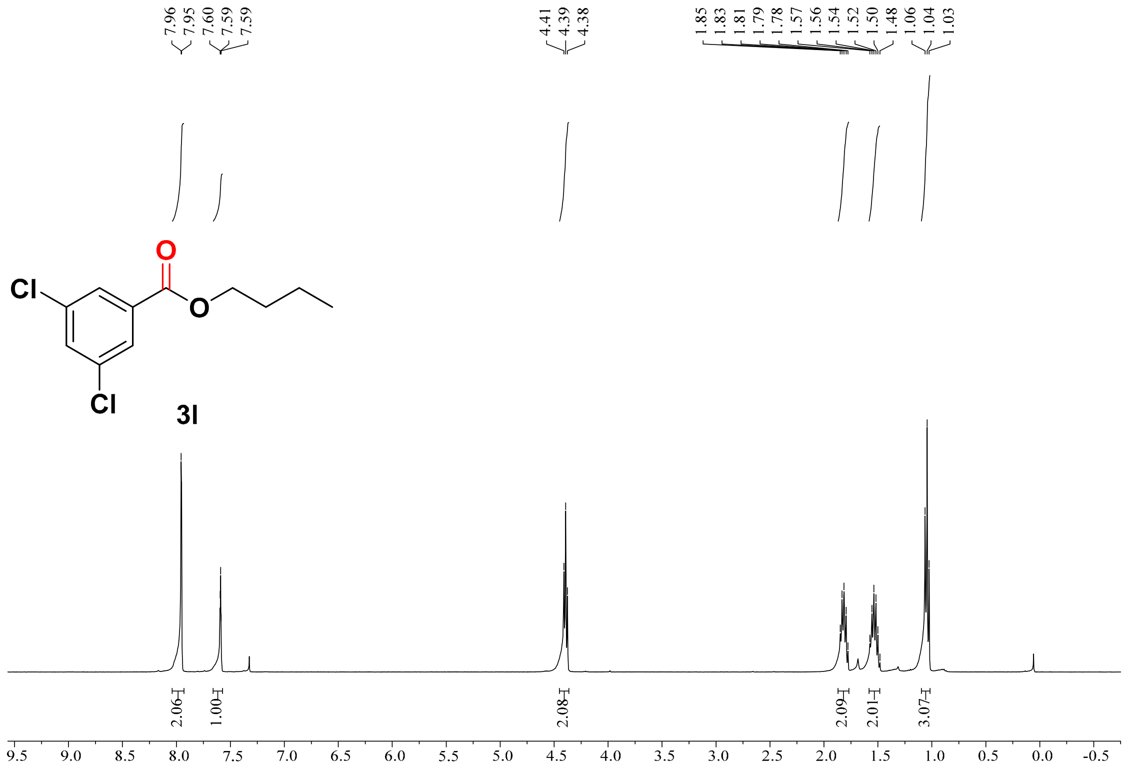
7.96
7.95
7.60
7.59
7.59
4.41
4.39
4.38
1.85
1.83
1.81
1.79
1.78
1.57
1.56
1.54
1.52
1.50
1.48
1.06
1.04
1.03
Cl
O
O
Cl
3l
2.06
1.00
2.08
2.09
2.01
3.07
9.5 9.0 8.5 8.0 7.5 7.0 6.5 6.0 5.5 5.0 4.5 4.0 3.5 3.0 2.5 2.0 1.5 1.0 0.5 0.0 -0.5

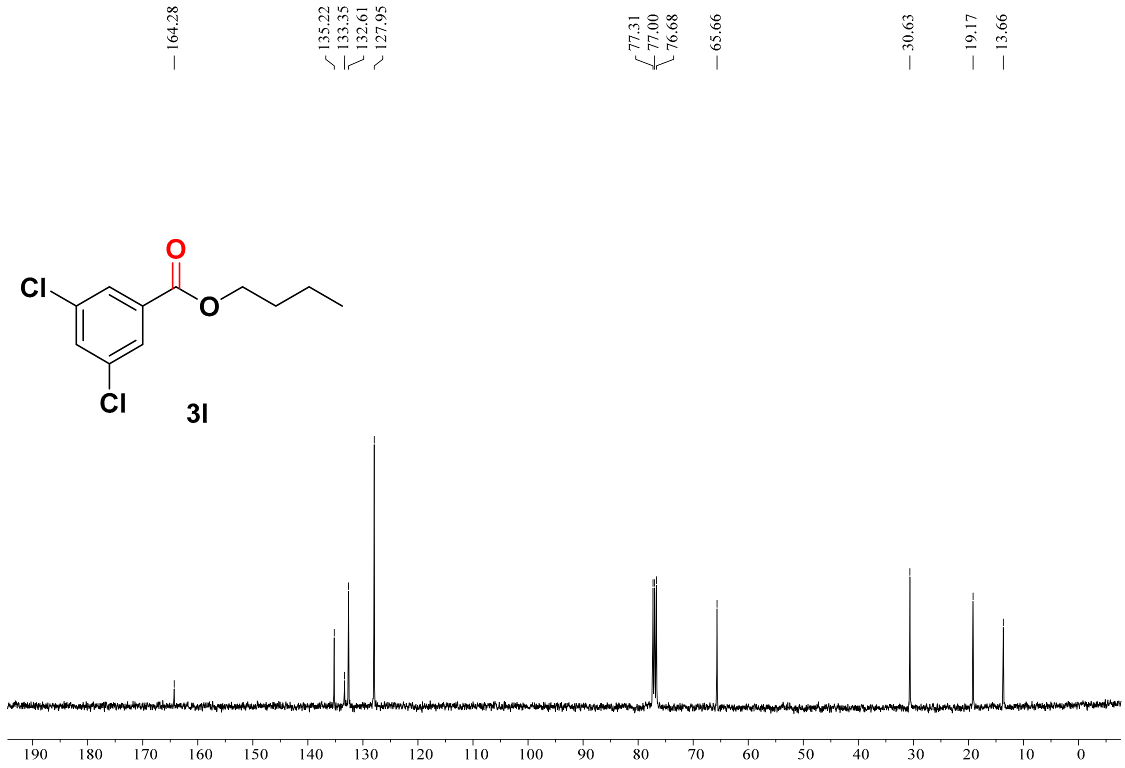
164.28
135.22
133.35
132.61
127.95
77.31
77.00
76.68
65.66
30.63
19.17
13.66
Cl
O
O
Cl
3l
190 180 170 160 150 140 130 120 110 100 90 80 70 60 50 40 30 20 10 0

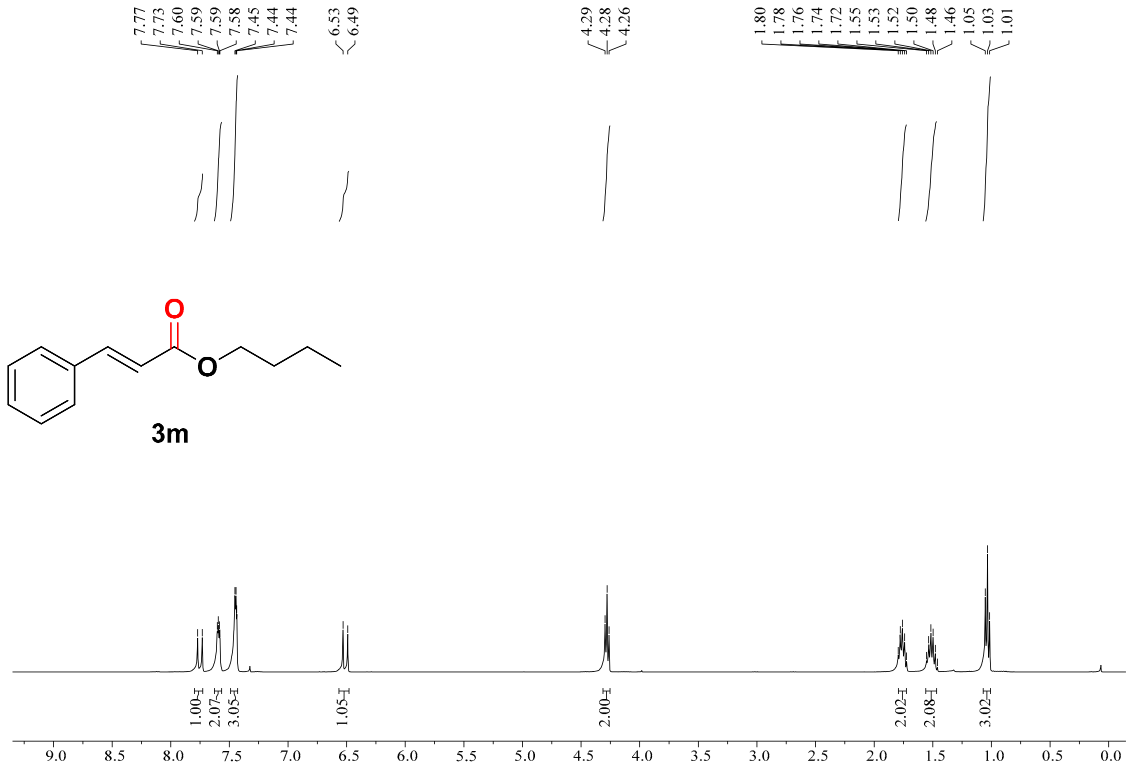
7.77
7.73
7.60
7.59
7.59
7.58
7.45
7.44
7.44
6.53
6.49
4.29
4.28
4.26
1.80
1.78
1.76
1.74
1.72
1.55
1.53
1.52
1.50
1.48
1.46
1.05
1.03
1.01
O
O
3m
1.00
2.07
3.05
1.05
2.00
2.02
2.08
3.02
9.0 8.5 8.0 7.5 7.0 6.5 6.0 5.5 5.0 4.5 4.0 3.5 3.0 2.5 2.0 1.5 1.0 0.5 0.0

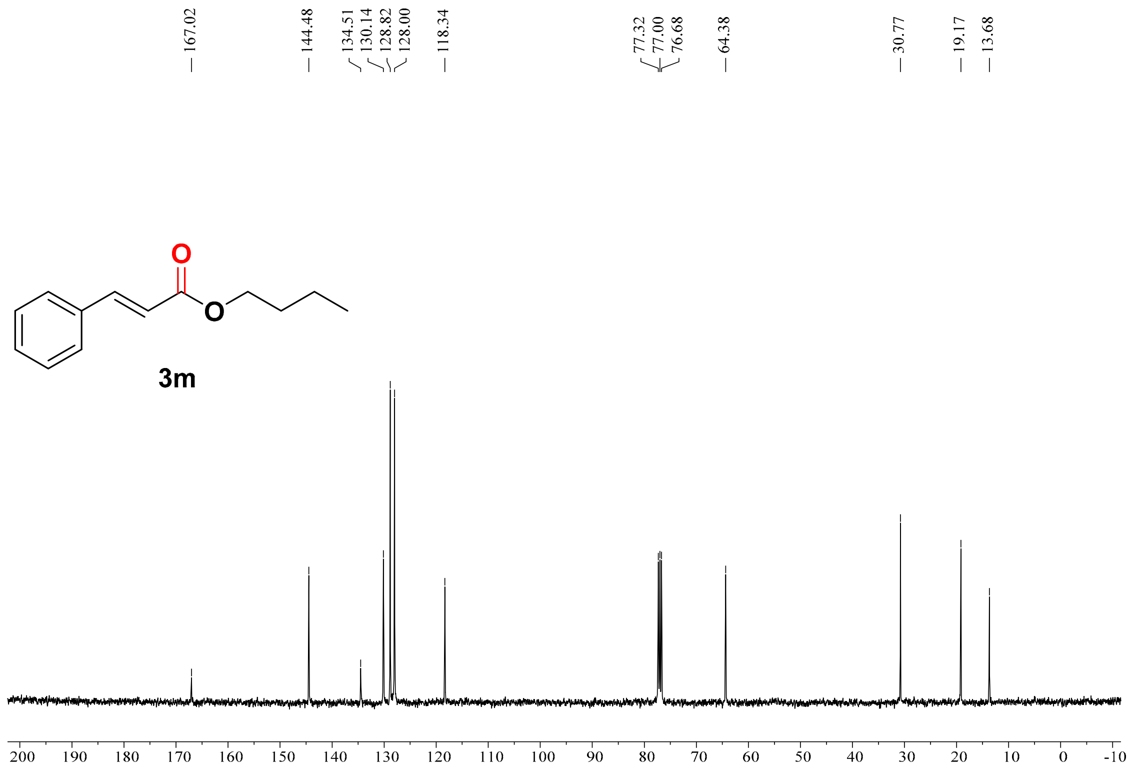
167.02
144.48
134.51
130.14
128.82
128.00
118.34
77.32
77.00
76.68
64.38
30.77
19.17
13.68
O
O
3m
200 190 180 170 160 150 140 130 120 110 100 90 80 70 60 50 40 30 20 10 0 -10

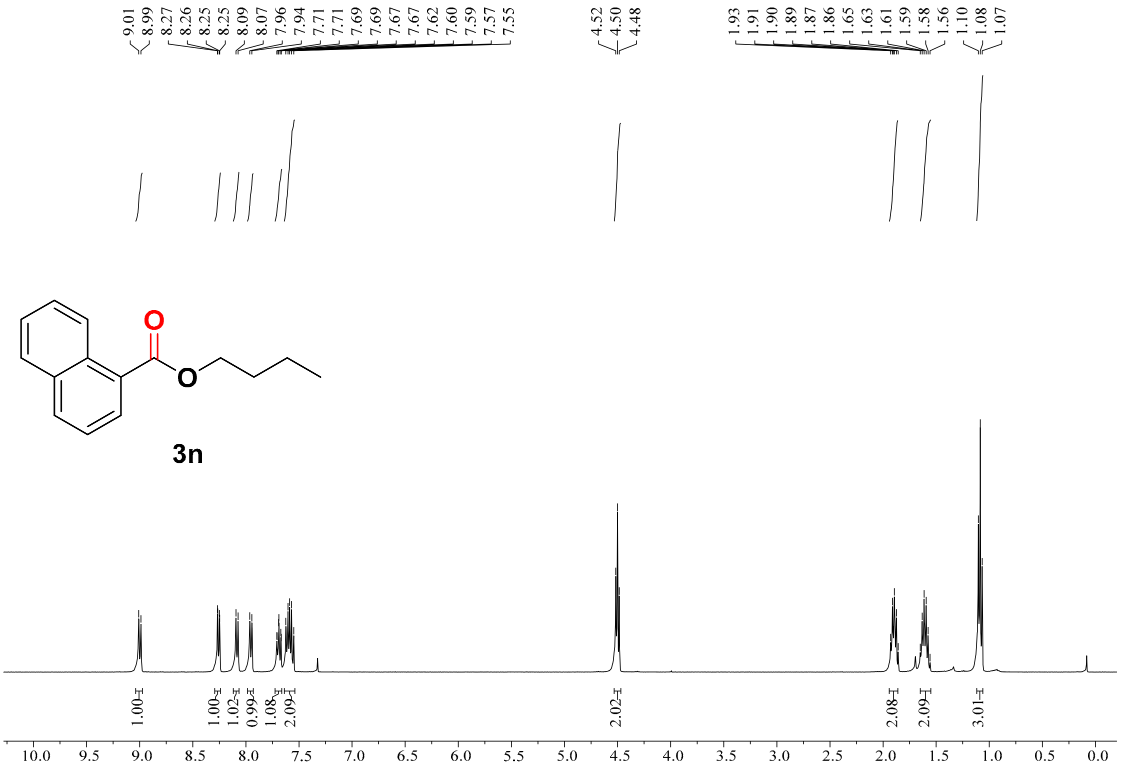
9.01
8.99
8.27
8.26
8.25
8.25
8.09
8.07
7.96
7.94
7.71
7.71
7.69
7.69
7.67
7.67
7.62
7.60
7.59
7.57
7.55
4.52
4.50
4.48
1.93
1.91
1.90
1.89
1.87
1.86
1.65
1.63
1.61
1.59
1.58
1.56
1.10
1.08
1.07
O
O
3n
1.00
1.00
1.02
0.99
1.08
2.09
2.02
2.08
2.09
3.01
10.0 9.5 9.0 8.5 8.0 7.5 7.0 6.5 6.0 5.5 5.0 4.5 4.0 3.5 3.0 2.5 2.0 1.5 1.0 0.5 0.0

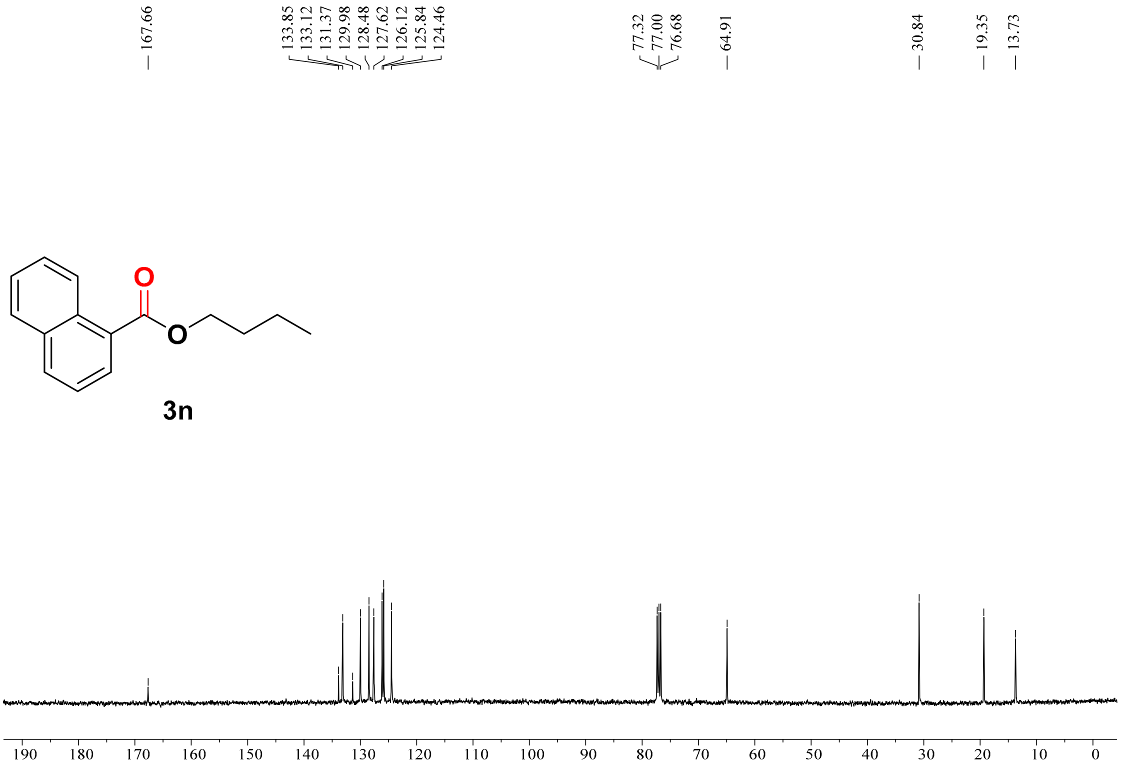
167.66
133.85
133.12
131.37
129.98
128.48
127.62
126.12
125.84
124.46
77.32
77.00
76.68
64.91
30.84
19.35
13.73
O
O
3n
190 180 170 160 150 140 130 120 110 100 90 80 70 60 50 40 30 20 10 0

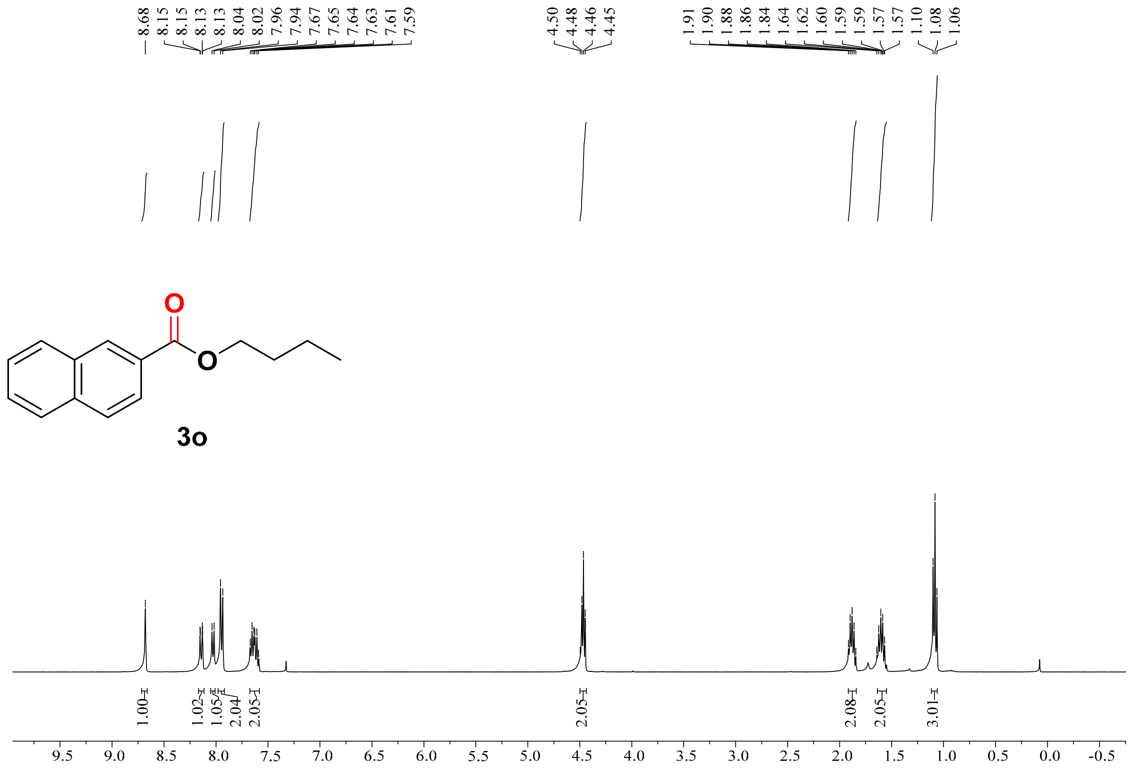
8.68
8.15
8.15
8.13
8.13
8.04
8.02
7.96
7.94
7.67
7.65
7.64
7.63
7.61
7.59
4.50
4.48
4.46
4.45
1.91
1.90
1.88
1.86
1.84
1.64
1.62
1.60
1.59
1.59
1.57
1.57
1.10
1.08
1.06
3o
1.00
1.02
1.05
2.04
2.05
2.05
2.08
2.05
3.01
9.5 9.0 8.5 8.0 7.5 7.0 6.5 6.0 5.5 5.0 4.5 4.0 3.5 3.0 2.5 2.0 1.5 1.0 0.5 0.0 -0.5

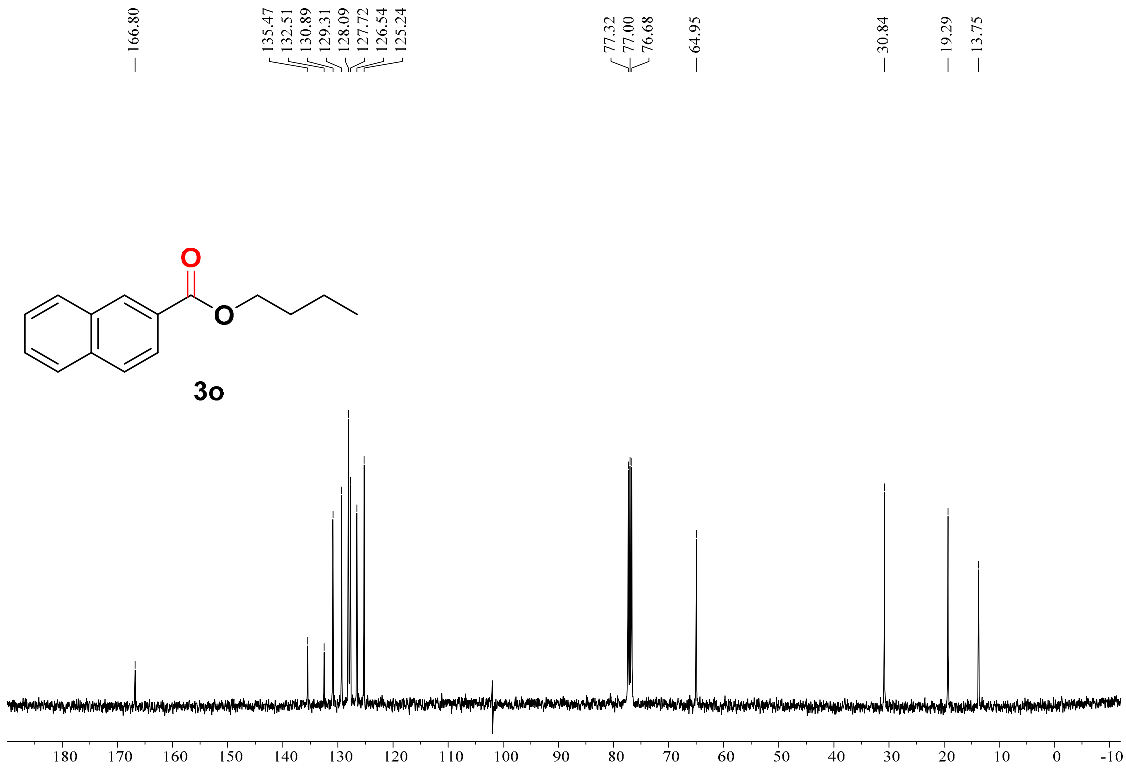
166.80
135.47
132.51
130.89
129.31
128.09
127.72
126.54
125.24
77.32
77.00
76.68
64.95
30.84
19.29
13.75
3o
180 170 160 150 140 130 120 110 100 90 80 70 60 50 40 30 20 10 0 -10

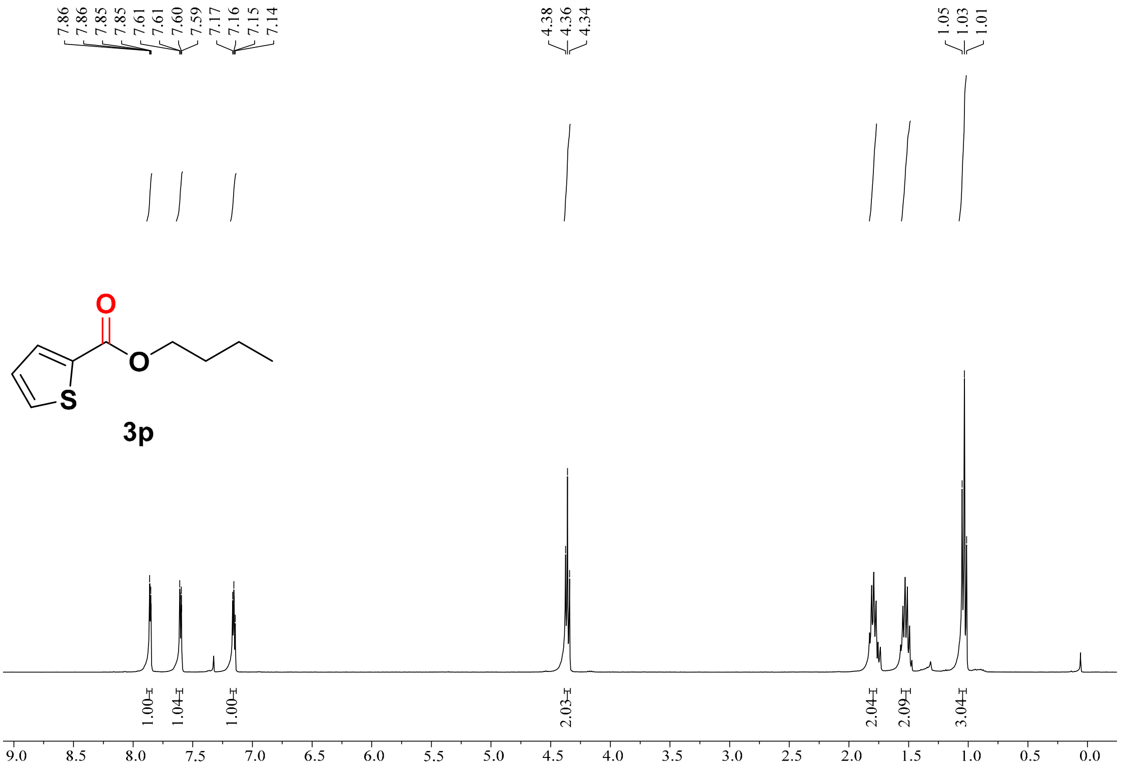
7.86
7.86
7.85
7.85
7.61
7.61
7.60
7.59
7.17
7.16
7.15
7.14
4.38
4.36
4.34
1.05
1.03
1.01
O
O
S
3p
1.00
1.04
1.00
2.03
2.04
2.09
3.04
9.0
8.5
8.0
7.5
7.0
6.5
6.0
5.5
5.0
4.5
4.0
3.5
3.0
2.5
2.0
1.5
1.0
0.5
0.0

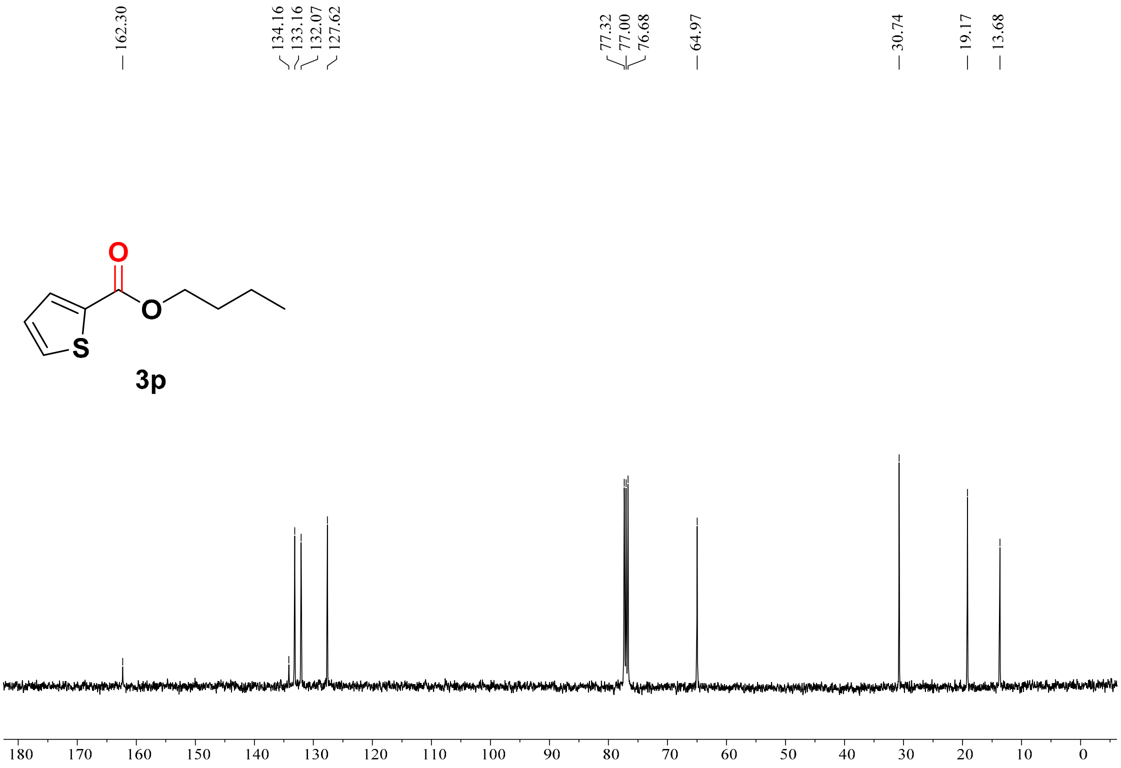
162.30
134.16
133.16
132.07
127.62
77.32
77.00
76.68
64.97
30.74
19.17
13.68
O
O
S
3p
180
170
160
150
140
130
120
110
100
90
80
70
60
50
40
30
20
10
0

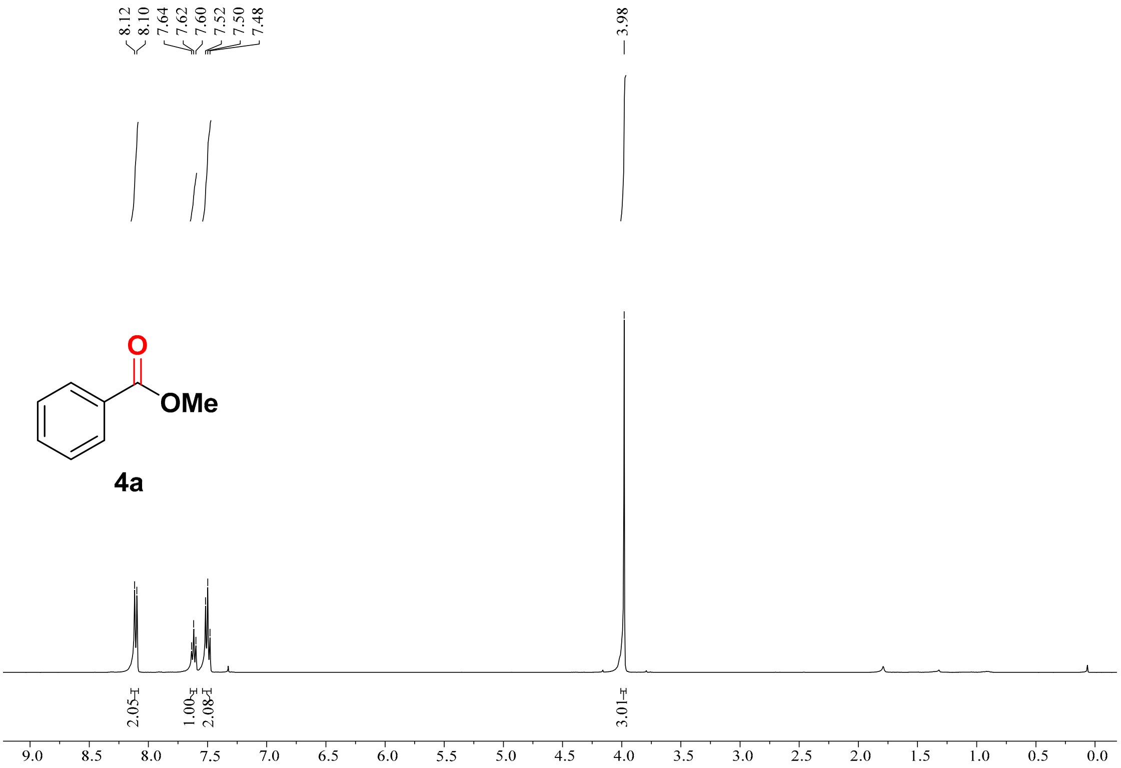

8.12
8.10
7.64
7.62
7.60
7.52
7.50
7.48
3.98
OMe
4a
2.05
1.00
2.08
3.01
9.0
8.5
8.0
7.5
7.0
6.5
6.0
5.5
5.0
4.5
4.0
3.5
3.0
2.5
2.0
1.5
1.0
0.5
0.0

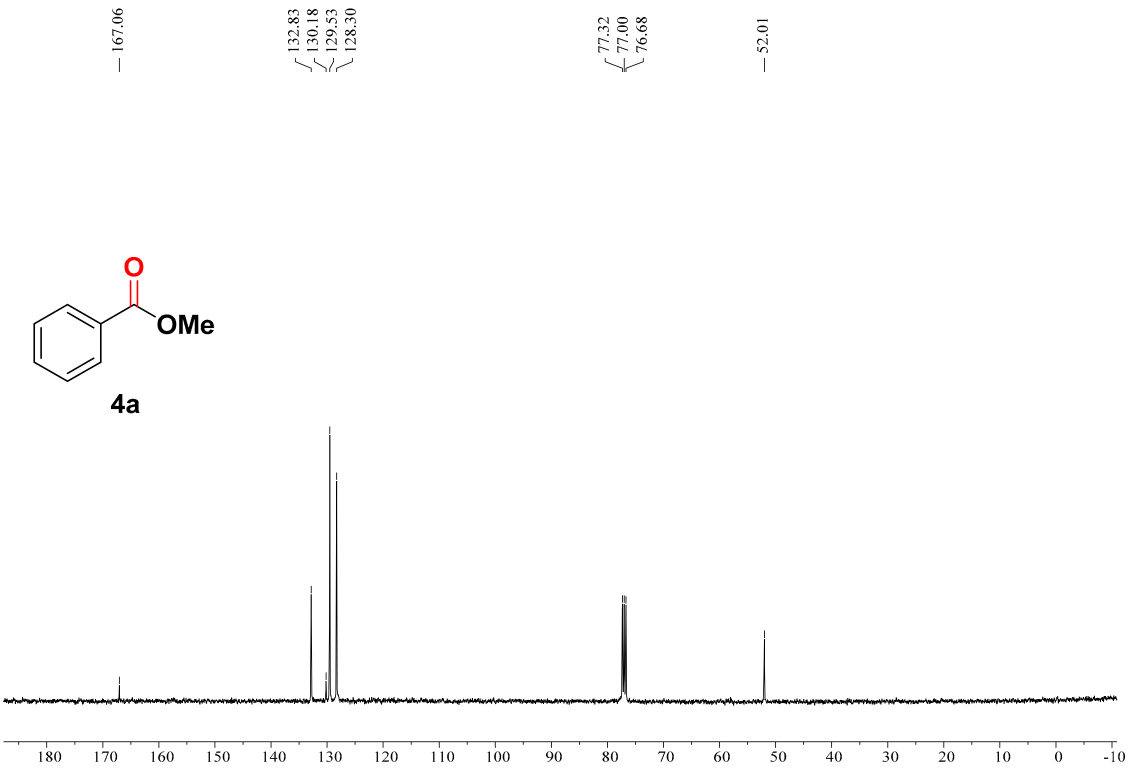

167.06
132.83
130.18
129.53
128.30
77.32
77.00
76.68
52.01
OMe
4a
180
170
160
150
140
130
120
110
100
90
80
70
60
50
40
30
20
10
0
-10

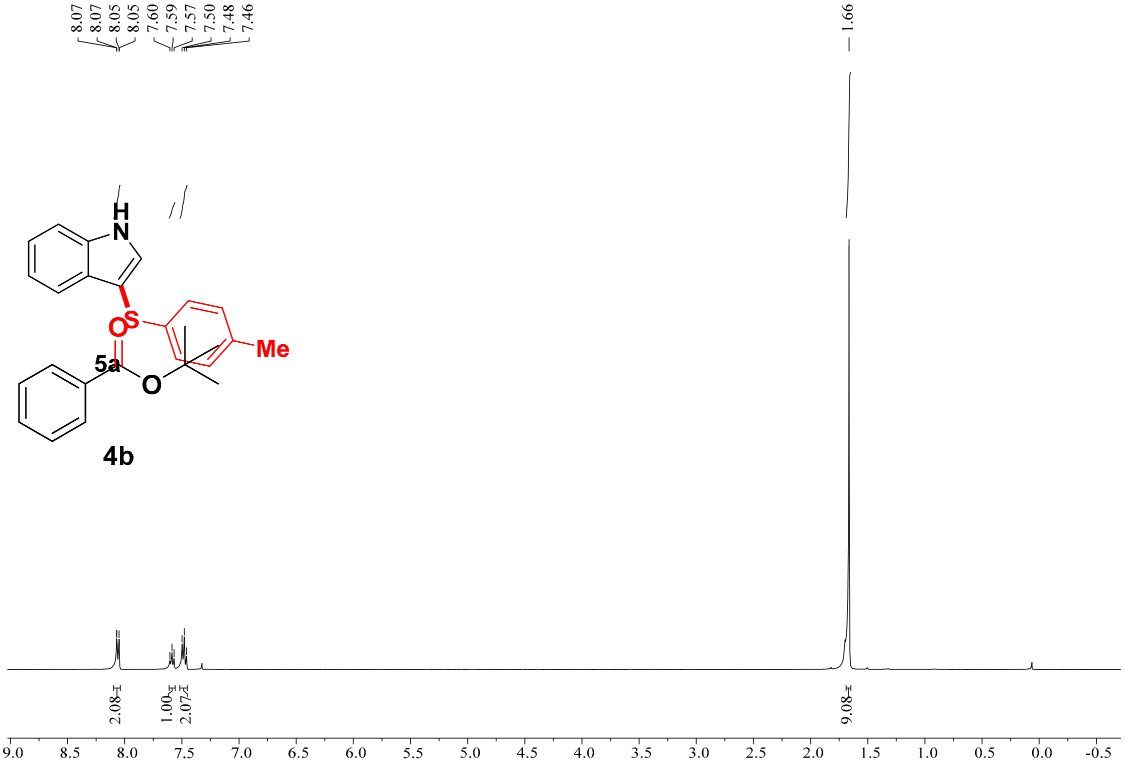
8.07
8.07
8.05
8.05
7.60
7.59
7.57
7.50
7.48
7.46
1.66
H
N
S
Me
5a
O
O
4b
2.08
1.00
2.07
9.08
9.0 8.5 8.0 7.5 7.0 6.5 6.0 5.5 5.0 4.5 4.0 3.5 3.0 2.5 2.0 1.5 1.0 0.5 0.0 -0.5

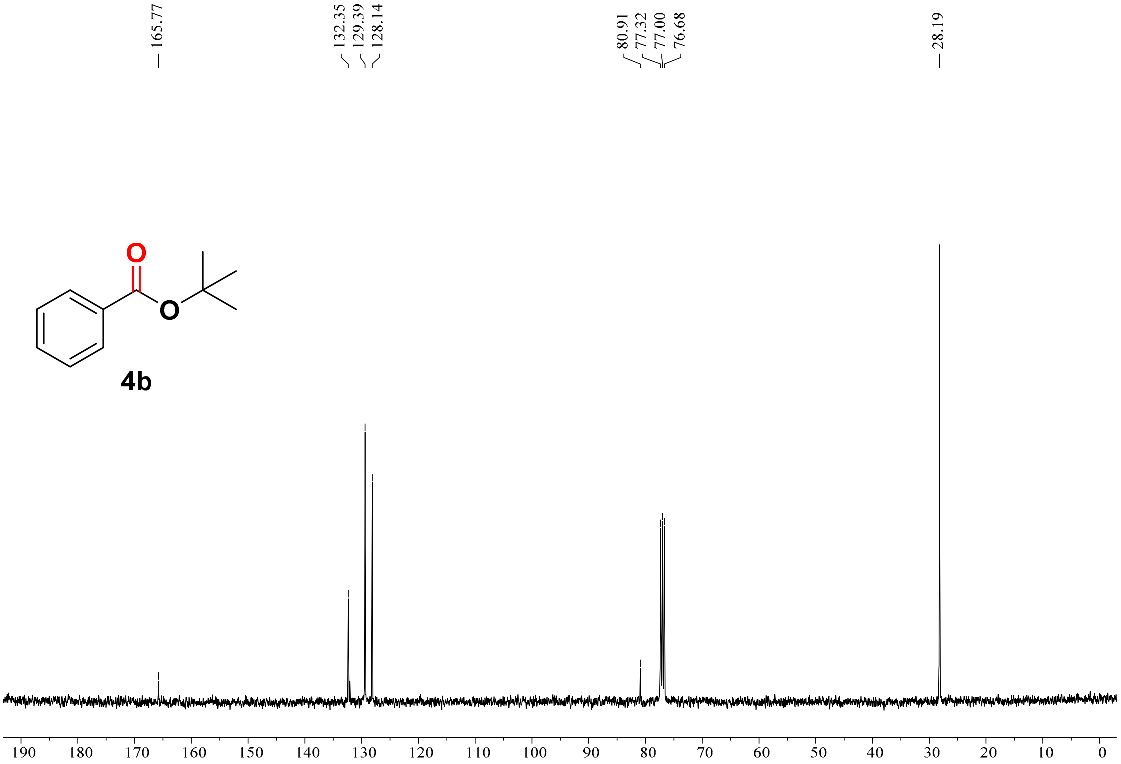
165.77
132.35
129.39
128.14
80.91
77.32
77.00
76.68
28.19
O
O
4b
190 180 170 160 150 140 130 120 110 100 90 80 70 60 50 40 30 20 10 0

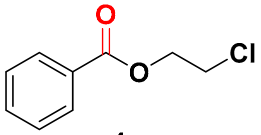
O
O
Cl

4c

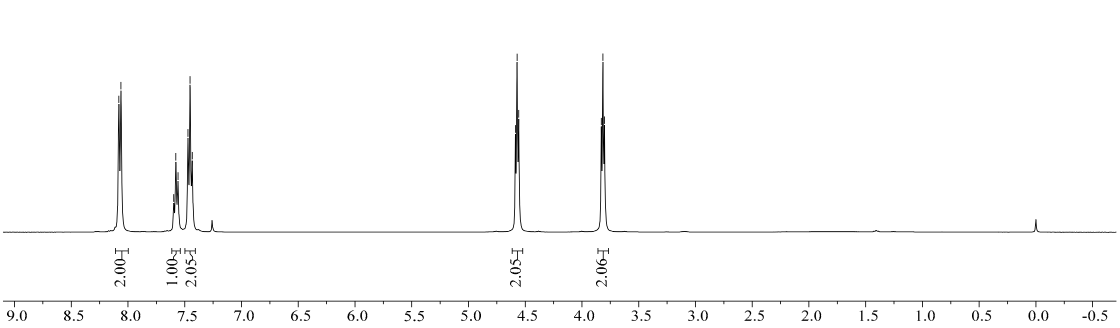
8.08
8.06
7.60
7.58
7.56
7.47
7.45
7.43
4.59
4.57
4.56
3.83
3.82
3.80
2.00
1.00
2.05
2.05
2.06
9.0 8.5 8.0 7.5 7.0 6.5 6.0 5.5 5.0 4.5 4.0 3.5 3.0 2.5 2.0 1.5 1.0 0.5 0.0 -0.5

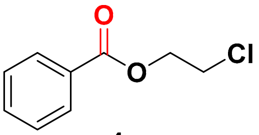
O
O
Cl

4c

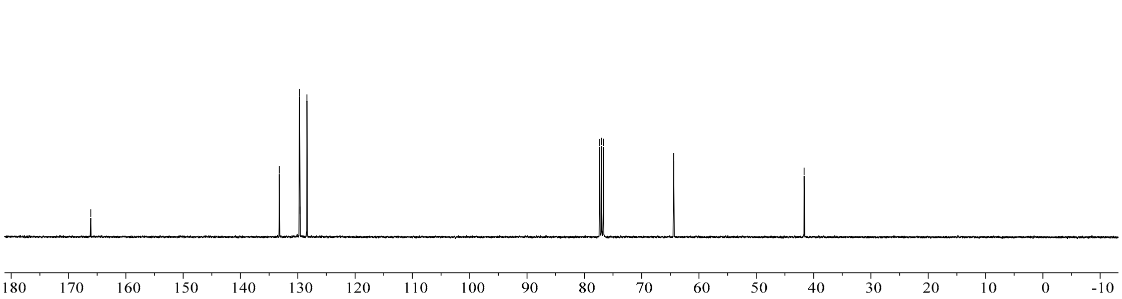
166.14
133.22
129.71
129.60
128.41
77.32
77.00
76.68
64.42
41.63
180 170 160 150 140 130 120 110 100 90 80 70 60 50 40 30 20 10 0 -10

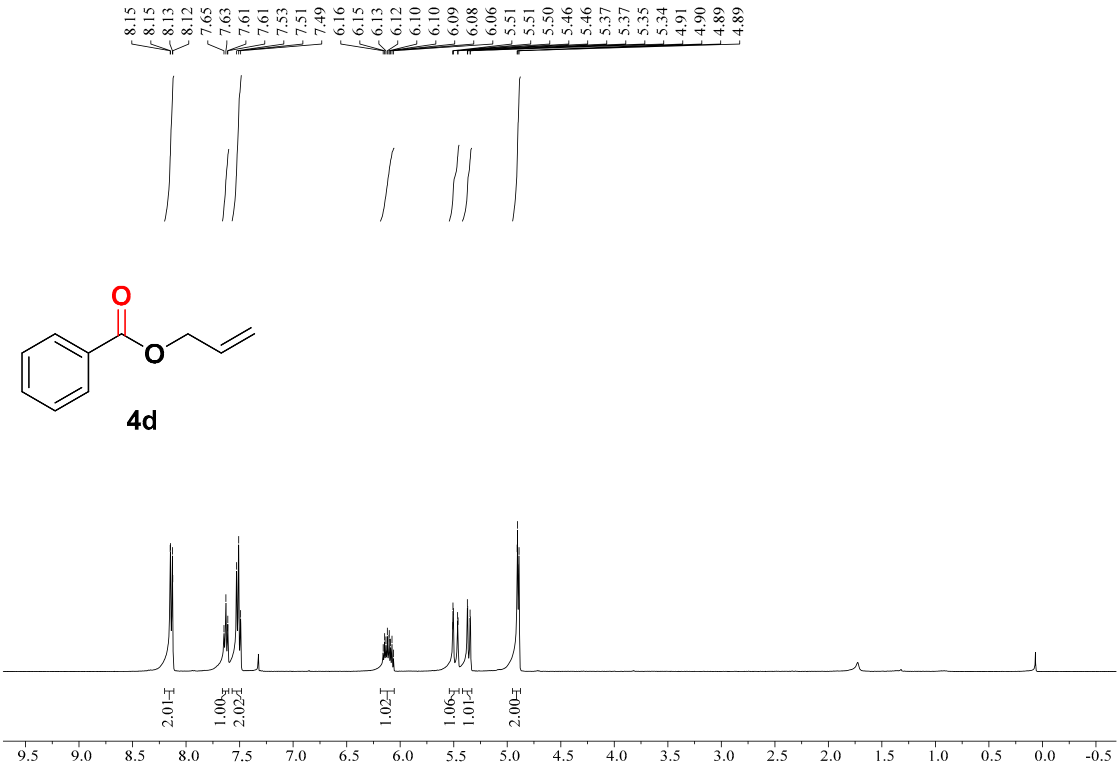
8.15
8.15
8.13
8.12
7.65
7.63
7.61
7.61
7.53
7.51
7.49
6.16
6.15
6.13
6.12
6.10
6.10
6.09
6.08
6.06
5.51
5.51
5.50
5.46
5.46
5.37
5.37
5.35
5.34
4.91
4.90
4.89
4.89
O
O
4d
2.01
1.00
2.02
1.02
1.06
1.01
2.00
9.5 9.0 8.5 8.0 7.5 7.0 6.5 6.0 5.5 5.0 4.5 4.0 3.5 3.0 2.5 2.0 1.5 1.0 0.5 0.0 -0.5

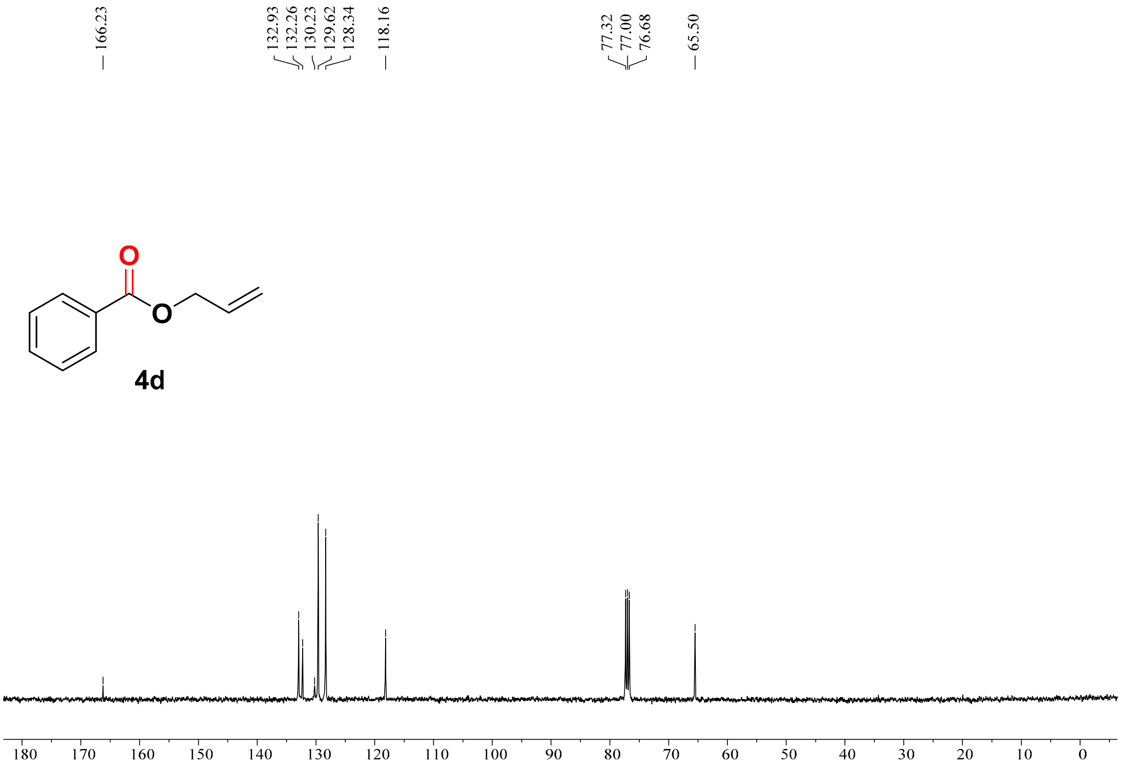
166.23
132.93
132.26
130.23
129.62
128.34
118.16
77.32
77.00
76.68
65.50
O
O
4d
180 170 160 150 140 130 120 110 100 90 80 70 60 50 40 30 20 10 0

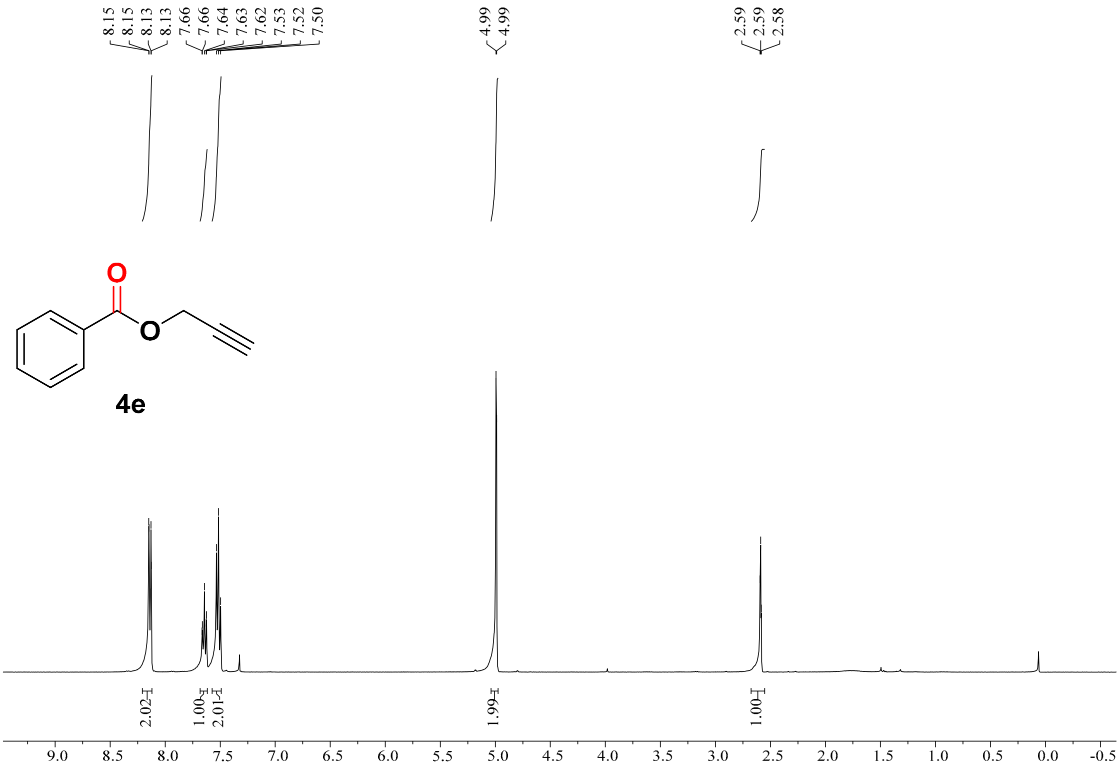
8.15
8.15
8.13
8.13
7.66
7.66
7.64
7.63
7.62
7.53
7.52
7.50
4.99
4.99
2.59
2.59
2.58
4e
2.02
1.00
2.01
1.99
1.00
9.0 8.5 8.0 7.5 7.0 6.5 6.0 5.5 5.0 4.5 4.0 3.5 3.0 2.5 2.0 1.5 1.0 0.5 0.0 -0.5

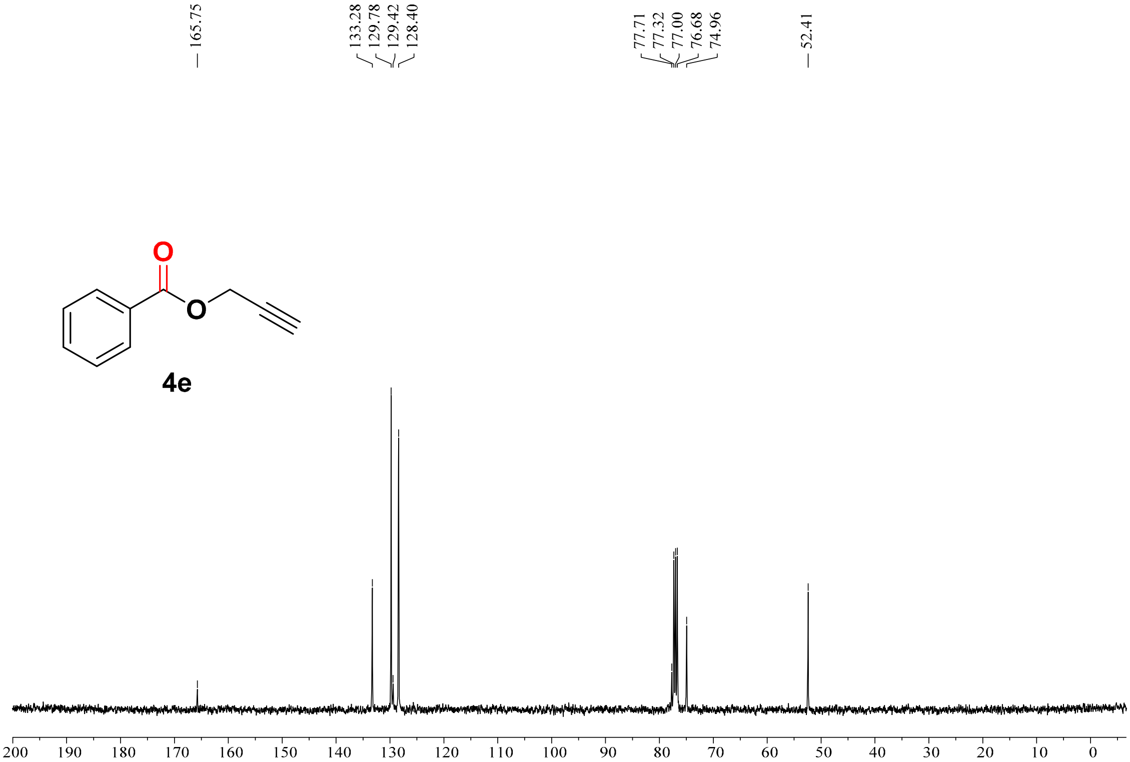
165.75
133.28
129.78
129.42
128.40
77.71
77.32
77.00
76.68
74.96
52.41
4e
200 190 180 170 160 150 140 130 120 110 100 90 80 70 60 50 40 30 20 10 0

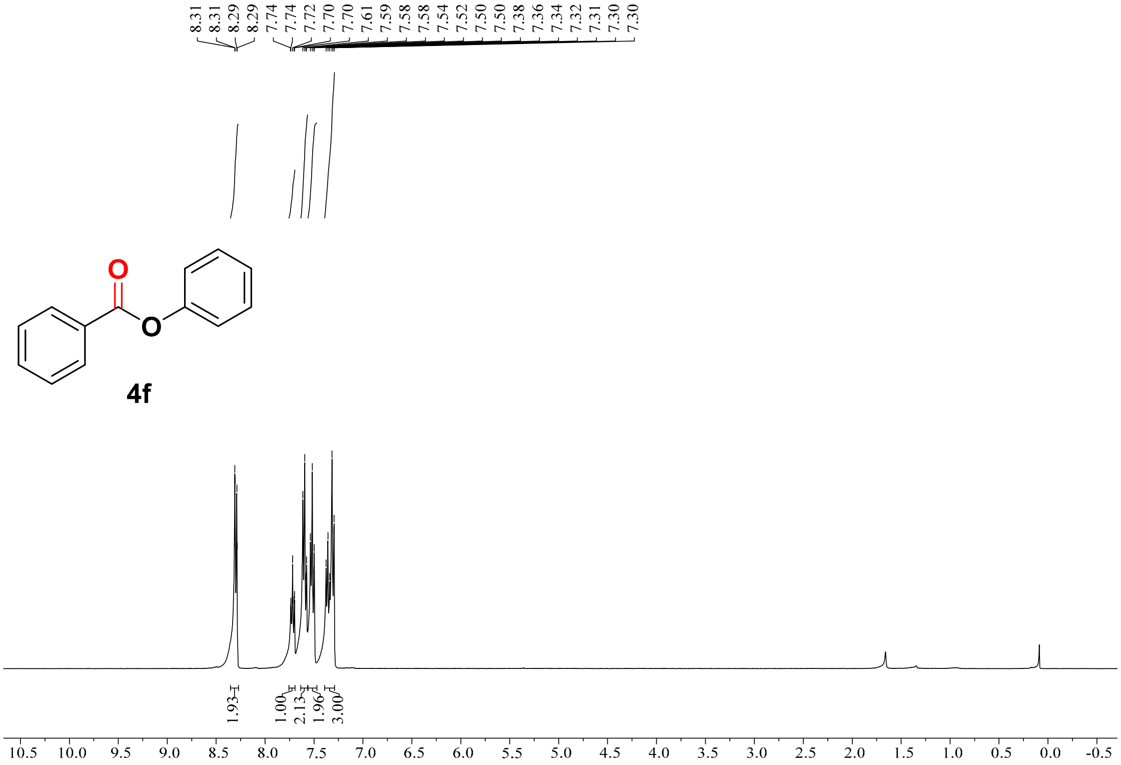
8.31
8.31
8.29
8.29
7.74
7.74
7.72
7.70
7.70
7.61
7.59
7.58
7.58
7.54
7.52
7.50
7.50
7.38
7.36
7.34
7.32
7.31
7.30
7.30
4f
1.93
1.00
2.13
1.96
3.00
10.5 10.0 9.5 9.0 8.5 8.0 7.5 7.0 6.5 6.0 5.5 5.0 4.5 4.0 3.5 3.0 2.5 2.0 1.5 1.0 0.5 0.0 -0.5

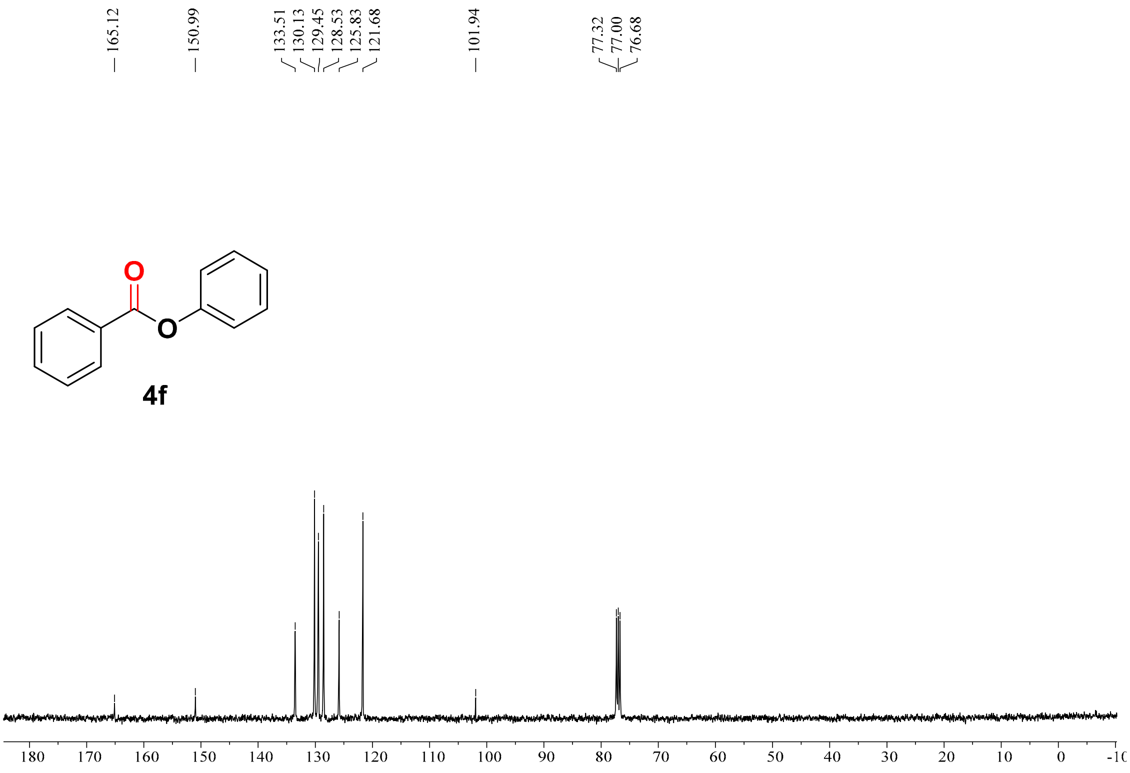
165.12
150.99
133.51
130.13
129.45
128.53
125.83
121.68
101.94
77.32
77.00
76.68
4f
180 170 160 150 140 130 120 110 100 90 80 70 60 50 40 30 20 10 0 -10

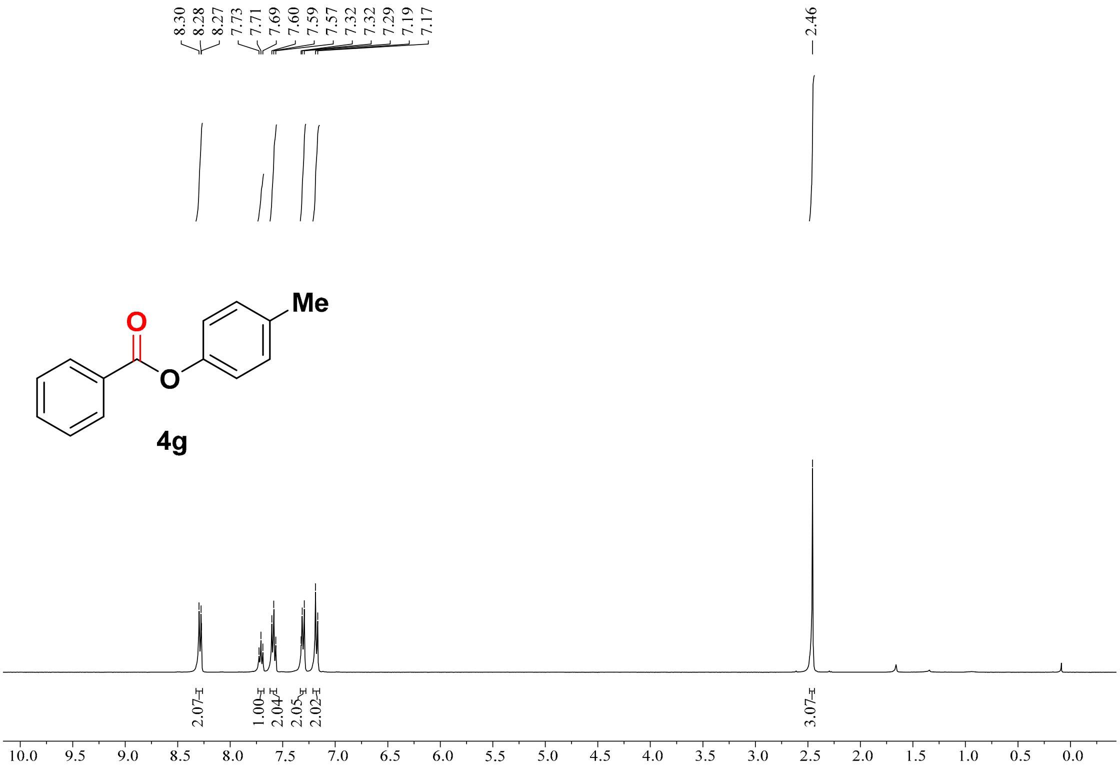

8.30
8.28
8.27
7.73
7.71
7.69
7.60
7.59
7.57
7.32
7.32
7.29
7.19
7.17
2.46
Me
O
O
4g
2.07
1.00
2.04
2.05
2.02
3.07
10.0 9.5 9.0 8.5 8.0 7.5 7.0 6.5 6.0 5.5 5.0 4.5 4.0 3.5 3.0 2.5 2.0 1.5 1.0 0.5 0.0

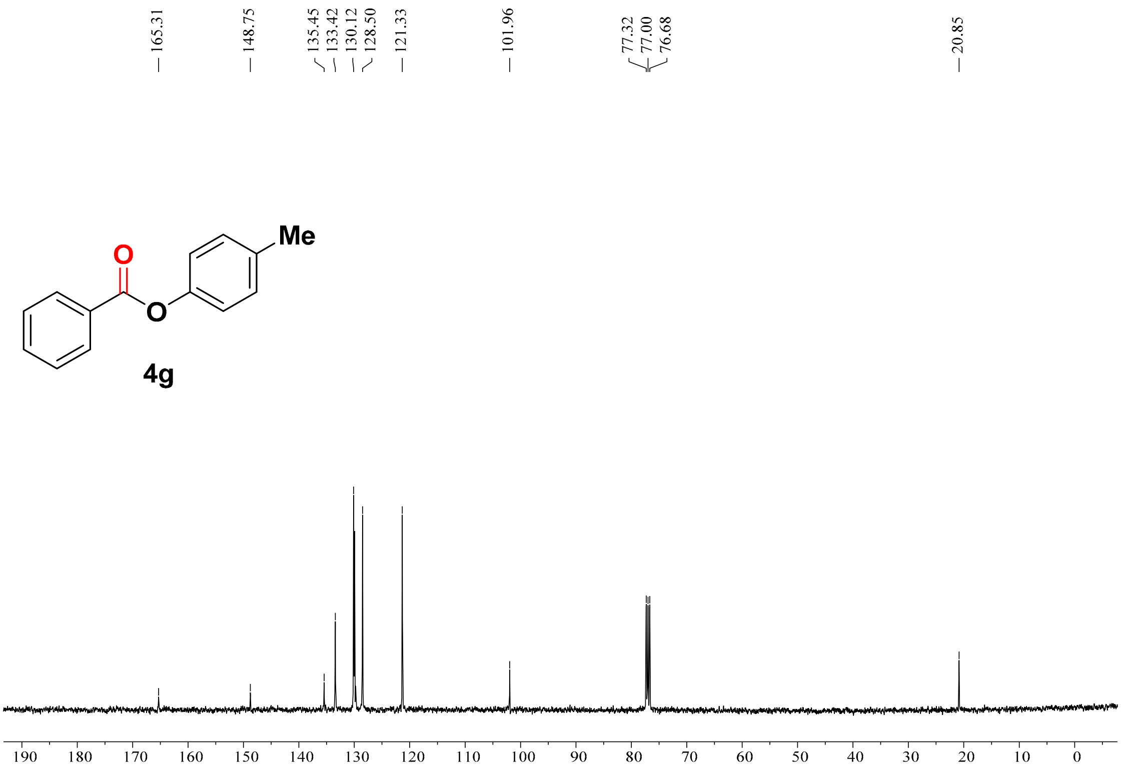

165.31
148.75
135.45
133.42
130.12
128.50
121.33
101.96
77.32
77.00
76.68
20.85
Me
O
O
4g
190 180 170 160 150 140 130 120 110 100 90 80 70 60 50 40 30 20 10 0

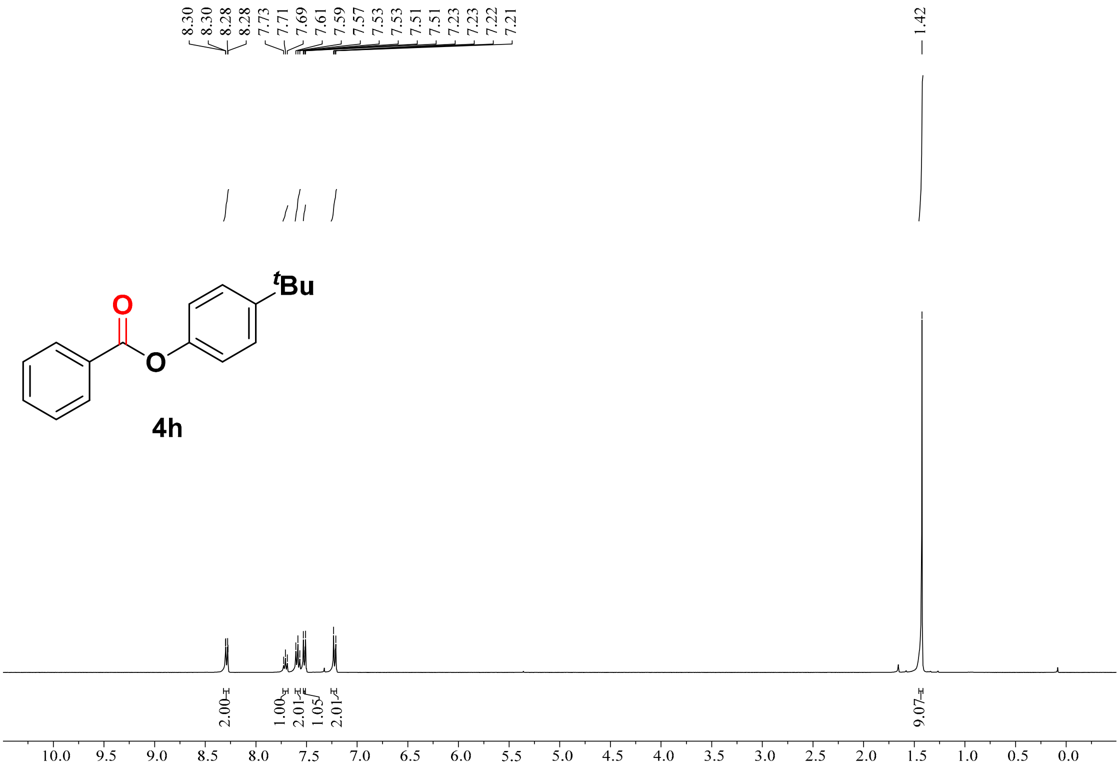
8.30
8.30
8.28
8.28
7.73
7.71
7.69
7.61
7.59
7.57
7.53
7.53
7.51
7.51
7.23
7.23
7.22
7.21
1.42
tBu
O
O
4h
2.00
1.00
2.01
1.05
2.01
9.07
10.0
9.5
9.0
8.5
8.0
7.5
7.0
6.5
6.0
5.5
5.0
4.5
4.0
3.5
3.0
2.5
2.0
1.5
1.0
0.5
0.0

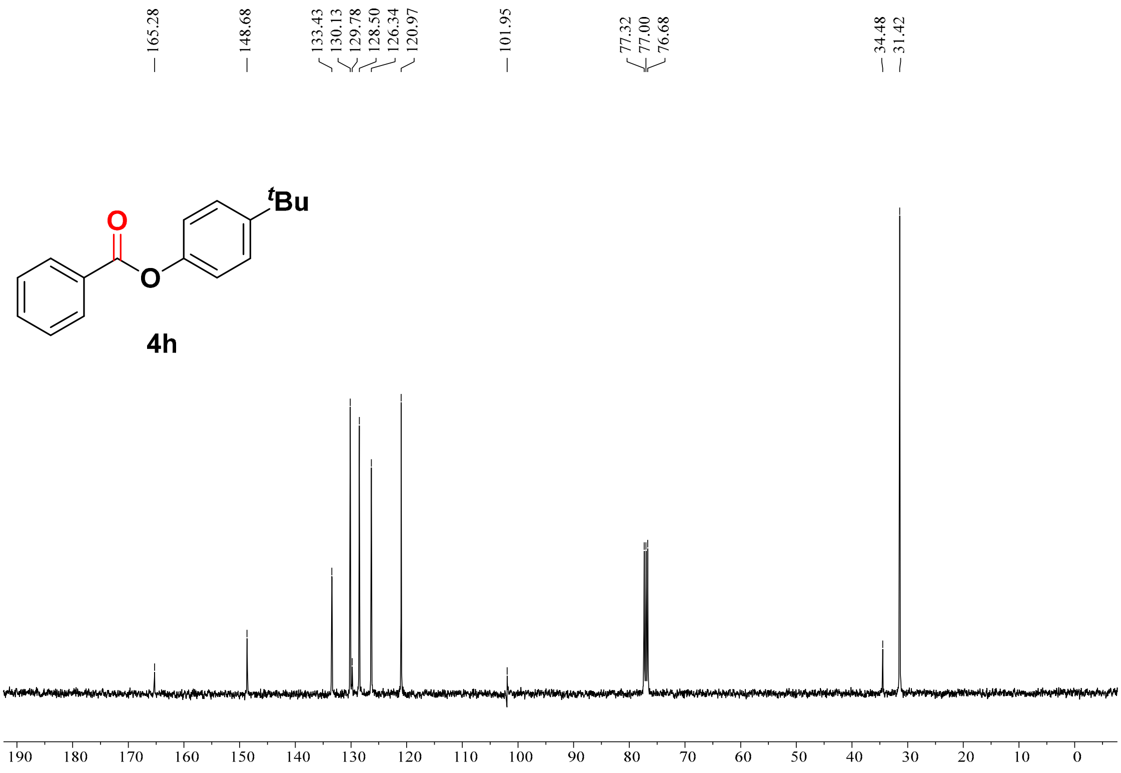
165.28
148.68
133.43
130.13
129.78
128.50
126.34
120.97
101.95
77.32
77.00
76.68
34.48
31.42
tBu
O
O
4h
190
180
170
160
150
140
130
120
110
100
90
80
70
60
50
40
30
20
10
0

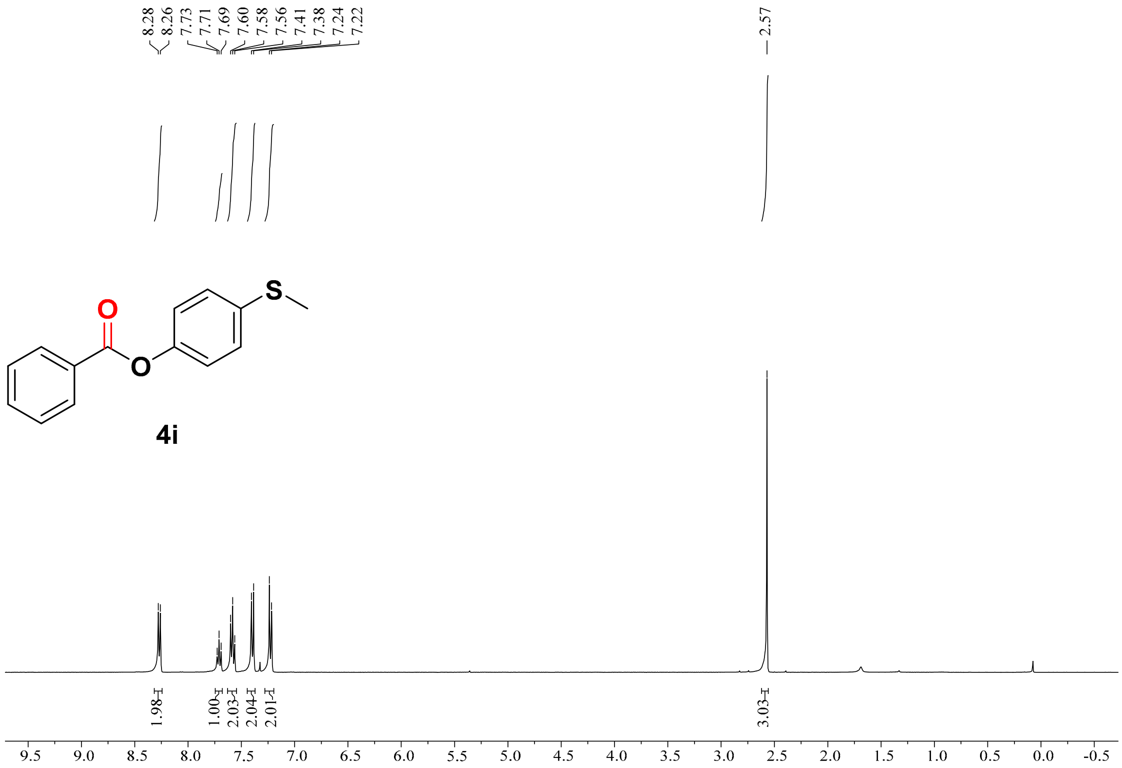
8.28
8.26
7.73
7.71
7.69
7.60
7.58
7.56
7.41
7.38
7.24
7.22
2.57
S
O
O
4i
1.98
1.00
2.03
2.04
2.01
3.03
9.5 9.0 8.5 8.0 7.5 7.0 6.5 6.0 5.5 5.0 4.5 4.0 3.5 3.0 2.5 2.0 1.5 1.0 0.5 0.0 -0.5

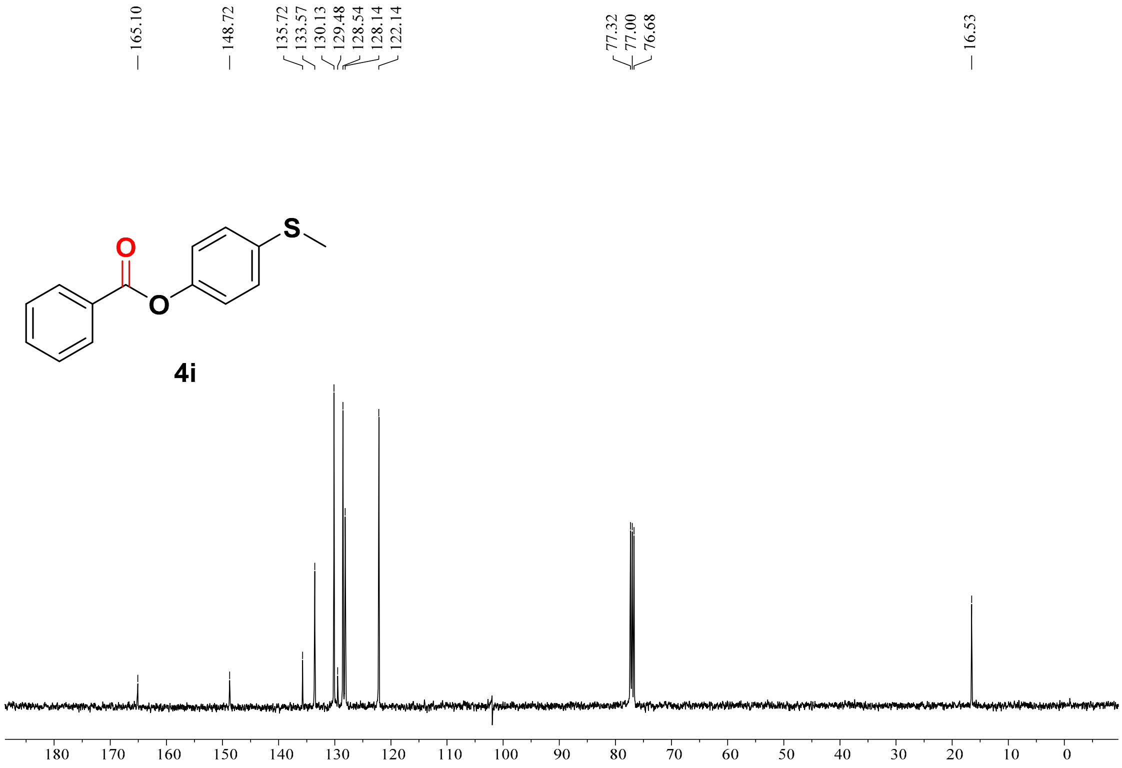
165.10
148.72
135.72
133.57
130.13
129.48
128.54
128.14
122.14
77.32
77.00
76.68
16.53
S
O
O
4i
180 170 160 150 140 130 120 110 100 90 80 70 60 50 40 30 20 10 0

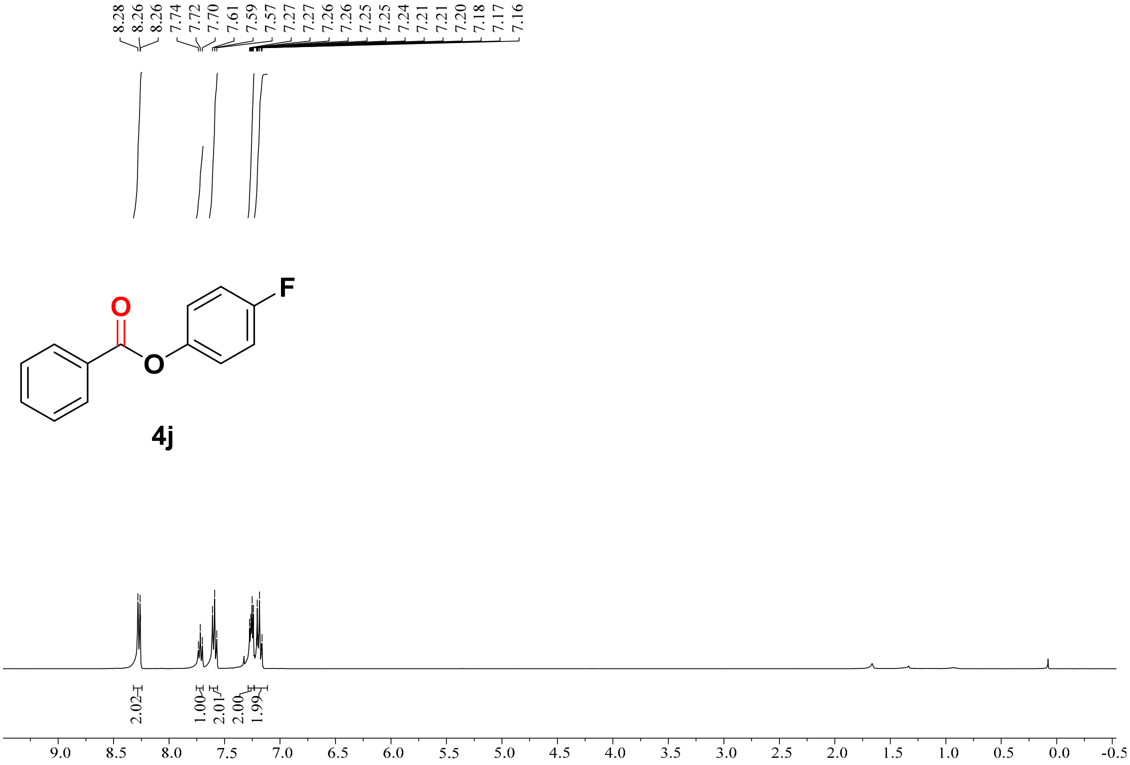
8.28
8.26
8.26
7.74
7.72
7.70
7.61
7.59
7.57
7.27
7.27
7.26
7.26
7.25
7.25
7.24
7.21
7.21
7.20
7.18
7.17
7.16
F
O
O
4j
2.02
1.00
2.01
2.00
1.99
9.0
8.5
8.0
7.5
7.0
6.5
6.0
5.5
5.0
4.5
4.0
3.5
3.0
2.5
2.0
1.5
1.0
0.5
0.0
-0.5

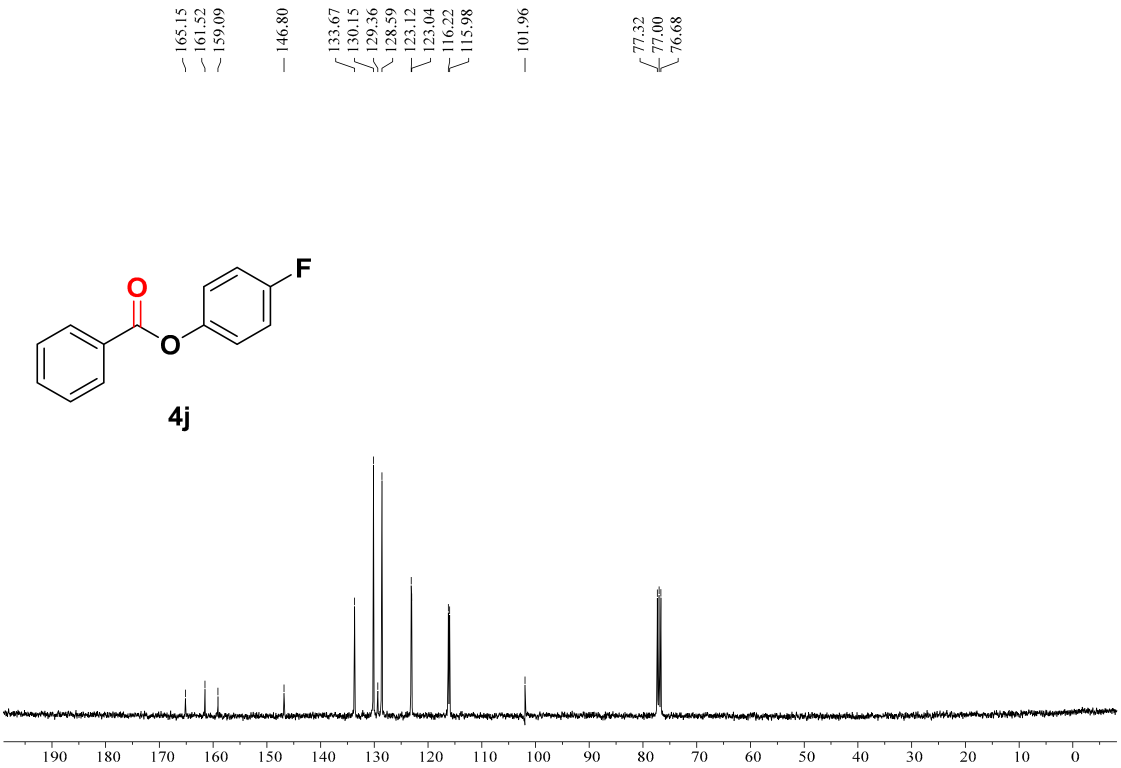
165.15
161.52
159.09
146.80
133.67
130.15
129.36
128.59
123.12
123.04
116.22
115.98
101.96
77.32
77.00
76.68
F
O
O
4j
190
180
170
160
150
140
130
120
110
100
90
80
70
60
50
40
30
20
10
0

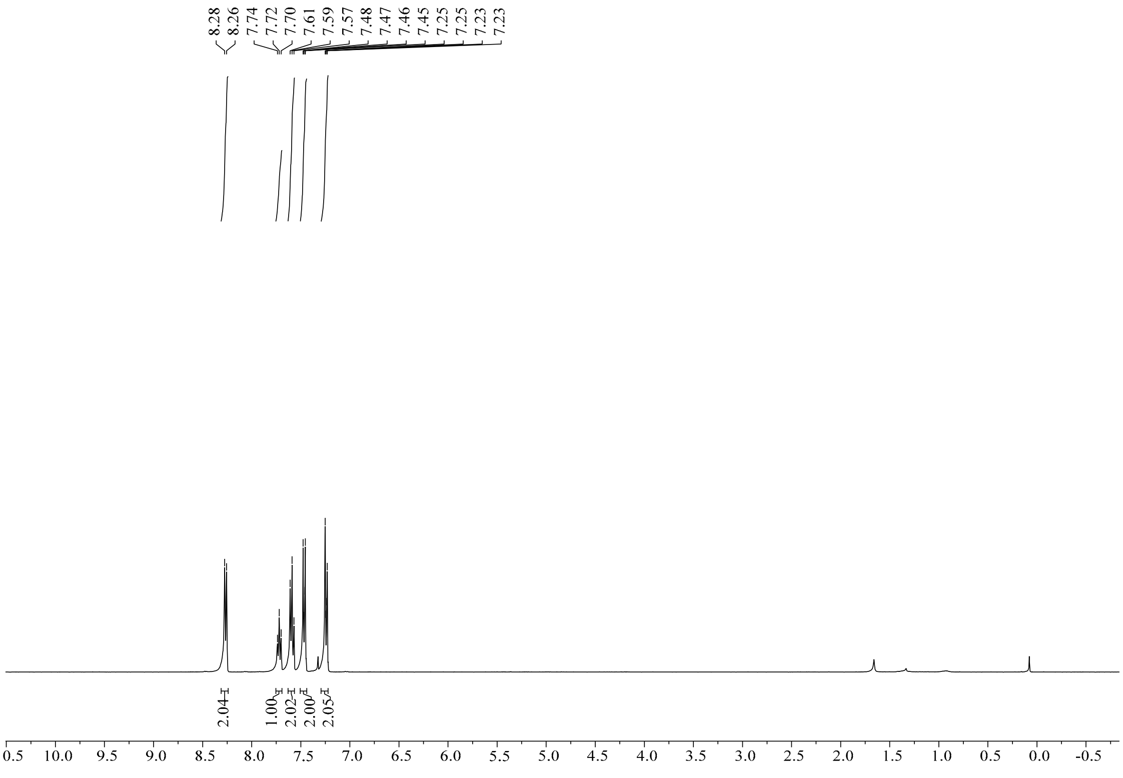

8.28
8.26
7.74
7.72
7.70
7.61
7.59
7.57
7.48
7.47
7.46
7.45
7.25
7.25
7.23
7.23
2.04
1.00
2.02
2.00
2.05
10.5 10.0 9.5 9.0 8.5 8.0 7.5 7.0 6.5 6.0 5.5 5.0 4.5 4.0 3.5 3.0 2.5 2.0 1.5 1.0 0.5 0.0 -0.5

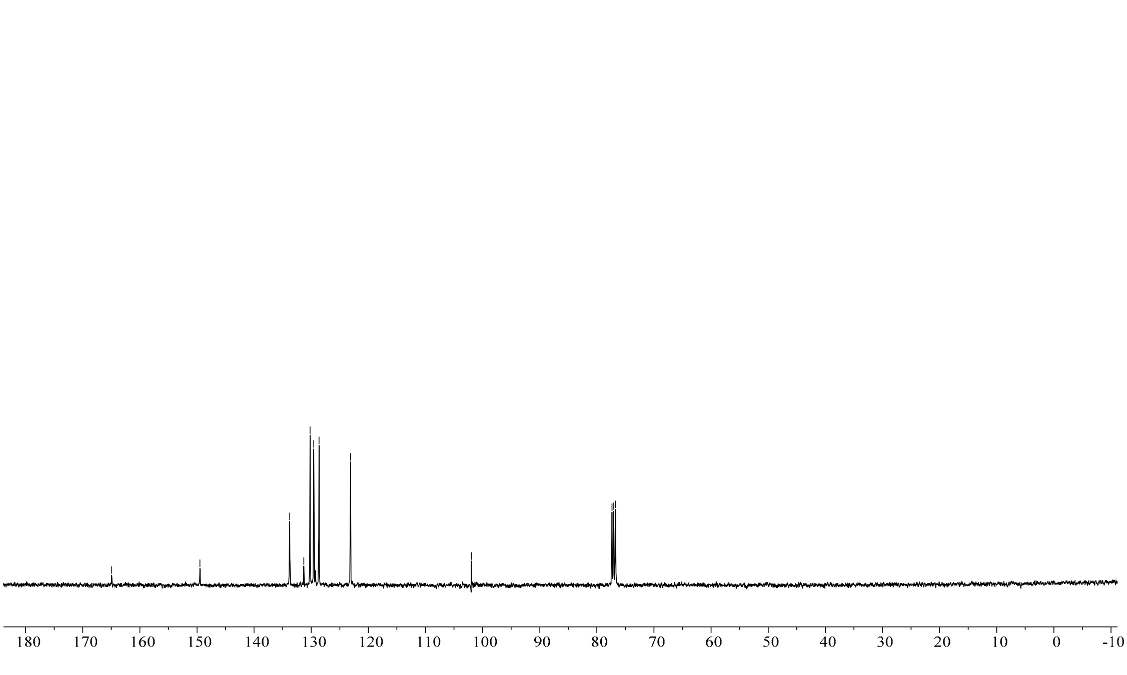

164.92
149.48
133.77
131.29
130.21
129.55
128.64
123.11
101.98
77.36
77.04
76.72
180 170 160 150 140 130 120 110 100 90 80 70 60 50 40 30 20 10 0 -10

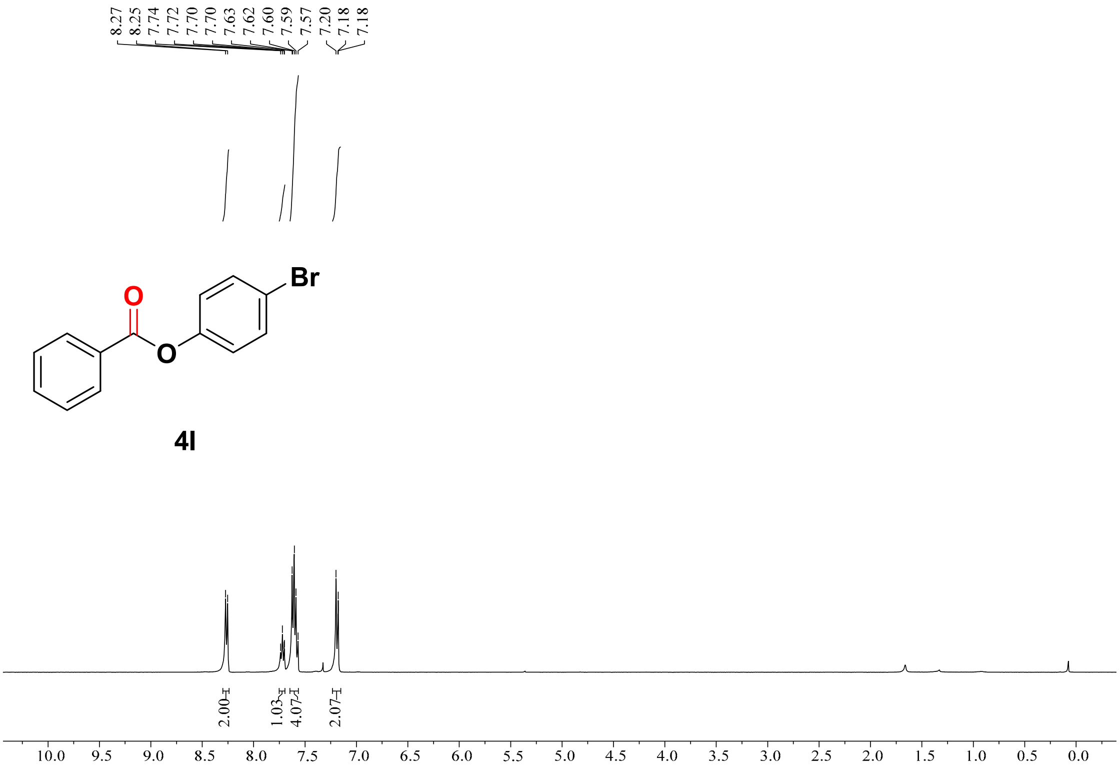
8.27
8.25
7.74
7.72
7.70
7.70
7.63
7.62
7.60
7.59
7.57
7.20
7.18
7.18
Br
O
O
4l
2.00
1.03
4.07
2.07
10.0 9.5 9.0 8.5 8.0 7.5 7.0 6.5 6.0 5.5 5.0 4.5 4.0 3.5 3.0 2.5 2.0 1.5 1.0 0.5 0.0

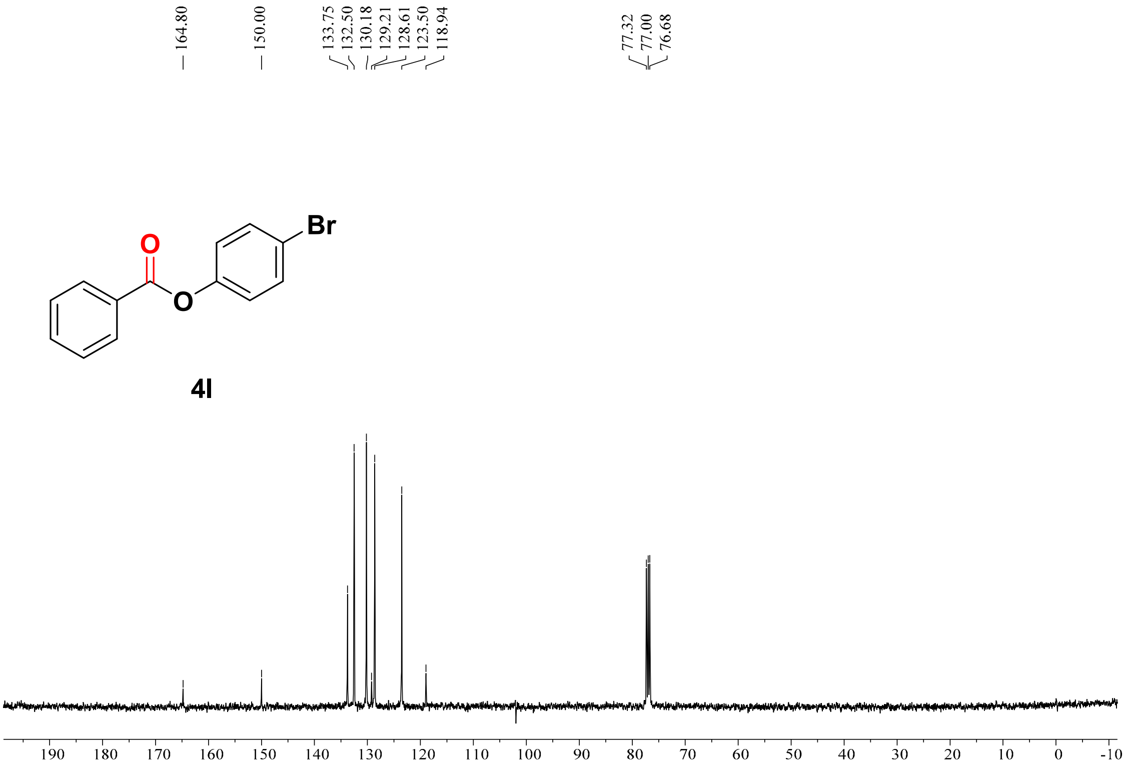
164.80
150.00
133.75
132.50
130.18
129.21
128.61
123.50
118.94
77.32
77.00
76.68
Br
O
O
4l
190 180 170 160 150 140 130 120 110 100 90 80 70 60 50 40 30 20 10 0 -10

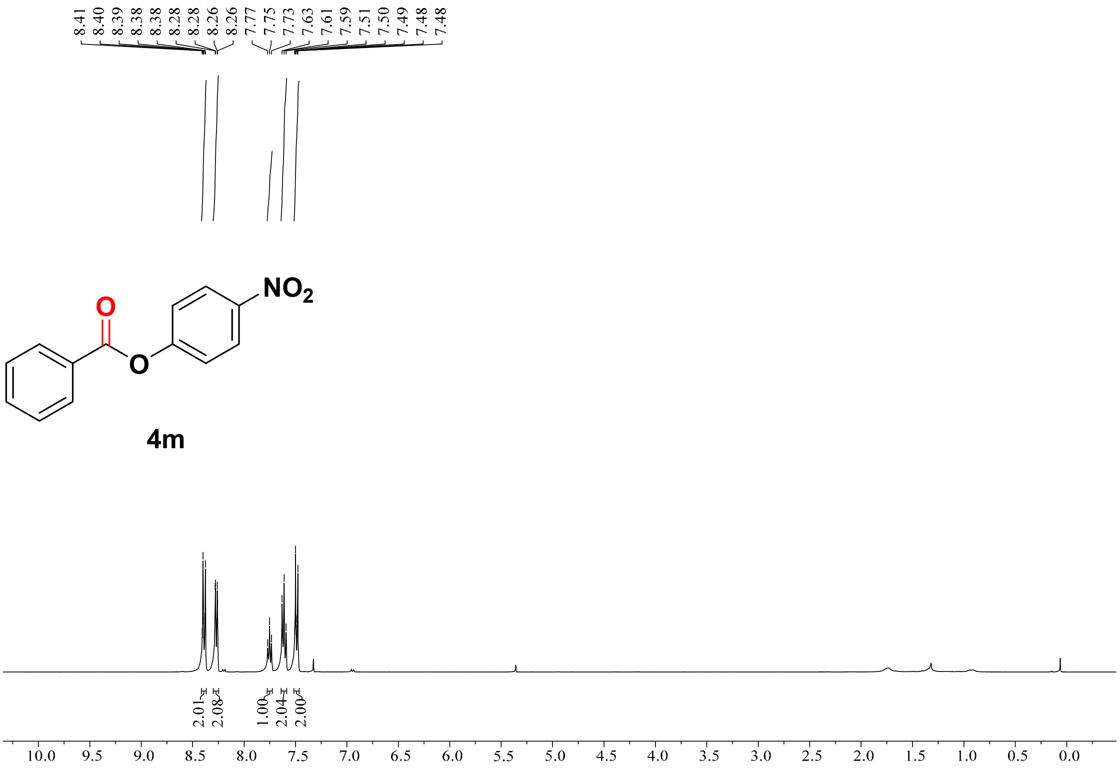
8.41
8.40
8.39
8.38
8.38
8.28
8.28
8.26
8.26
7.77
7.75
7.73
7.63
7.61
7.59
7.51
7.50
7.49
7.48
7.48
NO2
O
O
4m
2.01
2.08
1.00
2.04
2.00
10.0
9.5
9.0
8.5
8.0
7.5
7.0
6.5
6.0
5.5
5.0
4.5
4.0
3.5
3.0
2.5
2.0
1.5
1.0
0.5
0.0

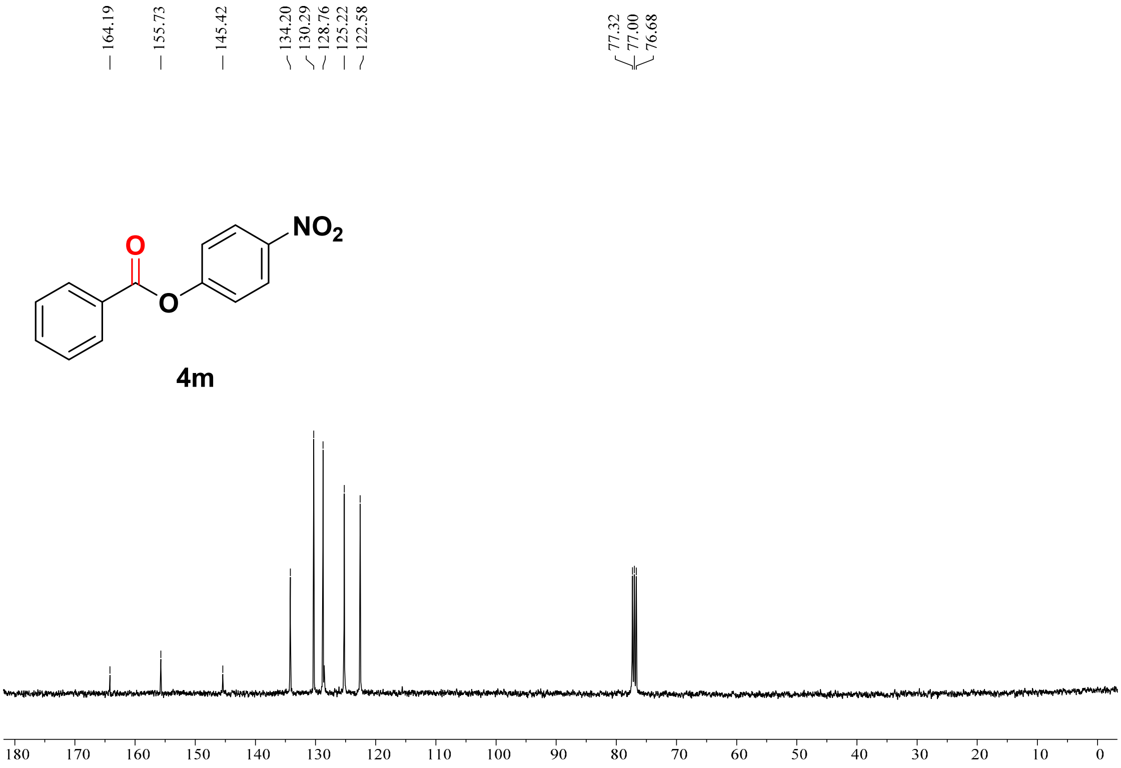
164.19
155.73
145.42
134.20
130.29
128.76
125.22
122.58
77.32
77.00
76.68
NO2
O
O
4m
180
170
160
150
140
130
120
110
100
90
80
70
60
50
40
30
20
10
0

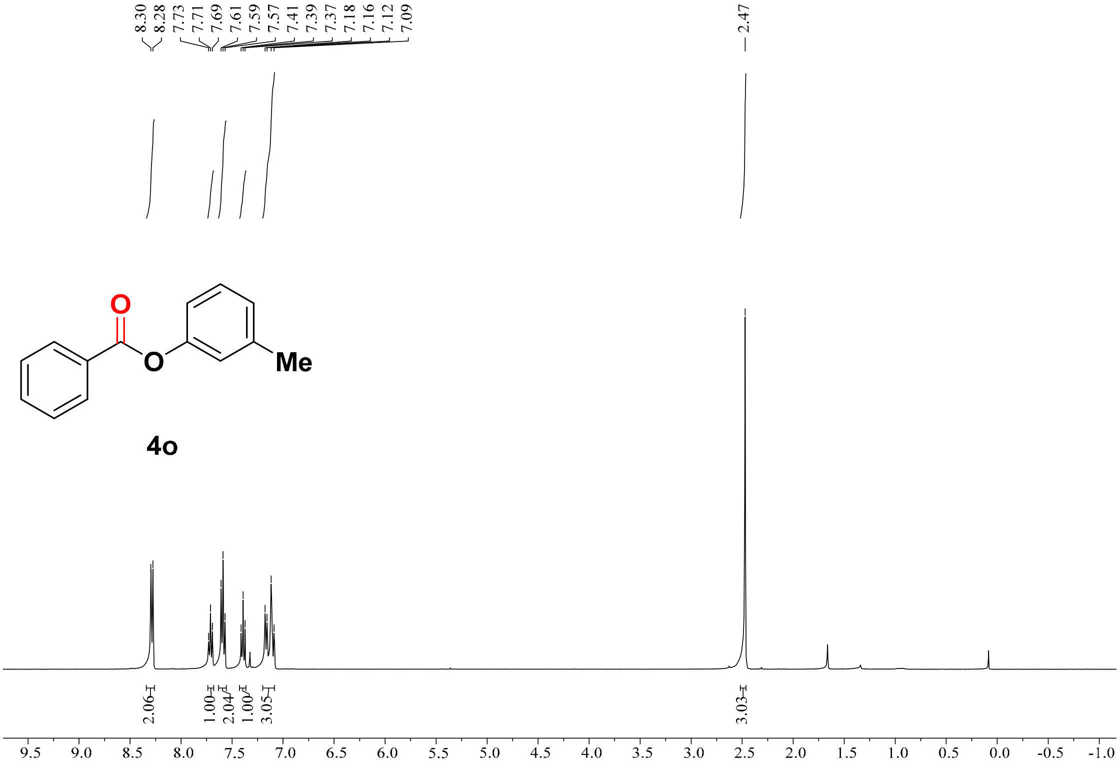
8.30
8.28
7.73
7.71
7.69
7.61
7.59
7.57
7.41
7.39
7.37
7.18
7.16
7.12
7.09
2.47
O
O
Me
4o
2.06
1.00
2.04
1.00
3.05
3.03
9.5 9.0 8.5 8.0 7.5 7.0 6.5 6.0 5.5 5.0 4.5 4.0 3.5 3.0 2.5 2.0 1.5 1.0 0.5 0.0 -0.5 -1.0

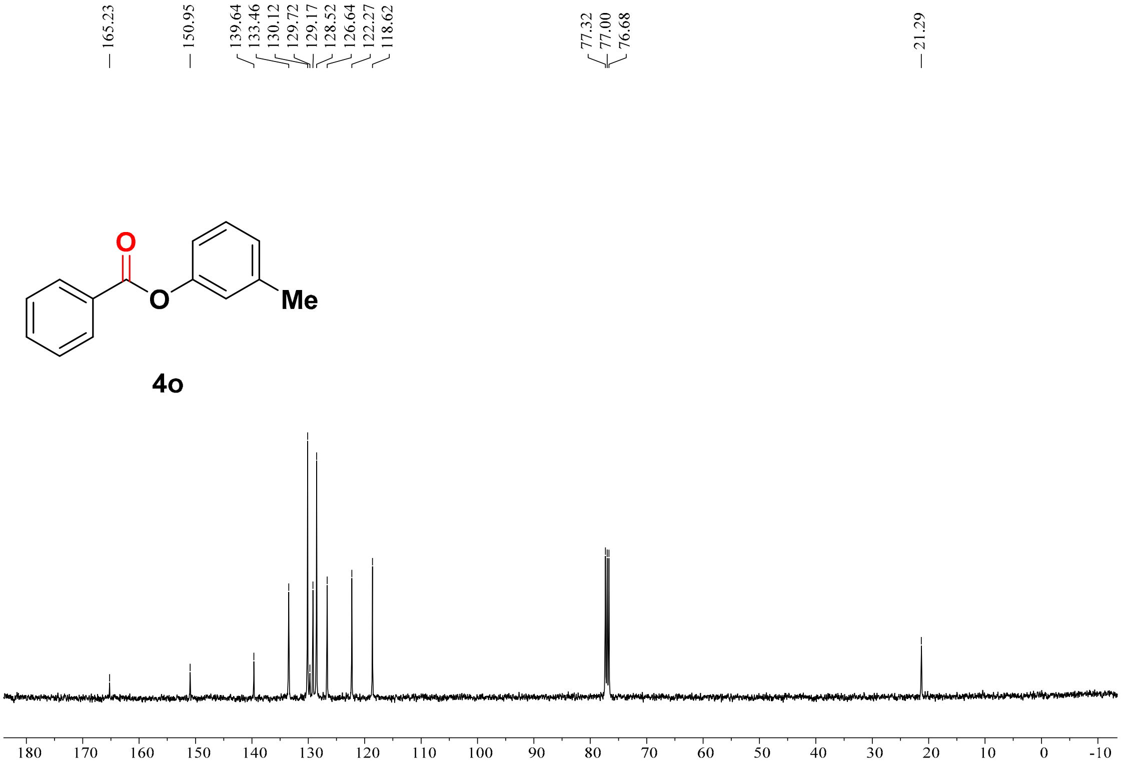
165.23
150.95
139.64
133.46
130.12
129.72
129.17
128.52
126.64
122.27
118.62
77.32
77.00
76.68
21.29
O
O
Me
4o
180 170 160 150 140 130 120 110 100 90 80 70 60 50 40 30 20 10 0 -10
